# Stem cell therapies for periodontal tissue regeneration: a network meta-analysis of preclinical studies

**DOI:** 10.1186/s13287-020-01938-7

**Published:** 2020-10-02

**Authors:** Qiang Li, Guangwen Yang, Jialing Li, Meng Ding, Na Zhou, Heng Dong, Yongbin Mou

**Affiliations:** 1 Department of Oral Implantology, Nanjing Stomatological Hospital, Medical School of Nanjing University, Nanjing, China; 2 Central Laboratory, Nanjing Stomatological Hospital, Medical School of Nanjing University, Nanjing, China; 3 Department of Orthodontics, Nanjing Stomatological Hospital, Medical School of Nanjing University, Nanjing, China

**Keywords:** Stem cell therapy, Periodontitis, Periodontal defects, Periodontal tissue regeneration, Tissue engineering, Network meta-analysis

## Abstract

**Background:**

Periodontal tissue regeneration (PTR) is the ultimate goal of periodontal therapy. Currently, stem cell therapy is considered a promising strategy for achieving PTR. However, there is still no conclusive comparison that distinguishes clear hierarchies among different kinds of stem cells.

**Methods:**

A systematic review and network meta-analysis (NMA) was performed using MEDLINE (via PubMed), EMBASE, and Web of Science up to February 2020. Preclinical studies assessing five types of stem cells for PTR were included; the five types of stem cells included periodontal ligament-derived stem cells (PDLSCs), bone marrow-derived stem cells (BMSCs), adipose tissue-derived stem cells (ADSCs), dental pulp-derived stem cells (DPSCs), and gingival-derived stem cells (GMSCs). The primary outcomes were three histological indicators with continuous variables: newly formed alveolar bone (NB), newly formed cementum (NC), and newly formed periodontal ligament (NPDL). We performed pairwise meta-analyses using a random-effects model and then performed a random-effects NMA using a multivariate meta-analysis model.

**Results:**

Sixty preclinical studies assessing five different stem cell-based therapies were identified. The NMA showed that in terms of NB, PDLSCs (standardized mean difference 1.87, 95% credible interval 1.24 to 2.51), BMSCs (1.88, 1.17 to 2.59), and DPSCs (1.69, 0.64 to 2.75) were statistically more efficacious than cell carriers (CCs). In addition, PDLSCs were superior to GMSCs (1.49, 0.04 to 2.94). For NC, PDLSCs (2.18, 1.48 to 2.87), BMSCs (2.11, 1.28 to 2.94), and ADSCs (1.55, 0.18 to 2.91) were superior to CCs. For NPDL, PDLSCs (1.69, 0.92 to 2.47) and BMSCs (1.41, 0.56 to 2.26) were more efficacious than CCs, and PDLSCs (1.26, 0.11 to 2.42) were superior to GMSCs. The results of treatment hierarchies also demonstrated that the two highest-ranked interventions were PDLSCs and BMSCs.

**Conclusion:**

PDLSCs and BMSCs were the most effective and well-documented stem cells for PTR among the five kinds of stem cells evaluated in this study, and there was no statistical significance between them. To translate the stem cell therapies for PTR successfully in the clinic, future studies should utilize robust experimental designs and reports.

## 1. Introduction

Periodontitis is a chronic bacteriological disease characterized by a series of tooth-supporting structures (gingiva, periodontal ligament, cementum, and alveolar bone) in a state of inflammation, resulting in progressive damage, such as gingival atrophy, alveolar bone resorption, and tooth loss [1]. Currently, the overall prevalence of periodontitis is as high as 45 to 50%, making it the sixth most common human disease and a substantial public health burden worldwide [2]. Periodontitis is also considered to be associated with the occurrence and prognosis of many systemic diseases [3]. Therefore, effective and safe periodontal therapy methods are urgently needed.

The ultimate goal of periodontal therapy is to inhibit periodontitis progression and promote periodontal tissue regeneration (PTR) [4]. Various therapeutic interventions are utilized clinically to treat periodontitis, including removing plaque and calculus by scaling and root planning or removing the necrotic tissues and initiating guided tissue regeneration (GTR) by periodontal surgery [5]. However, it seems that these therapies can only delay tooth loss with a small amount of PTR, which is unsatisfactory for both patients and dentists [6]. To date, the exploration of new PTR therapies includes bone transplantation, allogeneic materials, GTR, and various growth factor-based treatments [6–8]. However, these strategies still cannot reliably regenerate intact periodontal tissue damaged by severe periodontitis [9].

Stem cell-based tissue engineering and regenerative medicine are considered promising treatment strategies for PTR [10–12]. Numerous preclinical studies have tested the feasibility, safety, and effectiveness of various stem cell products [13]. Currently, the most studied stem cells, mainly adipose tissue-derived stem cells (ADSCs) [14, 15], bone marrow-derived stem cells (BMSCs) [16–18], dental pulp-derived stem cells (DPSCs) [19–21], gingival-derived stem cells (GMSCs) [22], and periodontal ligament-derived stem cells (PDLSCs) [23–26], have been assessed for experimental PTR in a variety of animal models [12]. Stem cells have been exploited for their ability to form multiple periodontal tissues under appropriate induction conditions [27]. In addition, the application of stem cells can not only reconstruct the appropriate alveolar bone but also induce newly formed cementum (NC) and periodontal ligament (PDL), which implies complete regeneration of the periodontal complex [28].

Systematic reviews and meta-analyses of preclinical studies can improve the reliability and accuracy of the research results, scientifically support the selection of treatments that should be given priority for clinical trials, and reduce the risk of failure in the transition from animal experiments to clinical trials [29]. Previously, six systematic reviews were conducted using narrative methods without meta-analysis, focusing on a single type of stem cell or the synthesis of multiple stem cells [30–35]. Only three systematic reviews and pairwise meta-analyses assessed the efficacy of stem cells in PTR, demonstrating that stem cell-based therapies generally had a favorable effect [36–38]. However, it is still uncertain whether there are differences in the efficacy of different sources of stem cells in PTR. In addition, these studies were not conclusive enough to generate clear hierarchies.

Hence, the aim of this systematic review and network meta­analysis (NMA) was to comprehensively compare and rank five kinds of stem cells we were interested in for periodontal defect models by evaluating data from published preclinical studies, including ADSCs, BMSCs, DPSCs, GMSCs, and PDLSCs, which will provide reliable evidence for the design of preclinical and clinical trials in the future.

## 2. Methods

This systematic review and NMA was reported according to the PRISMA for Network Meta-Analyses (PRISMA-NMA) statement [39]. The protocol for this study was prepared and registered on the PROSPERO international prospective register of systematic reviews (registration number: CRD42020169202).

### 2.1. Search strategy

An electronic search was performed up to February 2020 in the following electronic bibliographic databases: MEDLINE (via PubMed), EMBASE, and Web of Science. Search strategies were developed for the three databases (See Supplementary Table 1 for details). The retrieval adopted the combination of Mesh terms and free-text terms. The reviewers traced the references of the included literature and relevant systematic reviews in order to supplement the potentially relevant studies manually. Two reviewers (QL and GY) independently performed literature retrieval and screening, with any discrepancies resolved by discussion. There were no language or date restrictions, and foreign language papers were translated if required.

### 2.2. Eligibility criteria

We followed the participants, interventions, comparisons, outcomes, and study (PICOS) design framework [40] to identify and include preclinical, controlled comparative studies of periodontal defects or periodontitis models that evaluated the therapeutic potential of stem cells for the outcomes of three quantitative histological indicators: newly formed alveolar bone (NB), NC, and newly formed PDL (NPDL). Five types of stem cells most commonly used in PTR were included in the present NMA: ADSCs, BMSCs, DPSCs, GMSCs, and PDLSCs. To maintain homogeneity across studies, the review focused on locally applied interventions, excluding systemically applied therapies. We also excluded non-research articles or studies (*e.g.*, reviews, hypotheses, and editorials), studies using animal models accompanied by other diseases, and studies with duplicated data (we evaluated only the latest research). Clinical trials assessing PTR treated with stem cells were also included to summarize existing clinical evidence in a narrative approach.

### 2.3. Data extraction

For each eligible study, two reviewers (QL and GY) independently extracted the following items, when available: study characteristics (author and year of publication); animal model characteristics (species, strain, gender, age or weight, model type, defect size); description of interventions and comparators (stem cell type, cell source, cell passage number, cell number, administration route, administration method, duration, and cell carriers); and outcomes (quantification data of newly formed periodontal tissue, including alveolar bone, cementum, and PDL). When only graphical presentation was available, we used a validated graphical digitizer and open-source program (Web Plot Digitizer, version 4.2, Ankit Rohatgi) to obtain values for mean and standard deviation (SD) or standard error of mean (SEM) under high magnification. When the extracted data existed in the form of medians and quartile differences, we used Hozo’s formulas to transform them into mean and SD [41]. The two reviewers then cross-checked the data and resolved disagreements by discussion with a third reviewer (HD). In case of missing or unclear information, the corresponding authors of the papers were contacted via email.

### 2.4. Quality assessment

Two independent reviewers (QL and GY) evaluated the quality of each included preclinical study and risk of bias with adjudication by a third reviewer (HD), using a modified Systematic Review Centre for Laboratory Animal Experimentation (SYRCLE) risk of bias tool [42]. The tool includes ten defined criteria (Supplementary Table 2), including (1) sequence generation, (2) baseline characteristics, (3) allocation concealment, (4) random housing, (5) blinding against performance bias, (6) random outcome assessment, (7) blinding against detection bias, (8) incomplete outcome data, (9) selective outcome reporting, and (10) other sources of biases. We used “yes,” “no,” and “unclear” to judge the low risk of bias, high risk of bias, and insufficient details reported to assess the risk of bias properly, respectively.

### 2.5. Statistical analysis

For each direct comparison, we did pairwise meta-analyses through Stata software 16.0/MP (StataCorp, College Station, TX). We calculated the standardized mean difference (SMD) for continuous outcomes with 95% credible intervals (CI). Statistical heterogeneity was assessed in each pairwise comparison with the visual inspection of forest plots and *I*^2^ statistic [43].

We performed NMA using the multivariate meta-analysis model through Stata [44]. For this analysis, we used the “network” and “mvmeta” suite of Stata commands [45, 46]. We presented results from NMA as summary relative effect sizes as SMD for each possible pair of treatments. Network diagrams were drawn to depict the evidence for the outcomes. The size of the treatment nodes reflects the number of defects allocated to each treatment, while the thickness of the edges reflects the number of studies informing each comparison. Forest plots and league tables of all pairwise comparisons were prepared to summarize treatment comparisons for each outcome. We estimated the ranking probabilities of each intervention being ranked at different ranking positions and then calculated the surface under the cumulative ranking curve (SUCRA) to summarize the treatment hierarchy [47]. We assumed a common heterogeneity variance in the NMA. The assessment in the entire network was based on the magnitude of the heterogeneity variance parameter (*τ*^2^) estimated from the NMA models. We assessed the statistical inconsistency between direct and indirect sources of evidence globally and locally. To evaluate the presence of inconsistency globally, we used the “design-by-treatment” model as described by Higgins [48]. Using this approach, we judged the presence of inconsistency from any source in the entire network based on a chi-square test. To evaluate the presence of inconsistency locally, we used the node-splitting approach [49] and loop-specific approach [50].

## 3. Results

### 3.1. Description of the included studies

**Fig. 1:**
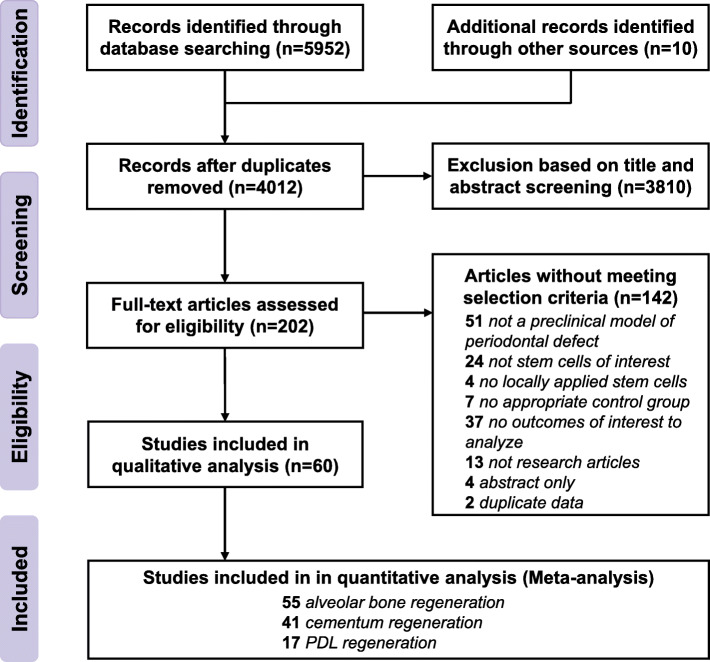
Flowchart of the study selection process according to the PRISMA guidelines. “Other sources” refer to a manual search of bibliographies of the included studies and relevant systematic reviews. Down arrows indicate the progression of studies that passed the previous criteria. Side arrows indicate the number of studies excluded at each stage

The modified PRISMA flowchart in Fig. 1 summarizes the details of the study selection process used to obtain eligible studies [40]. We identified 2252, 1391, and 2309 articles from MEDLINE, EMBASE, and Web of Science databases, respectively. In addition, we also obtained ten potentially eligible studies from relevant reviews. Overall, 4012 articles were identified after eliminating 1950 duplicate articles. Based on the abstract and title reviews, 3810 articles were excluded. After reviewing the full texts of the remaining 202 articles, 60 articles met our eligibility criteria for systematic review and NMA (Supplementary Table 3 shows the references and reasons for exclusion) [15–25, 51–99].

The characteristics of each included study are summarized in Supplementary Table 4 and Supplementary Table 5. Among the 60 preclinical studies, 53 had two arms, six had three arms, and one had four arms. Animal models varied by species, such that 51.67% of the studies used canine models (*e.g.*, beagle dogs and mongrel dogs), 28.33% used rodents (*e.g.*, Sprague Dawley rats, athymic nude rats, and Wistar rats), 16.67% used swine (*e.g.*, minipigs), and 3.33% used ovine. The periodontal defect models included mainly intrabony defects (21.67%), furcation defects (26.67%), and fenestration defects (16.67%). These studies evaluated the effect of stem cells from five different sources for PTR, including PDLSCs (50.00%), BMSCs (33.33%), ADSCs (10.00%), DPSCs (10.00%), and GMSCs (8.33%). Among the 60 studies, 46.67% used autologous stem cells, 38.33% used allogeneic stem cells, and 20.00% used xenogeneic stem cells.

### 3.2. Quality of the included studies

**Fig. 2:**
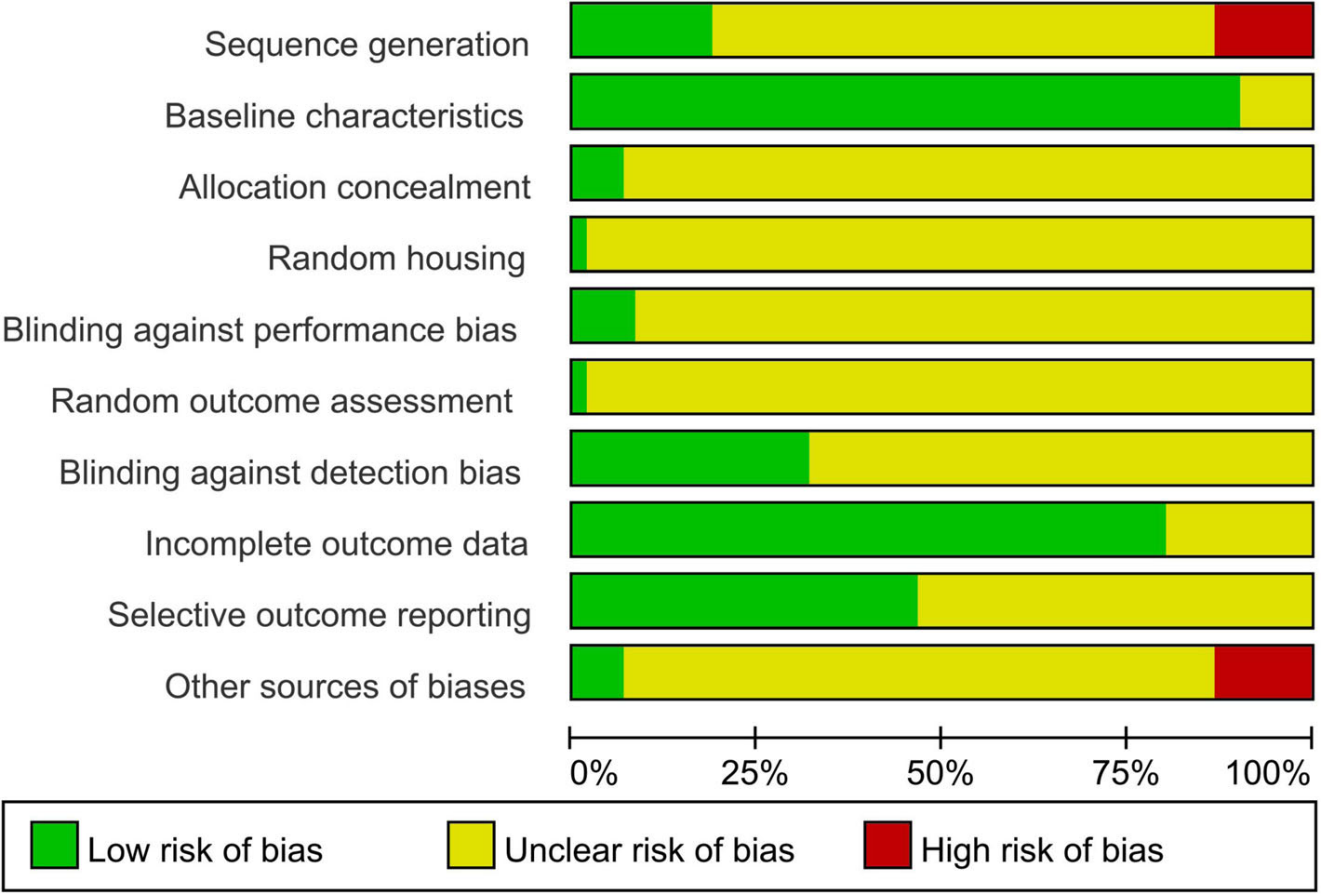
Quality assessment of each risk of bias item presented as percentages across all included studies. The horizontal axis indicates the percentage of answers to the questions in the SYRCLE risk of bias tool. Each color represents a different level of bias: red for a high-risk, green for a low-risk, and yellow for an unclear risk of bias

The SYRCLE risk of bias assessment is shown in Fig. 2 (overall) and Supplementary Fig. 1 (individual studies). Most studies reported the baseline characteristics and avoided attrition bias. Only 20 studies (32.79%) were judged as having a low risk of blinding against detection bias. A high risk of bias was rare in any domain. However, few studies have attempted to report sequence generation, allocation concealment, random housing, blinding against performance bias, random outcome assessment, and other sources of bias; thus, unclear assessments were common.

### 3.3. Outcomes

**Fig. 3:**
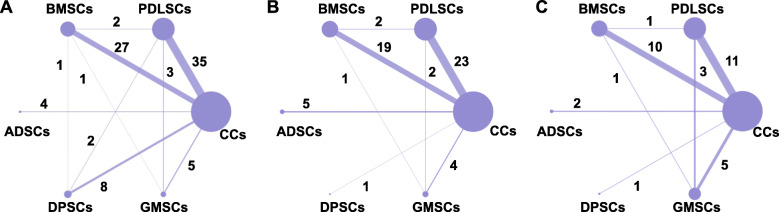
Network plot of comparisons for all studies investigating stem cell therapies for alveolar bone (**a**), cementum (**b**), and periodontal ligament regeneration (**c**). The nodes represent an intervention and their size is proportional to the number of trials comparing this intervention to any other in the network. The lines connecting each pair of interventions represent a direct comparison and are drawn in proportion to the number of trials in each direct comparison. The numbers on the lines represent the number of trials in each comparison

Among the three histological indicators used as study effect sizes in the present NMA, NB was used to illustrate the effect size for stem cell administration in 91.67% of the 60 studies, 68.33% of the studies reported NC and 28.33% of the studies reported NPDL. Figure 3 presents the network plots of each outcome and demonstrates that the two most common comparisons were between PDLSCs and cell carriers (CCs), followed by BMSCs versus CCs.

#### 3.3.1. Pairwise meta-analyses

**Fig. 4:**
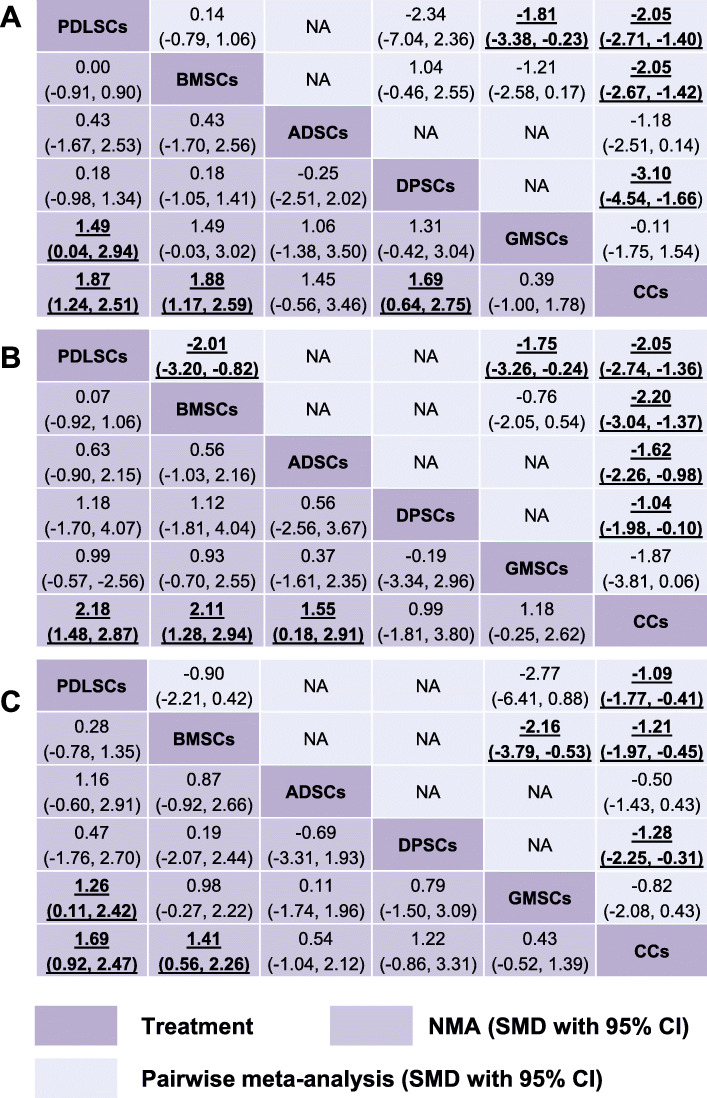
Summary treatment effects from network meta-analysis and pairwise meta-analysis for alveolar bone (**a**), cementum (**b**), and periodontal ligament regeneration (**c**) likelihood. Comparisons should be read from left to right. The estimate is located at the intersection of the column-defining treatment and the row-defining treatment. The lower triangles of the league tables represent the NMA results, and the upper triangles are the pairwise meta-analysis results. For the NMA and pairwise meta-analysis results, an SMD value higher than 0 favors column-defining treatment. For SMDs of comparisons in the opposing direction, the opposite numbers should be taken. Significant results are bold and underscored. NA = not applicable, no preclinical studies making direct comparisons

We present our random-effects pairwise meta-analyses of alveolar bone, cementum, and PDL regeneration in Supplementary Fig. 2 and Fig. 4 (upper triangle). In terms of NB, PDLSCs (SMD 2.05, 95% CI 1.40 to 2.71), BMSCs (SMD 2.05, 95% CI 1.42 to 2.67), and DPSCs (SMD 3.10, 95% CI 1.66 to 4.54) were statistically more efficacious than CCs; PDLSCs (SMD 1.81, 95% CI 0.23 to 3.38) were superior to GMSCs. In terms of NC, PDLSCs (SMD 2.05, 95% CI 1.36 to 2.74), BMSCs (SMD 2.20, 95% CI 1.37 to 3.04), ADSCs (SMD 1.62, 95% CI 0.98 to 2.26), and DPSCs (SMD 1.04, 95% CI 0.10 to 1.98) were statistically more efficacious than CCs; also, PDLSCs were superior to GMSCs (SMD 1.75, 95% CI 0.24 to 3.26) and BMSCs (SMD 2.01, 95% CI 0.82 to 3.20). For NPDL, PDLSCs (SMD 1.09, 95% CI 0.41 to 1.77), BMSCs (SMD 1.21, 95% CI 0.45 to 1.97), and DPSCs (SMD 1.28, 95% CI 0.31 to 2.25) were superior to CCs; and BMSCs (SMD 2.16, 95% CI 0.53 to 3.79) were more efficacious than GMSCs. Notably, the number of studies involving direct comparisons of two types of stem cells is limited (no more than three) (Fig. 3).

#### 3.3.2. Network meta-analyses

**Fig. 5:**
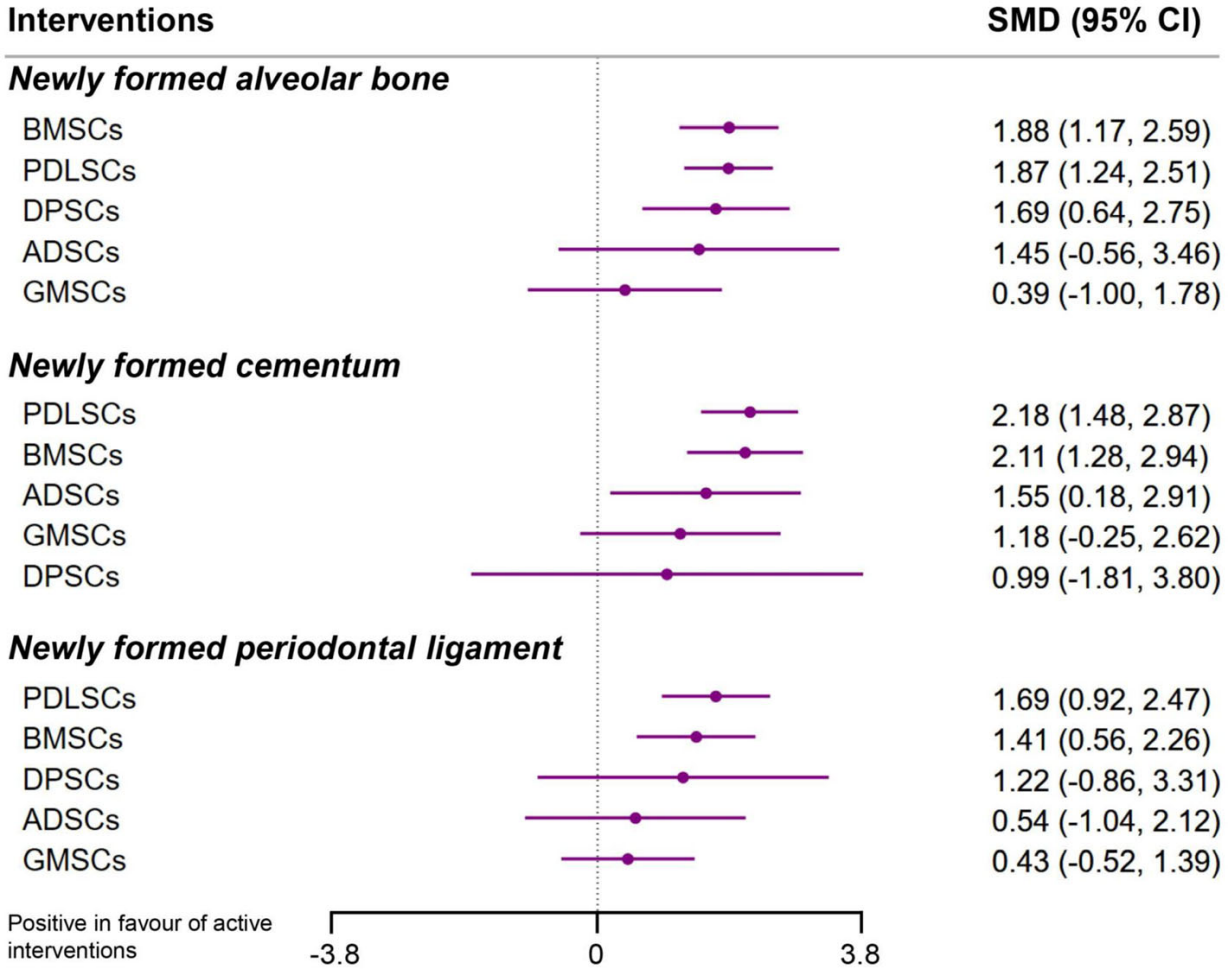
Network meta-analyses of stem cell therapies compared to cell carrier-only controls for each outcome. Points signify SMD estimates, and lines mark their 95% CIs. SMDs of more than 0 favor stem cells over cell carrier-only control. All single 95% CIs crossing the vertical line (0) imply no significant effect on the alveolar bone, cementum, or periodontal ligament regeneration. The SMDs with 95% CIs were estimated from the random-effects consistency model

The lower triangles of Fig. 4 summarize the league tables with SMDs and 95% CIs of interventions compared with each other. The results of the NMAs for all three outcomes are also presented as forest plots in Fig. 5 with estimated SMDs and 95% CIs of interventions compared with a cell carrier-only control. NMA showed that compared with CCs, PDLSCs (SMD 1.87, 95% CI 1.24 to 2.51), BMSCs (SMD 1.88, 95% CI 1.17 to 2.59), and DPSCs (SMD 1.69, 95% CI 0.64 to 2.75) increased NB; PDLSCs showed an increase of 1.49 (95% CI 0.04 to 2.94) in NB compared with GMSCs. In terms of NC, PDLSCs (SMD 2.18, 95% CI 1.48 to 2.87), BMSCs (SMD 2.11, 95% CI 1.28 to 2.94), and ADSCs (SMD 1.55, 95% CI 0.18 to 2.91) were more effective than CCs. Regarding NPDL, trends favored PDLSCs (SMD 1.69, 95% CI 0.92 to 2.47) and BMSCs (SMD 1.41, 95% CI 0.56 to 2.26) when compared to CCs and favored PDLSCs (SMD 1.26, 95% CI 0.11 to 2.42) when compared to GMSCs.

**Fig. 6:**
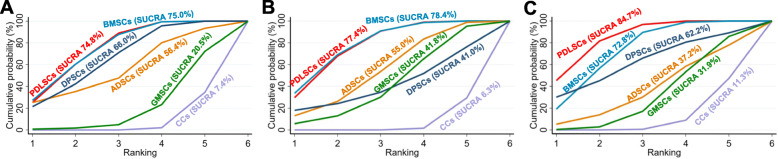
Cumulative ranking plots comparing each of the stem cells for alveolar bone (**a**), cementum (**b**), and periodontal ligament regeneration (**c**). Ranking indicates the cumulative probability of being the best intervention, the second-best intervention, the third-best intervention, etc. The *x*-axis shows the relative ranking, and the *y*-axis shows the cumulative probability of each ranking. The surface underneath this cumulative ranking line (SUCRA) was estimated; the larger the SUCRA, the higher its rank among all available treatments

Figure 6 shows the cumulative probabilities for each intervention being at each possible rank and presents the treatment hierarchies with the SUCRA; the larger the SUCRA is, the higher its rank among all available interventions. Supplementary Table 6 also summarizes the SUCRA, mean probabilities of being best, and mean rank. The ranking in Fig. 6 indicates the cumulative probability of being the best intervention, the second-best intervention, the third-best intervention, etc. A SUCRA value of 100% means the stem cell is the best, and a SUCRA value of 0% means the intervention is the worst. The results of ranking probabilities revealed that the two highest-ranked interventions were PDLSCs and BMSCs in all three outcomes (Fig. 6 and Supplementary Table 6). Specifically, the highest-ranked stem cells in NB were BMSCs (SUCRA 75.0) and PDLSCs (SUCRA 74.8%); DPSCs (SUCRA 66.0%) ranked third followed by ADSCs (SUCRA 56.4%), GMSCs (SUCRA 20.5%), and CCs (SUCRA 7.4%). In terms of NC, BMSCs (SUCRA 78.4%) and PDLSCs (SUCRA 77.4%) ranked two highest followed by ADSCs (SUCRA 55.0%), GMSCs (SUCRA 41.8%), DPSCs (SUCRA 41.0%), and CCs (SUCRA 6.3%). PDLSCs (SUCRA 84.7%) ranked first in PDL regeneration followed by BMSCs (SUCRA 72.8%), DPSCs (SUCRA 62.2%), ADSCs (SUCRA 37.2%), GMSCs (SUCRA 31.9%), and CCs (SUCRA 11.3%).

No evidence of global inconsistency in any outcomes was observed using the “design-by-treatment” models (Supplementary Table 7). The node-splitting approach separated evidence on a particular comparison into direct and indirect evidence. Local inconsistency from the node-splitting model showed significant differences only between CCs vs. PDLSCs and PDLSCs vs. GMSCs for both NC and NPDL but not for NB (Supplementary Table 7). When at least three interventions were compared with each other in a network that formed a closed path, the loop-specific approach compared indirect evidence with direct evidence, and their differences defined the inconsistency factor (IF). Subsequently, the magnitude of the IF, 95% CI of IF, and a loop-specific *z*-test could be used to infer the presence of inconsistency in each loop. The evaluation of inconsistency using loop-specific heterogeneity estimates revealed that there was no inconsistency in each loop (Supplementary Table 7 and Supplementary Fig. 3).

### 3.4. A narrative synthesis of published clinical trials

Among the literature we searched, we finally included eight published clinical trials for narrative synthesis, in which PTR was achieved by using stem cells (Table 1) [100–107]. The types of stem cells used included PDLSCs (three studies), BMSCs (two studies), DPSCs (two studies), and umbilical cord mesenchymal stem cells (UMSCs, one study). Only three studies were designed as randomized controlled trials (RCTs), in which DPSCs [105] and UMSCs [103] were found to have significantly increased PTR compared with that of the control group; although PDLSCs could increase the height of alveolar bone in periodontal defects, no statistically significant differences were detected between the cell group and the control group [102]. Five single-arm studies also demonstrated that PDLSCs, BMSCs, and DPSCs could significantly improve the clinical parameters of periodontal regeneration [100, 101, 104, 106, 107]. Additionally, four studies reported the safety of stem cells, and the results showed that using autologous BMSCs and PDLSCs to treat periodontal defects was safe and did not produce significant adverse effects [101, 102, 104, 106].

**Table 1:** Characteristics of the included clinical trials

Study	Baseline of participants	Defect type	Stem cells	Cell carrier	Control	Major finding	Study design
Aimetti 2018 [100]	11 participants with 11 defects; mean age 51.2 ± 6.1 years; five males and six females; Italia	Intrabony defect	Autologous DPSCs isolated from one vital tooth of the patients	Collagen sponge	Self-control	The application of DPSCs significantly improved clinical (PD, AL) and radiographic parameters (BF) of periodontal regeneration.	Single-arm and single-center clinical study
Baba 2016 [101]	10 participants with ten defects; mean age 48.4 years; three males and seven females; Japan	Intrabony defect	Autologous BMSCs isolated from patient iliac crest marrow aspirate	PRP and a composed of PLA resin fibers	Two healthy teeth per patient were used as the control	All three clinical parameters (PD, AL, and LBG) improved significantly. No clinical safety problems attributable to BMSCs were identified.	Single-arm and single-center clinical study
Chen 2016 [102]	30 participants with 41 defects; 30.04 ± 7.90 years for the control group; 26.05 ± 4.44 for cell group; Male and Female; China	Intrabony defect	Autologous PDLSCs isolated from the third molars of the patients	Bio-oss^®^	21 defects treated with GTR and Bio-oss® without stem cells	Each group showed a significant increase in the alveolar bone height, while no statistically significant differences were detected between the cell group and the control group. Using autologous PDLSCs is safe and does not produce significant adverse effects.	Single-center RCTs
Dhote 2015 [103]	14 participants with 24 defects; mean age 32.62 ± 6.99 years; eight males and six females; India	Intrabony defect	Allogeneic UMSCs isolated from human umbilical cord	β-TCP and rh-PDGF-BB	14 control sites were treated by an open flap debridement only	Using stem cells cultured on β-TCP in combination with rh-PDGF-BB resulted in a significant added benefit in terms of AL gains, PD reductions, more excellent radiographic BF, and improvement in LBG compared to the control group.	Single-center RCTs
Feng 2010 [104]	Three participants with 16 defects; 25, 25, and 29 years; Male; China	Intrabony defect	Autologous PDLSCs obtained from third molars	Bone grafting material CALCITITE 4060-2	Self-control	Clinical examination (PD, AL, and GR) indicated that PDLSCs might provide therapeutic benefits for periodontal defects. All treated patients showed no adverse effects during the follow-up.	Single-arm and single-center clinical study
Ferrarotti 2018 [105]	29 participants with 29 defects; mean age 50.7 ± 8.5 years; 13 males and 14 females; Italia	Intrabony defect	Autologous DPSCs isolated from one vital tooth of the patients	Collagen sponge	14 control sites were filled with collagen sponge alone	Application of DPSCs significantly improved clinical parameters of periodontal regeneration (PD, AL, and BF) 1 year after treatment.	Single-center RCTs
Iwata 2018 [106]	10 participants with 14 defects; mean age 46 ± 12 years; five males and five females; Japan	Intrabony defect	Autologous PDLSCs isolated from the third molars of the patients	β-TCP	Self-control	Clinical parameters (PD, AL) and radiographic assessment (bone height) were improved in all 10 cases at 6 months after the transplantation. These therapeutic effects were sustained during a mean follow-up period of 55 ± 19 months, and there were no serious adverse events.	Single-arm and single-center clinical study
Yamada 2006 [107]	One participant with one defect; 54 years; Female; Japan	Intrabony defect	Autologous BMSCs isolated from patient iliac crest marrow aspirate	PRP and thrombin-calcium chloride	The patient’s contralateral homonymous teeth	BMSCs/PRP gel could be clinically effective in reducing PD, improving AL in intrabony lesions.	Single-arm and single-center clinical study

*Abbreviations*: *AL* attachment level, *BF* bone filling, *β-TCP* beta-tricalcium phosphate, *GR* gingival recession, *LBG* linear bone growth, *PD* probing depth, *PLA* poly-L-lactic acid, *PRP* platelet-rich plasma, *RCTs* randomized controlled trials, *rh-PDGF-BB* recombinant human platelet-derived growth factor-BB

## 4. Discussion

### 4.1. Principal finding

To the best of our knowledge, this systematic review and NMA represents the first and most comprehensive synthesis of data on stem cell-based therapies for preclinical PTR. We mainly focused on five promising stem cell types, among which PDLSCs and BMSCs appeared to be the most effective and the most well-documented stem cell-based therapies for alveolar bone, cementum, and PDL. In addition, direct and indirect comparisons revealed that DPSCs were more efficacious than CCs in terms of bone regeneration and that ADSCs were superior to CCs in terms of cementum regeneration. No apparent inconsistency between the results of direct comparison and indirect comparison was found. However, although a broad range of stem cells had been assessed, there was an unclear risk of bias, and few head-to-head comparisons were performed.

### 4.2. Implications for future preclinical and clinical research

#### 4.2.1. Accessibility of stem cells

In addition to effectiveness, another critical issue is the accessibility of stem cells. The treatment of periodontal defects requires numerous cells (5 × 10^4^ to 2 × 10^8^ cells per defect), which is sometimes difficult to obtain from a single patient. Although stem cells can be expanded in vitro, their ability to self-renew and proliferate is typically diminished during passage [108]. In addition, periodontitis is not a terminal disease and treatment should balance risks and benefits simultaneously. Therefore, the best approach to acquiring stem cells should meet the requirements of sufficient quantity, painless and straightforward sampling, and low risk of complications.

Since PDLSCs and DPSCs are not always conveniently available in clinical practice, it is crucial to develop new sources of stem cells for PTR. Compared with other dental stem cells, GMSCs are easier to obtain, stable in morphology, uniform in morphology, rapid in proliferation, and able to maintain normal karyotype and telomerase activity in long-term culture [109]. However, our NMA showed that there is no evidence that GMSCs are statistically superior to the control group in periodontal regeneration. Nondental stem cells are potential substitutes for dental stem cells. BMSCs are the most widely studied type of stem cell and have proven to be effective in our NMA. ADSCs can be easily isolated in large numbers from resected adipose tissue or by liposuction and show significant similarity with BMSCs in gene expression and osteogenic capacity [24, 110]. Data analyzed in the present NMA also showed that ADSCs are beneficial in cementum regeneration. Induced pluripotent stem cells (iPSCs), another type of stem cell, were not included in our NMA because a few research has evaluated iPSCs and quantitative data are lacking, but the potential curative effect, application value, and potential to avoid ethical issues by using iPSCs cannot be ignored. Both dental cells and nondental cells have been successfully reprogrammed into iPSCs, which can differentiate into multiple cell types [111–114].

#### 4.2.2. Insufficient evidence for other potential stem cells

Our NMA suggests that PDLSCs and BMSCs comprised the largest share (80.00%) of stem cell-based therapies investigated in experimental PTR over the last decade. Both direct and indirect comparisons of PDLSCs and BMSCs concluded a beneficial therapeutic effect in PTR. Clinical studies have also suggested their effectiveness (Table 1) [101, 102, 106, 115]. In contrast, it is worth emphasizing that only a few quantitative analyses of GMSCs and ADSCs were included in the present NMA (five for GMSCs and six for ADSCs). There are differences between the results of different studies, which may be due to the substantial heterogeneity of the studies. For instance, GMSCs were found to be beneficial to alveolar bone regeneration in one out of five studies, while two of three studies showed that GMSCs were beneficial to cementum regeneration, and two of four studies showed that GMSCs favored PDL regeneration (Supplementary Fig. 2). iPSCs, which were reprogrammed from male human foreskin fibroblasts by four transfected transcription factors (Oct4, Sox2, Nanog, and Lin28), demonstrated the ability to initiate alveolar bone, cementum, and PDL regeneration in a periodontal fenestration defect model of rodents [111]. In addition, a recent study has shown that iPSCs, reprogrammed from fibroblasts by transducing retroviral vectors encoding four transcription factors (Oct-4, Sox2, Klf4, and c-Myc), can differentiate into osteocyte-like cells and promote periodontal bone regeneration [112]. Yang et al. [116] improved the regeneration of furcation defects in an experimental periodontitis model by transplanting swine embryonic stem cells (ESCs) and found that the experimental site had differentiated into new PDL and cementum. A clinical trial has shown that the use of UMSCs can significantly improve clinical parameters that reflect periodontal status (Table 1) [103]. Combined with treated dentin matrix particles, cell sheets derived from human dental follicle stem cells (DFSCs) can promote the proliferation and osteogenic differentiation of human BMSCs, and achieve an optimized effect of PTR in beagle dogs with one-wall periodontal intrabony defects [117]. However, due to the limited number of related studies, additional preclinical studies are required in future work to further ensure the efficacy of GMSCs, ADSCs, iPSCs, ESCs, UMSCs, and DFSCs in PTR.

#### 4.2.3. Poorly connected network

Most of the included studies were two-arm trials, and few studies (11.67%) compared stem cells from different sources simultaneously. The evidence map also shows that although PDLSCs and BMSCs are currently the most preclinical studies, there is little evidence for a direct comparison between them, and the whole network diagrams are poorly connected (Fig. 3). Although NMA provides indirect evidence, the evidence intensity of indirect comparisons is weaker than that of direct comparisons, which may lead to deviations in comparison results. Multiple-arm studies or studies providing evidence that addresses key unresolved issues should be performed in the future to make the whole network diagram more complete.

#### 4.2.4. Multiple preclinical animal models

The consequences and implications of the present NMA may be restricted to animal models of periodontal defects and may not be suitable for direct extrapolation to periodontal defects in humans. The differences between preclinical models and human diseases make their relevance to one another debatable.

The development of periodontitis can be divided into different stages, including the formation of pathogenic biofilms, the stimulation/invasion of oral microorganisms and/or their derivatives, the induction of destructive host response in gingival tissue, and the destruction of supporting tissue and alveolar bone [1]. The periodontal defect caused by periodontitis is a chronic inflammatory process in humans. In contrast, the most commonly used modeling methods in the studies included in the present NMA (accounting for 43/60) were the acute defect models created by surgical methods, including surgical dehiscence, fenestration, intrabony, and furcation defects (Supplementary Table 4). Although these models can mimic the defect morphology in humans, they do not exhibit the same microenvironment of chronic inflammation in the defects. In chronic defect models, the formation of natural periodontitis is induced by placing wires, ligatures, or impression material around the teeth. However, the defect areas produced by this method are difficult to standardize. In the included studies, another method was used that overcomes the shortcomings of the above two methods. Standardized defects were created surgically, in which foreign materials (*e.g.*, impression materials, cotton balls saturated with anaerobic bacteria) or binding wires were placed around the affected teeth for a period of time to induce inflammation (Supplementary Table 4).

Multiple animal species, including canines (51.67%), rodents (28.33%), swine (16.67%), and ovine (3.33%), were used in the included studies. Compared with smaller animals, the dental anatomy of larger animals bears a closer resemblance to human dentoalveolar architecture, which allows for a more direct interpretation of the data and the translation of the acquired knowledge into clinical practice [118]. However, due to the issues of expansive, ethics, and the requirement of specialized breeding and maintenance facilities, the use of large animals is limited and should be reserved for the last phase of potential therapy validation [119]. In contrast, small animals (mainly rodents) are often used as a starting point for preliminary screening, and the results are then verified in large animals, which may eliminate the need for larger species before human trials [120].

#### 4.2.5. Biomaterials serve as stem cell delivery vehicles

Our systematic review and NMA findings indicate that stem cells associated with suitable scaffolds may provide beneficial effects on PTR in human patients and preclinical animal models (Table 1 and Supplementary Table 4). As shown in Table 1, clinical trials mainly use scaffolds made of collagen (*e.g.*, fibers, sheets, hydrogels, and sponges) and bone substitute (*e.g.*, Bio-Oss^®^, beta-tricalcium phosphate, and hydroxyapatite), which may be particularly beneficial due to their biocompatibility, biodegradability, and capability to promoting healing [121]. In contrast, the biomaterials used in preclinical experiments are more varied and novel, including but not limited to bone substitute, collagen, polymers, plasma-rich platelets, blood coagulum, enamel matrix derivative, gelatin, and hyaluronic acid (Supplementary Table 4). The diversity of CCs shows the clinical necessity of biomaterials, but it should also be noted that this diversity may lead to substantial heterogeneity in NMA.

The use of appropriate scaffold biomaterials as cell delivery vehicles can not only establish a suitable microenvironment to prolong the viability of stem cells but also provide essential factors to direct stem cell differentiation toward desired lineages [122, 123]. Besides, biomaterials synergize with tissue engineering in recapitulating cellular interactions [124]. In the complex microenvironment of the stem cell/material interface, cells and materials cooperatively dictate each other’s fate: cells reconstruct their surroundings, while materials determine their fate by their inherent properties, such as adhesivity, stiffness, nanostructure, or degradability [123–125]. In addition, stem cells that come into contact with the materials can sense the characteristics of the material, integrate clues through signal propagation, and eventually translate parallel signal information into the decision of cell fate [125]. However, the significant disadvantages of most biomaterials for PTR are poor mechanical strength, weak adhesion to defective tissues, unpredictable cell-biomaterial interactions, immune reaction, low-efficiency cell seeding, and rapid/uncontrollable degradation [35, 126, 127].

The type of defect, especially the number of involved alveolar bone walls, should be considered when choosing the method of cell delivery. When the lesions are retentive, liquid or gel scaffolds can be used without causing the cells to disperse [15, 16, 25, 66]. When the lesions are large, the use of bone substitute can confine the cell-material complex to the surgical site and improve the regeneration process, such as by combining the stem cells and bone substitute or by transplanting the cell sheets attached with polymers to the root surface of the tooth and then filling the bone defects with bone substitute [20, 24, 61, 87].

The treatment of stem cells and biomaterials provides the possibility for substantial PTR. However, although a large number of biomaterials have been widely assessed in a laboratory setting, bone substitutes and collagen membranes are still the most commonly used biomaterials to fill periodontal defects in a clinical setting. Therefore, we suggest that future studies should compare stem cell-based therapies with current clinical conventional periodontal therapies and determine the appropriate type of cell-supporting scaffolds for different defects.

### 4.3. Strengths and limitations of the study

The strength of our study is that we comprehensively compared and ranked five different sources of stem cells for PTR. Compared with previous pairwise meta­analyses, the use of indirect comparisons within the present NMA adds additional information to the current evidence. Compared with periodontal probing and radiological observation techniques used in clinical studies, histology can be used in preclinical studies to analyze regenerative periodontal tissue more intuitively and objectively. Additionally, we included preclinical studies without language restrictions to avoid bias.

Our NMA had some limitations. First, although most of the studies tried to report baseline characteristics and avoid attrition bias, few studies provided enough information to determine how they precluded potential performance bias, detection bias, or reporting bias. Unclear risk of bias was common across studies, which should be considered when interpreting the results from the present NMA. Second, different animal strains, periodontal defect types, and CCs were included in the studies resulting in clinical heterogeneity. Substantial statistical heterogeneity was found in many comparisons of primary outcomes, which might be explained in part by experimental variability. We used a random-effects model to account for the expected heterogeneity. Third, although no evidence of global inconsistency was observed, there is a local inconsistency between the results of direct comparison and indirect comparison, which should be interpreted with caution. In addition, according to past research, funnel plots serving to detect the small-study effects can be severely distorted in pairwise comparisons based on the SMD summary measure [128]. We did not use funnel plots to assess small-study effects in the present NMA of preclinical studies, but it is worth noting that most studies had a small sample size.

## 5. Conclusion

In summary, this systematic review and NMA suggests that in preclinical studies of periodontal defect animal models, PDLSCs and BMSCs have consistently exhibited therapeutic benefits based on quantitative histologic data of NB, NC, and NPDL. PDLSCs have a favorable effect on NB and NPDL compared to GMSCs. Moreover, when compared with cell carrier-only therapies, DPSCs are superior for alveolar bone regeneration, while ADSCs are superior for cementum regeneration. Notably, future preclinical studies should employ robust experimental designs and reporting to overcome the limitations of current studies; such approaches include evaluating the biological rationality and accessibility of given stem cells, reporting adverse reactions, and using standardized models, blinding, and randomization, which may improve the successful translation of stem cell therapies in clinical practice.

## Supplementary information

**Additional file 1.** : Supplementary Table 1. Detailed search strategy.

**Additional file 2.** : Supplementary Table 2. The domain and corresponding questions in the SYRCLE’s risk of bias tool.

**Additional file 3.** : Supplementary Table 3. References and the reasons for the exclusion in the full text reviewing stage.

**Additional file 4.** : Supplementary Table 4. Characteristics of included studies.

**Additional file 5.** : Supplementary Table 5. Summary of the characteristics of included studies.

**Additional file 6.** : Supplementary Fig. 1. Risk of bias of included preclinical studies. Review author’s judgments about each risk of bias item for each included study. +, low risk; −, high risk;?, unclear risk.

**Additional file 7.** : Supplementary Fig. 2. Forest plots of pairwise meta-analyses showing the SMD and 95% CI of alveolar bone, cementum, and periodontal ligament regeneration for each included study. The graphs were generated using the ‘mvmeta’ suite in Stata. For all the plots, the solid vertical line (0) indicates no effect, SMDs of more than 0 favor stem cells on the right side of the x-axis. The size of the box indicates the weighting of each study, and the thin horizontal whisker indicates the 95% CI. The diamond represents overall effect size. Random-effects model was used to summarize the effect sizes. Heterogeneity is denoted by the Ι^2^.

**Additional file 8.** : Supplementary Table 6. Summary of the SUCRA, mean probabilities of being best and mean rank for each outcome.

**Additional file 9.** : Supplementary Table 7. The global and local inconsistency between direct and indirect sources of evidence.

**Additional file 10.** : Supplementary Fig. 3. Evaluation of inconsistency using loop-specific heterogeneity estimates. When at least three interventions are compared with each other in a network that forms a closed path, the loop-specific approach compares indirect evidence with direct evidence, and their differences define the inconsistency factor (IF). The magnitude of the IF, 95% CI of IF, and a loop-specific z-test can be used to infer the presence of inconsistency in each loop. IF close to zero indicates that direct evidence and indirect evidence are very consistent.

## Data Availability

All data generated or analyzed during this study are included in this published article and its supplementary information files.

## Abbreviations

ADSCs: Adipose tissue-derived stem cells
BMSCs: Bone marrow-derived stem cells
CCs: Cell carriers
CI: Credible interval
DFSCs: Dental follicle-derived stem cells
DPSCs: Dental pulp-derived stem cells
ESCs: Embryonic stem cells
GMSCs: Gingival-derived stem cells
GTR: Guided tissue regeneration
IF: Inconsistency factor
iPSCs: Induced pluripotent stem cells
NB: Newly formed bone
NC: Newly formed cementum
NPDL: Newly formed periodontal ligament
PDL: Periodontal ligament
PDLSCs: Periodontal ligament stem cells
PICOS: Participants, interventions, comparisons, outcomes, and study
PTR: Periodontal tissue regeneration
RCT: Randomized controlled trials
SD: Standard deviation
SMD: Standardized mean difference
SUCRA: Surface under the cumulative ranking curve
SYRCLE: Systematic Review Centre for Laboratory Animal Experimentation
UMSCs: Umbilical cord mesenchymal stem cells
