# Supplementary material for: Stem cell therapies for periodontal tissue regeneration: a network meta-analysis of preclinical studies
Source: Stem Cell Res Ther. 2020 Oct 2;11:427. doi: 10.1186/s13287-020-01938-7 (PMC7531120; doi:10.1186/s13287-020-01938-7)
Supplement: Supplementary file 1 — Additional file 1. : Supplementary Table 1. Detailed search strategy. [file 13287_2020_1938_MOESM1_ESM.docx]

**Supplementary Table 1.** **Detailed search strategy**

| Pubmed (Medline) | |
| --- | --- |
| #1 | stem cells [MESH] OR mesenchymal stromal cells [MESH] OR bone marrow cells [MESH] OR stem cell transplantation [MESH] OR regenerative medicine [MeSH] OR progenitor cell*[tiab] OR precursor cell* [tiab] OR embryon* cell* [tiab] OR engineer* [tiab] OR cell engineer* [MESH] OR tissue engineer* [MESH] OR bioengineer* [tiab] OR bio-engineer* [tiab] |
| #2 | Guided tissue regeneration, periodontal [MESH] OR ((periodontium [MESH] OR periodontium* [tiab] OR periodontal [tiab] OR alveolar bone [tiab] OR alveolar ridge [tiab] OR cementum [tiab] OR parodontium [tiab] OR paradentium* [tiab] OR tooth socket* [tiab] OR ((tooth [tiab] OR dental [tiab]) AND (supporting structure* [tiab] OR ligament* [tiab])) OR alveolar bone loss [MESH] OR alveolar atrophy* [tiab] OR alveolar resorption* [tiab] OR periodontal attachment loss [MESH]) AND (regeneration [MESH] OR regenerat* [tiab] OR reconstructive surgical procedures [MESH] OR reconstruct* [tiab] OR reconstitute* [tiab] OR repair* [tiab] OR heal* [tiab] OR rehabilitat* [tiab] OR restor* [tiab])) |
| #1 AND #2 | |
| Embase | |
| #1 | ‘stem cell’/exp OR ‘mesenchymal stem cells’/exp OR ‘bone marrow cell’/exp OR ‘embryo cell’/exp OR ‘cell transplantation’/exp OR ‘cell engineering’/exp OR ‘tissue engineering’/exp OR ‘regenerative medicine’/exp OR ‘tissue culture technique’/exp OR ‘progenitor cell*’:ab,ti OR ‘Bioengineer*’:ab,ti OR ‘Bio-Engineer*’:ab,ti |
| #2 | 'guided tissue regeneration, periodontal'/exp OR ((‘periodontium’/exp OR ‘periodontal ligament’/exp OR ‘alveolar bone’/exp OR ‘alveolar ridge’/exp OR ‘cementum’/exp OR ‘alveolar bone loss’/exp OR ‘periodontium*’:ab,ti OR ‘periodontal’:ab,ti OR ‘parodontium*’:ab,ti OR ‘paradentium*’:ab,ti OR ‘tooth socket*’:ab,ti OR ((‘tooth’:ab,ti OR ‘dental’:ab,ti) AND (‘supporting structure*’:ab,ti OR ‘ligament*’:ab,ti)) OR ‘alveolar atrophy*’:ab,ti OR ‘alveolar resorption*’:ab,ti OR ‘periodontal attachment loss’:ab,ti) AND (‘regenerat*’:ab,ti OR ‘reconstructive surgical procedure*’:ab,ti OR ‘reconstruct*’:ab,ti OR ‘reconstitute*’:ab,ti OR ‘rehabilitat*’:ab,ti)) |
| #1 AND #2 AND [embase]/lim | |
| Web of Science | |
| #1 | TS= (Stem Cell* OR Progenitor Cell* OR Mesenchymal Stromal Cell* OR Mesenchymal Progenitor Cell* OR Wharton* Jelly Cell* OR Progenitor Cell* OR Stem Cell Transplantation OR Cell Engineer* OR Tissue Engineer* OR Tissue-Engineer* OR Tissue Culture Technique* OR Bioengineer* OR Bio-Engineer* OR Regenerative Medicine) |
| #2 | TS= (Guided tissue regeneration, periodontal OR ((periodontium* OR periodontal OR PDL OR alveolar bone OR alveolar ridge OR cementum OR par*d*ntium* OR tooth socket* OR ((tooth OR dental) AND (supporting structure* OR ligament*))) AND (regenerat* OR reconstructive surgical procedure* OR reconstruct* OR reconstitute* OR rehabilitat*))) |
| #1 AND #2, Document type=Article | |
