# Supplementary material for: Stem cell therapies for periodontal tissue regeneration: a network meta-analysis of preclinical studies
Source: Stem Cell Res Ther. 2020 Oct 2;11:427. doi: 10.1186/s13287-020-01938-7 (PMC7531120; doi:10.1186/s13287-020-01938-7)
Supplement: Supplementary file 2 — Additional file 2. : Supplementary Table 2. The domain and corresponding questions in the SYRCLE’s risk of bias tool. [file 13287_2020_1938_MOESM2_ESM.docx]

**Supplementary Table 2. The domain and corresponding questions in the SYRCLE’s risk of bias tool.**

| Item | Domain | Type of bias | Corresponding Questions |
| --- | --- | --- | --- |
| Q1 | Sequence generation | Selection bias | Was the allocation sequence adequately generated and applied? |
| Q2 | Baseline characteristics | Selection bias | Were the groups similar at baseline or were they adjusted for confounders in the analysis? |
| Q3 | Allocation concealment | Selection bias | Was the allocation adequately concealed? |
| Q4 | Random housing | Performance bias | Were animals randomly housed during the experiment? |
| Q5 | Blinding against performance bias | Performance bias | Were the caregivers and/or investigators blinded from knowledge of which intervention each animal received during the experiment? |
| Q6 | Random outcome assessment | Detection bias | Were animals selected at random for outcome assessment? |
| Q7 | Blinding against detection bias | Detection bias | Was the outcome assessor blinded? |
| Q8 | Incomplete outcome data | Attrition bias | Were incomplete outcome data adequately addressed? |
| Q9 | Selective outcome reporting | Reporting bias | Are reports of the study free of selective outcome reporting? |
| Q10 | Other sources of biases | Other | Was the study apparently free of other problems that could result in high risk of bias? |

**Note:** We used "yes", "no", and "unclear" to judge the low risk of bias, high risk of bias, and insufficient details reported to assess the risk of bias properly, respectively.

**Reference:** Hooijmans CR, Rovers MM, de Vries RB, Leenaars M, Ritskes-Hoitinga M, Langendam MW. *SYRCLE's risk of bias tool for animal studies.* BMC medical research methodology. 2014; 14: 43.
