# Supplementary material for: Stem cell therapies for periodontal tissue regeneration: a network meta-analysis of preclinical studies
Source: Stem Cell Res Ther. 2020 Oct 2;11:427. doi: 10.1186/s13287-020-01938-7 (PMC7531120; doi:10.1186/s13287-020-01938-7)
Supplement: Supplementary file 3 — Additional file 3. : Supplementary Table 3. References and the reasons for the exclusion in the full text reviewing stage. [file 13287_2020_1938_MOESM3_ESM.docx]

| Reason of exclusion | Reference | Count |
| --- | --- | --- |
| Not a preclinical model of periodontal defect | (1-51) | 51 |
| Not stem cells of interest | (52-75) | 24 |
| No locally applied stem cells | (76-79) | 4 |
| No appropriate control group | (80-86) | 7 |
| No outcomes of interest to analyze | (87-123) | 37 |
| Not research articles | (124-136) | 13 |
| Abstract only | (137-140) | 4 |
| Duplicate data | (141, 142) | 2 |

**Supplementary Table 3. References and the reasons for the exclusion in the full text reviewing stage.**

**Reference**

1. Ai T, Zhang J, Wang X, Zheng X, Qin X, Zhang Q, et al. DNA methylation profile is associated with the osteogenic potential of three distinct human odontogenic stem cells. Signal Transduct Target Ther. 2018;3:1-.

2. Akazawa K, Iwasaki K, Nagata M, Yokoyama N, Ayame H, Yamaki K, et al. Double-layered cell transfer technology for bone regeneration. Sci Rep. 2016;6:33286-.

3. Alvira-González J, Sánchez-Garcés MÀ, Cairó JRB, Del Pozo MR, Sánchez CM, Gay-Escoda C. Assessment of Bone Regeneration Using Adipose-Derived Stem Cells in Critical-Size Alveolar Ridge Defects: An Experimental Study in a Dog Model. Int J Oral Maxillofac Implants. 2016;31(1):196-203.

4. Cho Y, Kim B, Bae H, Kim W, Baek J, Woo K, et al. Direct Gingival Fibroblast/Osteoblast Transdifferentiation via Epigenetics. J Dent Res. 2017;96(5):555-61.

5. De Kok IJ, Peter SJ, Archambault M, van den Bos C, Kadiyala S, Aukhil I, et al. Investigation of allogeneic mesenchymal stem cell-based alveolar bone formation: preliminary findings. Clin Oral Implants Res. 2003;14(4):481-9.

6. Fang D, Seo BM, Liu Y, Sonoyama W, Yamaza T, Zhang C, et al. Transplantation of mesenchymal stem cells is an optimal approach for plastic surgery. Stem Cells. 2007;25(4):1021-8.

7. He H, Yu J, Cao J, E L, Wang D, Zhang H, et al. Biocompatibility and osteogenic capacity of periodontal ligament stem cells on nHAC/PLA and HA/TCP scaffolds. Journal of Biomaterials Science, Polymer Edition. 2011;22(1-3):179-94.

8. Hiraga T, Ninomiya T, Hosoya A, Takahashi M, Nakamura H. Formation of bone-like mineralized matrix by periodontal ligament cells in vivo: A morphological study in rats. Journal of Bone and Mineral Metabolism. 2009;27(2):149-57.

9. Hong R, Wang Z, Sui A, Liu X, Fan C, Lipkind S, et al. Gingival mesenchymal stem cells attenuate pro-inflammatory macrophages stimulated with oxidized low-density lipoprotein and modulate lipid metabolism. Arch Oral Biol. 2019;98:92-8.

10. Honjo KI, Yamamoto T, Adachi T, Amemiya T, Mazda O, Kanamura N, et al. Evaluation of a dental pulp-derived cell sheet cultured on amniotic membrane substrate. Bio-Medical Materials and Engineering. 2015;25(2):203-12.

11. Hu Y-c, Liu X, Shen J-j, He J-c, Chen Q-e. Experimental study of canine bone marrow mesenchymal stem cells combined with calcium phosphate cement for repair of mandibular bone defects in Beagle dogs. Shanghai Kou Qiang Yi Xue. 2014;23(4):402-8.

12. Huojia M, Wu Z, Zhang X, Maimaitiyiming M, Rong M. Effect of Dental Pulp Stem Cells (DPSCs) in Repairing Rabbit Alveolar Bone Defect. Clin Lab. 2015;61(11):1703-8.

13. Jafarian M, Eslaminejad MB, Khojasteh A, Mashhadi Abbas F, Dehghan MM, Hassanizadeh R, et al. Marrow-derived mesenchymal stem cells-directed bone regeneration in the dog mandible: a comparison between biphasic calcium phosphate and natural bone mineral. Oral Surg Oral Med Oral Pathol Oral Radiol Endod. 2008;105(5):e14-e24.

14. Jahanbin A, Rashed R, Alamdari DH, Koohestanian N, Ezzati A, Kazemian M, et al. Success of Maxillary Alveolar Defect Repair in Rats Using Osteoblast-Differentiated Human Deciduous Dental Pulp Stem Cells. J Oral Maxillofac Surg. 2016;74(4):829.e1-.e8299.

15. Ji B, Sheng L, Chen G, Guo S, Xie L, Yang B, et al. The combination use of platelet-rich fibrin and treated dentin matrix for tooth root regeneration by cell homing. Tissue Eng Part A. 2015;21(1-2):26-34.

16. Kaibuchi N, Iwata T, Onizuka S, Yano K, Yamato M, Okano T, et al. Cytological character of mini pig mesenchymal stromal cells from various tissues and the attempt of cell sheet formation. Regenerative Therapy. 2017;6:83-9.

17. Kawaguchi H, Hayashi H, Mizuno N, Fujita T, Hasegawa N, Shiba H, et al. Cell transplantation for periodontal diseases. A novel periodontal tissue regenerative therapy using bone marrow mesenchymal stem cells. Clin Calcium. 2005;15(7):99-104.

18. Kim J-H, Ko S-Y, Lee JH, Kim D-H, Yun J-H. Evaluation of the periodontal regenerative properties of patterned human periodontal ligament stem cell sheets. J Periodontal Implant Sci. 2017;47(6):402-15.

19. Li H, Sun J, Li J, Yang H, Luo X, Chen J, et al. Xenogeneic Bio-Root Prompts the Constructive Process Characterized by Macrophage Phenotype Polarization in Rodents and Nonhuman Primates. Advanced Healthcare Materials. 2017;6(5).

20. Li J, Xu S-Q, Zhang K, Zhang W-J, Liu H-L, Xu Z, et al. Treatment of gingival defects with gingival mesenchymal stem cells derived from human fetal gingival tissue in a rat model. Stem Cell Res Ther. 2018;9(1):27-.

21. Li J, Zhang F, Zhang N, Geng X, Meng C, Wang X, et al. Osteogenic capacity and cytotherapeutic potential of periodontal ligament cells for periodontal regeneration in vitro and in vivo. PeerJ. 2019;2019(3).

22. Li X, Zheng Y, Zheng Y, Huang Y, Zhang Y, Jia L, et al. Circular RNA CDR1as regulates osteoblastic differentiation of periodontal ligament stem cells via the miR-7/GDF5/SMAD and p38 MAPK signaling pathway. Stem Cell Research and Therapy. 2018;9(1).

23. Ma Y, Ji Y, Zhong T, Wan W, Yang Q, Li A, et al. Bioprinting-Based PDLSC-ECM Screening for in Vivo Repair of Alveolar Bone Defect Using Cell-Laden, Injectable and Photocrosslinkable Hydrogels. ACS Biomaterials Science and Engineering. 2017;3(12):3534-45.

24. Mi HW, Lee MC, Fu E, Chow LP, Lin CP. Highly efficient multipotent differentiation of human periodontal ligament fibroblasts induced by combined BMP4 and hTERT gene transfer. Gene Therapy. 2011;18(5):452-61.

25. Ninomiya T, Hiraga T, Hosoya A, Ohnuma K, Ito Y, Takahashi M, et al. Enhanced bone-forming activity of side population cells in the periodontal ligament. Cell Transplantation. 2014;23(6):691-701.

26. Park JB, Lee K, Lee W, Kim H, Lee KH, Kim I. Establishment of the chronic bone defect model in experimental model mandible and evaluation of the efficacy of the mesenchymal stem cells in enhancing bone regeneration. Tissue Engineering and Regenerative Medicine. 2013;10(1):18-24.

27. Park J-C, Kim J-M, Jung I-H, Kim JC, Choi S-H, Cho K-S, et al. Isolation and characterization of human periodontal ligament (PDL) stem cells (PDLSCs) from the inflamed PDL tissue: in vitro and in vivo evaluations. Journal of clinical periodontology. 2011;38(8):721-31.

28. Park S-Y, Park J-C, Kim M-S, Lee S-E, Kim K-J, Jung B-J, et al. Differential effect of water-soluble chitin on collagen synthesis of human bone marrow stem cells and human periodontal ligament stem cells. Tissue Eng Part A. 2015;21(3-4):451-62.

29. Rubnikovich SP, Denisova YL, Vladimirskaya Т E, Andreyeva VА, Kvacheva ZB, Panasenkova GY, et al. Regenerative cell technologies for gingival recession treatment. Sovremennye Tehnologii v Medicine. 2018;10(4):94-104.

30. Sawada K, Takedachi M, Yamamoto S, Morimoto C, Ozasa M, Iwayama T, et al. Trophic factors from adipose tissue-derived multi-lineage progenitor cells promote cytodifferentiation of periodontal ligament cells. Biochemical and Biophysical Research Communications. 2015;464(1):299-305.

31. Shinagawa-Ohama R, Mochizuki M, Tamaki Y, Suda N, Nakahara T. Heterogeneous Human Periodontal Ligament-Committed Progenitor and Stem Cell Populations Exhibit a Unique Cementogenic Property under in Vitro and in Vivo Conditions. Stem Cells and Development. 2017;26(9):632-45.

32. Song JS, Kim S-O, Kim S-H, Choi H-J, Son H-K, Jung H-S, et al. In vitro and in vivo characteristics of stem cells derived from the periodontal ligament of human deciduous and permanent teeth. Tissue Eng Part A. 2012;18(19-20):2040-51.

33. Sun C, Liu H. Periodontal tissue in a bio-implant by periodontal ligament cells sheet and bone marrow stromal cells sheet. Zhonghua Kou Qiang Yi Xue Za Zhi. 2014;49(2):84-8.

34. Takizawa S, Yamamoto T, Honjo KI, Sato Y, Nakamura K, Yamamoto K, et al. Transplantation of dental pulp-derived cell sheets cultured on human amniotic membrane induced to differentiate into bone. Oral Diseases. 2019;25(5):1352-62.

35. Tansriratanawong K, Tamaki Y, Ishikawa H, Sato S. Co-culture with periodontal ligament stem cells enhances osteogenic gene expression in de-differentiated fat cells. Human Cell. 2014:1-11.

36. Varoni EM, Vijayakumar S, Canciani E, Cochis A, De Nardo L, Lodi G, et al. Chitosan-Based Trilayer Scaffold for Multitissue Periodontal Regeneration. J Dent Res. 2018;97(3):303-11.

37. Wang F, Zhou Y, Zhou J, Xu M, Zheng W, Huang W, et al. Comparison of Intraoral Bone Regeneration with Iliac and Alveolar BMSCs. J Dent Res. 2018;97(11):1229-35.

38. Wang L, Xu W, Chen Y, Wang J. Alveolar bone repair of rhesus monkeys by using BMP-2 gene and mesenchymal stem cells loaded three-dimensional printed bioglass scaffold. Sci Rep. 2019;9(1):18175-.

39. Wang P, Wang W, Geng T, Liu Y, Zhu S, Liu Z, et al. EphrinB2 regulates osteogenic differentiation of periodontal ligament stem cells and alveolar bone defect regeneration in beagles. Journal of Tissue Engineering. 2019;10.

40. Wang Y, Cheung GS-p, Xu X, Zhao S, Zhang C. The effect of cultured autologous periodontal ligament cells on the healing of delayed autotransplanted dog's teeth. J Endod. 2010;36(2):264-7.

41. Wofford A, Bow A, Newby S, Brooks S, Rodriguez R, Masi T, et al. Human Fat-Derived Mesenchymal Stem Cells Xenogenically Implanted in a Rat Model Show Enhanced New Bone Formation in Maxillary Alveolar Tooth Defects. Stem Cells International. 2020;2020.

42. Xu M, Wei X, Fang J, Xiao L. Combination of SDF-1 and bFGF promotes bone marrow stem cell-mediated periodontal ligament regeneration. Bioscience Reports. 2019;39(12).

43. Yang H, Gao L-N, An Y, Hu C-H, Jin F, Zhou J, et al. Comparison of mesenchymal stem cells derived from gingival tissue and periodontal ligament in different incubation conditions. Biomaterials. 2013;34(29):7033-47.

44. Yin X, Li P, Li Y, Cai Y, Wen J, Luan Q. Growth/differentiation factor-5 promotes in vitro/vivo periodontal specific differentiation of induced pluripotent stem cell-derived mesenchymal stem cells. Experimental and Therapeutic Medicine. 2017;14(5):4111-7.

45. Zhang C, Yan B, Cui Z, Cui S, Zhang T, Wang X, et al. Bone regeneration in minipigs by intrafibrillarly-mineralized collagen loaded with autologous periodontal ligament stem cells. Sci Rep. 2017;7(1):10519-.

46. Zhang L, Ding Y, Shao J-L, Dong J-S. Study of alveolar bone defects reparation by combination of bone marrow stromal cells and fibrin glue. Hua Xi Kou Qiang Yi Xue Za Zhi. 2011;29(2):125-8.

47. Zhang L, Wang P, Mei S, Li C, Cai C, Ding Y. In vivo alveolar bone regeneration by bone marrow stem cells/fibrin glue composition. Arch Oral Biol. 2012;57(3):238-44.

48. Zhang Y, Kong N, Zhang Y, Yang W, Yan F. Size-dependent effects of gold nanoparticles on osteogenic differentiation of human periodontal ligament progenitor cells. Theranostics. 2017;7(5):1214-24.

49. Zheng B, Jiang J, Chen Y, Lin M, Du Z, Xiao Y, et al. Leptin Overexpression in Bone Marrow Stromal Cells Promotes Periodontal Regeneration in a Rat Model of Osteoporosis. Journal of periodontology. 2017;88(8):808-18.

50. Zhou Y, Li Y, Mao L, Peng H. Periodontal healing by periodontal ligament cell sheets in a teeth replantation model. Arch Oral Biol. 2012;57(2):169-76.

51. Zhu B, Liu W, Liu Y, Zhao X, Zhang H, Luo Z, et al. Jawbone microenvironment promotes periodontium regeneration by regulating the function of periodontal ligament stem cells. Sci Rep. 2017;7:40088-.

52. Turnbull RS, Freeman E. Use of wounds in the parietal bone of the rat for evaluating bone marrow for grafting into periodontal defects. J Periodontal Res. 1974;9(1):39-43.

53. Levin MP, Getter L, Cutright DE. A comparison of iliac marrow and biodegradable ceramic in periodontal defects. Journal of biomedical materials research. 1975;9(2):183-95.

54. Aukhil I, Pettersson E, Suggs C. Guided tissue regeneration. An experimental procedure in beagle dogs. Journal of periodontology. 1986;57(12):727-34.

55. Iglhaut J, Aukhil I, Simpson DM, Johnston MC, Koch G. Progenitor cell kinetics during guided tissue regeneration in experimental periodontal wounds. J Periodontal Res. 1988;23(2):107-17.

56. Bruckmann C, Walboomers XF, Matsuzaka K, Jansen JA. Periodontal ligament and gingival fibroblast adhesion to dentin-like textured surfaces. Biomaterials. 2005;26(3):339-46.

57. Murano Y, Ota M, Katayama A, Sugito H, Shibukawa Y, Yamada S. Periodontal regeneration following transplantation of proliferating tissue derived from periodontal ligament into class III furcation defects in dogs. Biomedical research (Tokyo, Japan). 2006;27(3):139-47.

58. Nakajima K, Abe T, Tanaka M, Hara Y. Periodontal tissue engineering by transplantation of multilayered sheets of phenotypically modified gingival fibroblasts. J Periodontal Res. 2008;43(6):681-8.

59. Elçin YM, Inanç B, Elçin AE. Human embryonic stem cell differentiation on periodontal ligament fibroblasts. Methods Mol Biol. 2010;584:269-81.

60. Zuolin J, Hong Q, Jiali T. Dental follicle cells combined with beta-tricalcium phosphate ceramic: A novel available therapeutic strategy to restore periodontal defects. Medical Hypotheses. 2010;75(6):669-70.

61. Takamasa K, Wataru K, Takeharu I, Ryoko Y, Masashi O, Hideharu H, et al. Periodontal tissue regeneration with the stem cells cultured conditioned media. Journal of Tissue Engineering and Regenerative Medicine. 2012;6:282.

62. Inukai T, Katagiri W, Yoshimi R, Osugi M, Kawai T, Hibi H, et al. Novel application of growth factors derived from the conditioned media of mesenchymal stem cells (MSC-CM) for periodontal regeneration. Wound Repair and Regeneration. 2014;22(1):A19.

63. Jiawen S, Jianjun Z, Jiewen D, Dedong Y, Hongbo Y, Jun S, et al. Osteogenic differentiation of human amniotic epithelial cells and its application in alveolar defect restoration. Stem Cells Translational Medicine. 2014;3(12):1504-13.

64. Yang H, Aprecio RM, Zhou X, Wang Q, Zhang W, Ding Y, et al. Therapeutic effect of TSG-6 engineered iPSC-derived MSCs on experimental periodontitis in rats: A pilot study. PLoS ONE. 2014;9(6).

65. Han P, Ivanovski S, Crawford R, Xiao Y. Activation of the Canonical Wnt Signaling Pathway Induces Cementum Regeneration. Journal of Bone and Mineral Research. 2015;30(7):1160-74.

66. Su F, Liu S-S, Ma J-L, Wang D-S, E L-L, Liu H-C. Enhancement of periodontal tissue regeneration by transplantation of osteoprotegerin-engineered periodontal ligament stem cells. Stem Cell Res Ther. 2015;6(1):22-.

67. Wang Y, Zhou L, Li C, Xie H, Lu Y, Wu Y, et al. Bone marrow-derived cells homing for self-repair of periodontal tissues: a histological characterization and expression analysis. Int J Clin Exp Pathol. 2015;8(10):12379-89.

68. Wang F, Du L, Ge S. PTH/SDF-1α cotherapy induces CD90+CD34- stromal cells migration and promotes tissue regeneration in a rat periodontal defect model. Sci Rep. 2016;6:30403-.

69. Yu G, Okawa H, Okita K, Kamano Y, Wang F, Saeki M, et al. Gingival Fibroblasts as Autologous Feeders for Induced Pluripotent Stem Cells. J Dent Res. 2016;95(1):110-8.

70. Han N, Zhang F, Li G, Zhang X, Lin X, Yang H, et al. Local application of IGFBP5 protein enhanced periodontal tissue regeneration via increasing the migration, cell proliferation and osteo/dentinogenic differentiation of mesenchymal stem cells in an inflammatory niche. Stem Cell Res Ther. 2017;8(1):210-.

71. Shujaa Addin A, Akizuki T, Hoshi S, Matsuura T, Ikawa T, Fukuba S, et al. Biodegradable gelatin/beta-tricalcium phosphate sponges incorporating recombinant human fibroblast growth factor-2 for treatment of recession-type defects: A split-mouth study in dogs. J Periodontal Res. 2017;52(5):863-71.

72. Chew JRJ, Chuah SJ, Teo KYW, Zhang S, Lai RC, Fu JH, et al. Mesenchymal stem cell exosomes enhance periodontal ligament cell functions and promote periodontal regeneration. Acta Biomaterialia. 2019;89:252-64.

73. He X-T, Li X, Xia Y, Yin Y, Wu R-X, Sun H-H, et al. Building capacity for macrophage modulation and stem cell recruitment in high-stiffness hydrogels for complex periodontal regeneration: Experimental studies in vitro and in rats. Acta biomaterialia. 2019;88:162-80.

74. Zheng Y, Yang Y, Deng Y. Dual therapeutic cobalt-incorporated bioceramics accelerate bone tissue regeneration. Mater Sci Eng C Mater Biol Appl. 2019;99:770-82.

75. Qiu J, Wang X, Zhou H, Zhang C, Wang Y, Huang J, et al. Enhancement of periodontal tissue regeneration by conditioned media from gingiva-derived or periodontal ligament-derived mesenchymal stem cells: a comparative study in rats. Stem Cell Res Ther. 2020;11(1):42-.

76. Liu X, Wang Z, Song W, Sun W, Hong R, Pothukuchi A, et al. Systematically transplanted human gingiva-derived mesenchymal stem cells regulate lipid metabolism and inflammation in hyperlipidemic mice with periodontitis. Experimental and Therapeutic Medicine. 2020;19(1):672-82.

77. Sun W, Wang Z, Xu Q, Sun H, Liu X, Yang J, et al. The treatment of systematically transplanted gingival mesenchymal stem cells in periodontitis in mice. Experimental and Therapeutic Medicine. 2019;17(3).

78. Xu QC, Wang ZG, Ji QX, Yu XB, Xu XY, Yuan CQ, et al. Systemically transplanted human gingiva-derived mesenchymal stem cells contributing to bone tissue regeneration. Int J Clin Exp Pathol. 2014;7(8):4922-9.

79. Yu M, Ge S, Wang F, Wen Y, Yan X, Zeng Q, et al. The role of systemically delivered bone marrow-derived mesenchymal stem cells in the regeneration of periodontal tissues. Int J Oral Maxillofac Implants. 2013;28(6):e503-e11.

80. Chu Q, Wu Z-F, Xie G-Y, Wan L, He H-L, Liu L-X. Effects of gingival fibroblasts transfected with human transform growth factor-beta1 gene on improving the periodontal tissue regeneration. Zhonghua Kou Qiang Yi Xue Za Zhi. 2009;44(5):274-8.

81. Flores MG, Yashiro R, Washio K, Yamato M, Okano T, Ishikawa I. Periodontal ligament cell sheet promotes periodontal regeneration in athymic rats. J Clin Periodontol. 2008;35(12):1066-72.

82. Hou T, Li S, Zhang G, Li Y. High-fluence low-power laser irradiation promotes odontogenesis and inflammation resolution in periodontitis by enhancing stem cell proliferation and differentiation. International Journal of Molecular Medicine. 2018;42(4):2107-19.

83. Lekic PC, Rajshankar D, Chen H, Tenenbaum H, McCulloch CAG. Transplantation of labeled periodontal ligament cells promotes regeneration of alveolar bone. Anatomical Record. 2001;262(2):193-202.

84. Li M, Wang Y, Yu HL, Wang SS, Li B, Yang L. Chinese Journal of Tissue Engineering Research. 2016;20(12):1718-24.

85. Ni C, Zhou J, Kong N, Bian T, Zhang Y, Huang X, et al. Gold nanoparticles modulate the crosstalk between macrophages and periodontal ligament cells for periodontitis treatment. Biomaterials. 2019;206:115-32.

86. Yang M, Gao X, Shen Z, Shi X, Lin Z. Gelatin-assisted conglutination of aligned polycaprolactone nanofilms into a multilayered fibre-guiding scaffold for periodontal ligament regeneration. RSC Advances. 2019;9(1):507-18.

87. Kawaguchi H, Hirachi A, Hasegawa N, Iwata T, Hamaguchi H, Shiba H, et al. Enhancement of periodontal tissue regeneration by transplantation of bone marrow mesenchymal stem cells. Journal of Periodontology. 2004;75(9):1281-7.

88. Seo BM, Miura M, Gronthos S, Bartold PM, Batouli S, Brahim J, et al. Investigation of multipotent postnatal stem cells from human periodontal ligament. Lancet. 2004;364(9429):149-55.

89. Hasegawa N, Kawaguchi H, Hirachi A, Takeda K, Mizuno N, Nishimura M, et al. Behavior of transplanted bone marrow-derived mesenchymal stem cells in periodontal defects. Journal of Periodontology. 2006;77(6):1003-7.

90. Tobita M, Uysal AC, Ogawa R, Hyakusoku H, Mizuno H. Periodontal tissue regeneration with adipose-derived stem cells. Tissue Eng Part A. 2008;14(6):945-53.

91. Iwata T, Yamato M, Zhang Z, Mukobata S, Washio K, Ando T, et al. Validation of human periodontal ligament-derived cells as a reliable source for cytotherapeutic use. Journal of clinical periodontology. 2010;37(12):1088-99.

92. Wei N, Gong P, Liao D, Yang X, Li X, Liu Y, et al. Auto-transplanted mesenchymal stromal cell fate in periodontal tissue of beagle dogs. Cytotherapy. 2010;12(4):514-21.

93. Grimm W-D, Dannan A, Becher S, Gassmann G, Arnold W, Varga G, et al. The ability of human periodontium-derived stem cells to regenerate periodontal tissues: a preliminary in vivo investigation. Int J Periodontics Restorative Dent. 2011;31(6):e94-e101.

94. Park JY, Jeon SH, Choung PH. Efficacy of periodontal stem cell transplantation in the treatment of advanced periodontitis. Cell Transplantation. 2011;20(2):271-85.

95. Tobita M, Mizuno H. Adipose-derived stem cells for periodontal tissue regeneration. Methods Mol Biol. 2011;702:461-70.

96. Wang F, Yu M, Yan X, Wen Y, Zeng Q, Yue W, et al. Gingiva-derived mesenchymal stem cell-mediated therapeutic approach for bone tissue regeneration. Stem Cells and Development. 2011;20(12):2093-102.

97. Wang L, Shen H, Zheng W, Tang L, Yang Z, Gao Y, et al. Characterization of stem cells from alveolar periodontal ligament. Tissue Eng Part A. 2011;17(7-8):1015-26.

98. Wang W-J, Zhao Y-M, Lin B-C, Yang J, Ge L-H. Identification of multipotent stem cells from adult dog periodontal ligament. Eur J Oral Sci. 2012;120(4):303-10.

99. Wen Y, Lan J, Huang H, Yu M, Cui J, Liang J, et al. Application of eGFP to label human periodontal ligament stem cells in periodontal tissue engineering. Arch Oral Biol. 2012;57(9):1241-50.

100. Yang C, Lee J-S, Jung U-W, Seo Y-K, Park J-K, Choi S-H. Periodontal regeneration with nano-hyroxyapatite-coated silk scaffolds in dogs. J Periodontal Implant Sci. 2013;43(6):315-22.

101. Yang J-R, Hsu C-W, Liao S-C, Lin Y-T, Chen L-R, Yuan K. Transplantation of embryonic stem cells improves the regeneration of periodontal furcation defects in a porcine model. Journal of clinical periodontology. 2013;40(4):364-71.

102. Yang SH, Zhong LJ, Zhang PT, Zhang Y, Zhang YM, Ma LP, et al. Autologous periodontal ligament stem cells combined with composites repair periodontal bone defects in miniature pigs. Chinese Journal of Tissue Engineering Research. 2013;17(16):2851-8.

103. Gao Y, Zhao G, Li D, Chen X, Pang J, Ke J. Isolation and multiple differentiation potential assessment of human gingival mesenchymal stem cells. International Journal of Molecular Sciences. 2014;15(11):20982-96.

104. Nakajima R, Ono M, Hara ES, Oida Y, Shinkawa S, Pham HT, et al. Mesenchymal stem/progenitor cell isolation from tooth extraction sockets. J Dent Res. 2014;93(11):1133-40.

105. Sugawara A, Sato S. Application of dedifferentiated fat cells for periodontal tissue regeneration. Human Cell. 2014;27(1):12-21.

106. Padial-Molina M, Rodriguez JC, Volk SL, Rios HF. Standardized in vivo model for studying novel regenerative approaches for multitissue bone-ligament interfaces. Nature Protocols. 2015;10(7):1038-49.

107. Yu N, Bronckers ALJJ, Oortgiesen DAW, Yan X, Jansen JA, Yang F, et al. Periodontal cell implantation contributes to the regeneration of the periodontium in an indirect way. Tissue Engineering - Part A. 2015;21(1-2):166-73.

108. Tang HN, Xia Y, Xu J, Tian BM, Zhang XY, Chen FM. Assessment of cellular materials generated by co-cultured ‘inflamed’ and healthy periodontal ligament stem cells from patient-matched groups. Experimental Cell Research. 2016;346(1):119-29.

109. Wang Z-S, Feng Z-H, Wu G-F, Bai S-Z, Dong Y, Chen F-M, et al. The use of platelet-rich fibrin combined with periodontal ligament and jaw bone mesenchymal stem cell sheets for periodontal tissue engineering. Sci Rep. 2016;6:28126-.

110. Zhang H, Liu S, Zhu B, Xu Q, Ding Y, Jin Y. Composite cell sheet for periodontal regeneration: crosstalk between different types of MSCs in cell sheet facilitates complex periodontal-like tissue regeneration. Stem Cell Research and Therapy. 2016;7(1):1-15.

111. Astuty SW, Sunarto H, Amir L, Idrus E. Evaluation of regenerative therapy using cell sheet through cementum protein-1 expression on macaca nemestrina. International Journal of Applied Pharmaceutics. 2017;9(Special Issue 2):107-9.

112. Basan T, Welly D, Kriebel K, Scholz M, Brosemann A, Liese J, et al. Enhanced periodontal regeneration using collagen, stem cells or growth factors. Front Biosci (Schol Ed). 2017;9:180-93.

113. Shang F, Liu S, Ming L, Tian R, Jin F, Ding Y, et al. Human umbilical cord mscs as new cell sources for promoting periodontal regeneration in inflammatory periodontal defect. Theranostics. 2017;7(18).

114. Zhu B, Liu W, Zhang H, Zhao X, Duan Y, Li D, et al. Tissue-specific composite cell aggregates drive periodontium tissue regeneration by reconstructing a regenerative microenvironment. Journal of Tissue Engineering and Regenerative Medicine. 2017;11(6):1792-805.

115. Zhu M, Miao B, Zhu J, Wang H, Zhou Z. Transplantation of periodontal ligament cell sheets expressing human β‑defensin‑3 promotes anti‑inflammation in a canine model of periodontitis. Molecular medicine reports. 2017;16(5):7459-67.

116. Angelopoulos I, Brizuela C, Khoury M. Gingival Mesenchymal Stem Cells Outperform Haploidentical Dental Pulp-derived Mesenchymal Stem Cells in Proliferation Rate, Migration Ability, and Angiogenic Potential. Cell Transplantation. 2018;27(6):967-78.

117. Shi H, Zong W, Xu X, Chen J. Improved biphasic calcium phosphate combined with periodontal ligament stem cells may serve as a promising method for periodontal regeneration. American Journal of Translational Research. 2018;10(12):4030-41.

118. Wu M, Wang J, Zhang Y, Liu H, Dong F. Mineralization induction of gingival fibroblasts and construction of a sandwich tissue- engineered complex for repairing periodontal defects. Medical Science Monitor. 2018;24:1112-23.

119. Li Y, Nan X, Zhong TY, Li T, Li A. Treatment of Periodontal Bone Defects with Stem Cells from Inflammatory Dental Pulp Tissues in Miniature Swine. Tissue Engineering and Regenerative Medicine. 2019;16(2):191-200.

120. Qiao YQ, Zhu LS, Cui SJ, Zhang T, Yang RL, Zhou YH. Local Administration of Stem Cells from Human Exfoliated Primary Teeth Attenuate Experimental Periodontitis in Mice. Chin J Dent Res. 2019;22(3):157-63.

121. Yang X, Ma Y, Guo W, Yang B, Tian W. Stem cells from human exfoliated deciduous teeth as an alternative cell source in bio-root regeneration. Theranostics. 2019;9(9):2694-711.

122. Zhao Z, Liu J, Schneider A, Gao X, Ren K, Weir MD, et al. Human periodontal ligament stem cell seeding on calcium phosphate cement scaffold delivering metformin for bone tissue engineering. J Dent. 2019;91:103220-.

123. Abedian Z, Jenabian N, Moghadamnia AA, Zabihi E, Pourbagher R, Hossein-Nataj H, et al. A comparative study on immunophenotypic characterization and osteogenic differentiation of human Mesenchymal stromal cells derived from periodontal ligament and gingiva. Journal of periodontology. 2020:10.1002/JPER.19-0535.

124. Zhao Q, Gong P, Tan Z, Yang X. Differentiation control of transplanted mesenchymal stem cells (MSCs): A new possible strategy to promote periodontal regeneration. Medical Hypotheses. 2008;70(5):944-7.

125. He MMH, Zheng MMR, Lin MDY. Bionic restorative system: its potential value in caries therapy. Medical hypotheses. 2009;73(1):60-1.

126. Ishikawa I, Iwata T, Washio K, Okano T, Nagasawa T, Iwasaki K, et al. Cell sheet engineering and other novel cell-based approaches to periodontal regeneration. Periodontol 2000. 2009;51:220-38.

127. Zhang W, Abukawa H, Troulis MJ, Kaban LB, Vacanti JP, Yelick PC. Tissue engineered hybrid tooth-bone constructs. Methods. 2009;47(2):122-8.

128. Yang Z-H, Jin F, Zhang X-J, Liu X, Zhang Y-F, Liu J-Q, et al. A novel possible strategy based on self-assembly approach to achieve complete periodontal regeneration. Artificial organs. 2010;34(7):603-9.

129. Yuan G-H, Yang G-B, Wu L-A, Chen Z, Chen S. Potential Role of Dentin Sialoprotein by Inducing Dental Pulp Mesenchymal Stem Cell Differentiation and Mineralization for Dental Tissue Repair. Dent Hypotheses. 2010;1(2):69-75.

130. Dehghani Nazhavani A, Ghahramani Y, Farhadpour A. Dental pulp stem cells (DPSCS) and regenerative endodontics. Cell Journal. 2011;12:99-100.

131. Akita D, Arai Y, Kaneko T, Yamanaka K, Mashimo T, Morokuma M, et al. Adipose-derived stromal cells as a source in periodontal regeneration. Journal of Tissue Engineering and Regenerative Medicine. 2012;6:7.

132. Menicanin D, Mrozik KM, Wada N, Marino V, Shi S, Bartold PM, et al. Periodontal-ligament-derived stem cells exhibit the capacity for long-term survival, self-renewal, and regeneration of multiple tissue types in vivo. Stem Cells and Development. 2014;23(9):1001-11.

133. Sadeghi D, Nazarian H, Nojehdehian H. Adipose-derived stem cells combined with beta-tricalcium phosphate: A novel possible strategy for periodontal defects regeneration. Medical Hypotheses. 2014;82(1):54-6.

134. Yun J, Kim M, Kim J. Effect of hpdlscs transplantation on periodontal regeneration in rats with ligature-induced periodontitis. Tissue Engineering - Part A. 2017;23:S98.

135. Kim IS, Hwang SJ. Comparison of healing effect on inflammatory bone loss between cultivated human dental pulp stem cells and their conditioned medium concentrate. Calcified Tissue International. 2018;102(1):S64.

136. Sallum EA, Ribeiro FV, Ruiz KS, Sallum AW. Experimental and clinical studies on regenerative periodontal therapy. Periodontol 2000. 2019;79(1):22-55.

137. Adell R. Regeneration of the periodontium. An experimental study in dogs. Scand J Plast Reconstr Surg Suppl. 1974;11:1-177.

138. Wojtowicz A, Chaberek S, Urbanowska E, Ostrowski K. Comparison of efficiency of platelet rich plasma, hematopoieic stem cells and bone marrow in augmentation of mandibular bone defects. N Y State Dent J. 2007;73(2):41-5.

139. Adachi K, Amemiya T, Honjo KI, Ichioka H, Nishigaki M, Oseko F, et al. In vivo investigation of the osteogenic potential of human periodontal ligament cell sheet cultured on amniotic membrane. Journal of Oral and Maxillofacial Surgery. 2014;72(9):e177-e8.

140. Amemiya T, Honjo KI, Adachi K, Nishigaki M, Oseko F, Yamamoto T, et al. Immunohistochemical study of periosteal-derived cell sheet cultured on amniotic membrane aiming at periodontal tissue regeneration. Journal of Oral and Maxillofacial Surgery. 2014;72(9):e176.

141. Li H-X, Yan F-H, Lei L, Liu J-G. Effect of cryopreservation of bone marrow stromal cells on periodontal tissue regeneration. Zhonghua Kou Qiang Yi Xue Za Zhi. 2008;43(4):246-7.

142. Zhou W, Zhao C-H, Mei L-X. Effect of the compound of poly lactic-co-glycolic acid and bone marrow stromal cells modified by osteoprotegerin gene on the periodontal regeneration in Beagle dog periodontal defects. Hua Xi Kou Qiang Yi Xue Za Zhi. 2010;28(3):324-9.
