# Supplementary material for: Stem cell therapies for periodontal tissue regeneration: a network meta-analysis of preclinical studies
Source: Stem Cell Res Ther. 2020 Oct 2;11:427. doi: 10.1186/s13287-020-01938-7 (PMC7531120; doi:10.1186/s13287-020-01938-7)
Supplement: Supplementary file 4 — Additional file 4. : Supplementary Table 4. Characteristics of included studies. [file 13287_2020_1938_MOESM4_ESM.docx]

**Supplementary Table 4 Characteristics of included studies.**

| Study and reference | Species; Strain; Gender; Age/Weight | Defects type; Defects size (mm); (Inflammation-induced) | Transplant type | Stem Cell type; Cell source; Cell passage number; Cell number | Cell carrier | Healing time (weeks); Quantification items |
| --- | --- | --- | --- | --- | --- | --- |
| Akita 2014 (1) | Rodent; F344 rats and SD rats; Male; 8-9 weeks | Periodontal fenestration defects; 2×3×1 | Autologous | ADSCs; Rat; 2~3; 5×10^4^ | PLGA | 5; NB, NC, NPDL |
| Akita 2016 (2) | Rodent; F344 rats; Male; 10 weeks/200±10 g | Periodontal fenestration defects; 2×3×1 | Allogeneic | ADSCs; Rat; 2~3; 1×10^6^ | PLGA | 5; NB, NC, NPDL |
| Akizuki 2005 (3) | Canine; Beagle dogs; Female; 3 years/9.8-11.2 kg | Dehiscence defects; 5×5 | Autologous | PDLSCs; Canine; 4~6; NA | Hyaluronic acid | 8; NB, NC |
| Babo 2016 (4) | Rodent; Nude rats (Hsd:RH-Foxn1rnu); Male; Adult/350-400 g | 3-wall intra-bony defects; 2×2×1.7 | Allogeneic | PDLSCs; Rat; 4; NA | gPL construct overlaid with PL-loaded CaP/PLGA cement | 6; NB, NPDL |
| Cai 2015 (5) | Rodent; Fischer rats (F344/DuCrl); Male; 7 weeks | 3-wall periodontal defects; 2×2×1.7 | Allogeneic | BMSCs; Rat; NA; NA | PLGA/PCL | 6; NB, NPDL |
| Cao 2015 (6) | Swine; Minipigs; Male; 12 months/30-40 kg | 3-wall periodontal defects; 5×7×3 | Xenogeneic | DPSCs; Human; 2~3; 1×10^7^ | NS | 12; NB |
| Dan 2014 (7) | Rodent; CBH-rnu/Arc athymic rats; 12 weeks | Periodontal fenestration defects; 3×1.5 | Xenogeneic | PDLSCs & GMSCs; Human; 5; 1×10^5^ | CaP-PCL | 1 & 4; NB, NC, NPDL |
| Ding 2010 (8) | Swine; Minipigs; Male/Female; 6-8 months/30-40 kg | Periodontal defects; 5×7×3 | Allogeneic & Autologous | PDLSCs; Swine; NA; 1×10^6^ | HA/TCP | 12; NB |
| Doǧan 2002 (9) | Canine; Dogs | Class II furcation defects; 5×2 | Autologous | PDLSCs; Canine; 4; 2×10^5^ | Blood clot | 6; NB, NC |
| Doǧan 2003 (10) | Canine; Dogs; Adult | Class II furcation defects; 5×2 | Autologous | PDLSCs; Canine; 4; 2×10^5^ | Blood clot | 6; NB, NC |
| Du 2014 (11) | Rodent; SD rats; Male; 4-5 weeks/80-100 g | Periodontitis defects; (Binding wire and inoculating them with Pg; 4 weeks) | Allogeneic | BMSCs; Rat; NA; 1×10^6^ | NS | 12; NB |
| Duan 2018 (12) | Rodent; Nude mice; Male; 8 weeks/25-30 g | Periodontal fenestration defects; 4×3×2 | Xenogeneic | PDLSCs; Rat; NA; NA | Collagen membrane /collagen+PRF membrane | 2 & 4; NB |
| Fawzy El-Sayed 2015 (13) | Swine; Minipigs; Male/Female; 5-6 years/50.8±7.2 kg | Periodontal defects; 5×7×3; (Binding 3-0 silk; 4 weeks) | Allogeneic | GMSCs; Swine; 3; 2×10^7^ | HyStem-HP hydrogel | 16; NB, NC |
| Fu 2014 (14) | Swine; Minipigs; Female; 9-12 months/40-45 kg | Periodontitis defects; 5×5×7; (Binding 4-0 silk; 4 weeks) | Allogeneic | PDLSCs & DPSCs; Swine; 3; 1×10^6^ | HA/TCP | 12; NB |
| Guo 2017 (15) | Canine; Beagle dogs; Male; 1 year/10 kg | 2-wall intra-bony defects; 3×3×6; (Binding bacterial plaque retentive 3-0 silk; 4 weeks) | Allogeneic | PDLSCs; Canine; NA; NA | NA | 12; NB, NC |
| Han 2014 (16) | Rodent; SD rats; Female; Adult /220-250 g | Periodontal fenestration defects; 2×3 | Allogeneic | PDLSCs; Rat; NA; 1×10^6^ | Gelfoam | 1 &2 &3 & 4; NB, NC |
| Hu 2016 (17) | Swine; Minipigs; Male; 12 months/30-40 kg | 3-wall intra-bony defects; 5×7×3 | Xenogeneic | DPSCs; Human; 3~4; 1×10^7^ | NS | 12; NB |
| Iwata 2009 (18) | Canine; Beagle dogs; Male; 10 kg | 3-wall infra-bony defects; 5×5×4 | Autologous | PDLSCs; Canine; 3; NA | PGA+β-TCP | 6; NB, NC |
| Jiang 2016 (19) | Canine; Beagle dogs; Male; 6-8 months/13-15 kg | Class II furcation defects; 5×4×3 | Autologous | PDLSCs; Canine; NA; 1×10^6^ | Collagen sponge | 12; NB, NC |
| Khorsand 2013 (20) | Canine; Mongrel dogs; Male; 1–2 years/14-22 kg | Mesial 3-walled periodontal defects; 3×5×8; (Binding 3-0 silk; 4 weeks) | Autologous | DPSCs; Canine; 3; 2×10^7^ | Bio-Oss granules | 8; NB, NC, NPDL |
| Lemaitre 2016 (21) | Rodent; C57BL6/J mice; Female; 8 weeks | Periodontitis defects; (Inject Pg; 4 weeks) | Allogeneic | ADSCs; Mouse; 1; 2×10^5^ | Physiologic serum & type I collagen | 0 & 1 & 6 & 12; NC |
| Li 2009 (22) | Canine; Beagle dogs; Female; Adult/12-18kg | Periodontal fenestration defects; 5×5 | Autologous | BMSCs; Canine; 7; 1×10^5^ | e-PTFE | 8; NB, NC, NPDL |
| Li 2010 (23) | Canine; Beagle dogs; ; 10 kg | Class II furcation defects | Allogeneic | BMSCs; Canine; NA; 3×10^5^ | Collagen membranes | 12; NB, NC |
| Li 2018 (24) | Swine; Minipigs; Male; 18 months/55-60 kg | Periodontitis defects; 5×7×3; (Binding 4-0 silk; 4 weeks) | Xenogeneic | APSCs; Human; 3~5; 2×10^6^ | NS | 12; NB, NC |
| Liu 2008 (25) | Swine; Minipigs; 12 months/30-40 kg | Periodontitis defects; 5×7×3; (Binding 4-0 silk; 4 weeks) | Autologous | PDLSCs; Swine; 3; 2×10^7^ | HA/TCP | 12; NB |
| Liu 2015 (26) | Swine; Minipigs; Female; 9-12 months/40-45 kg | Periodontal defects; 5×7×3; (Binding 4-0 silk) | Xenogeneic | PDLSCs; Human; NA; 2×10^6^ | NS | 12; NB, NC |
| Lu 2004 (27) | Canine; Mongrel dogs; Male; 1-2 years | Class II furcation defects; 3.5×4 | Autologous | PDLSCs; Canine; 2~4; 5×10^6^ | nHAC | 8; NB, NC |
| Ma 2019 (28) | Swine; Minipigs | Periodontal bone defects; 5×7×3 | Xenogeneic | DPSCs & BMSCs; Human; NA; 1×10^7^ | NS | 12; NB |
| Mohammed 2018 (29) | Rodent; Abino Wistar rats; Male; Adult/210-250 g | Periodontitis defects; (Binding 4-0 silk; 2 weeks) | Allogeneic | ADSCs; Rat; NA; 1×10^7^ | PBS | 2 & 4; NB |
| Mrozik 2013 (30) | Ovine; Merino ewes; Female; 3-5 years/63.5-72.0 kg | 0-wall dehiscence periodontal defects; 10 | Allogeneic | PDLSCs; Ovine; 3; 1×10^7^ | Gelfoam | 4; NB, NC, NPDL |
| Nagahara 2015 (31) | Canine; Beagle dogs; Female; 12-20 months/10-14 kg | Class III furcation defects; 4; (Alginate impression materials; 2 weeks) | Autologous | BMSCs; Canine; 3; 2×10^7^ | TCP/atelocollagen | 4 & 8; NB, NC |
| Nakahara 2004 (32) | Canine; Beagle dogs; Female; Adult/10-12 kg | Periodontal fenestration defects; 6×4 | Autologous | PDLSCs; Canine; NA; 3×10^5^ | ePTFE membranes | 4; NB, NC |
| Nuñez 2012 (33) | Canine; Beagle dogs; Male; 1 year/10 kg | 3-wall intra-bony defects; 3×4; (Binding orthodontic wire ligatures; 4 weeks) | Autologous | PDLSCs; Canine; 4~5; 7.5×10^5^ | Collagen scaffold | 12; NB, NC |
| Ozasa 2014 (34) | Canine; Beagle dogs; Female; 50-56 months/9-11 kg | Periodontal furcation defects; 3×4; (Vinyl polysiloxane impression material; 4 weeks) | Autologous | ADSCs; Canine; 3~4; NA | Fibrin gel | 6; NB, NC |
| Paknejad 2015 (35) | Canine; Mongrel dogs; Male; 1-2years/14-22kg | 3-wall intra-bony defects; 4×4 | Autologous | BMSCs; Canine; 3; 2×10^7^ | ABBM | 8; NB, NC, NPDL |
| Park 2011 (36) | Canine; Beagle dogs; 10 months/10 kg | Periodontitis defects; 3×3; (Rubber base impression material; 4 weeks) | Autologous | PDLSCs & DPSCs; Canine; 2~3; 6×10^6^ | NA | 8; NB |
| Rezaei 2019 (37) | Canine; Mongrel dogs; Male; 1 year/20± 5 kg | Class II furcation defects; 5×5×5 | Autologous | BMSCs; Canine; 3; 2×10^6^ | Fibrin glue/PRP+Fibrin glue | 8; NB, NC, NPDL |
| Sano 2020 (38) | Rodent; SD rats; Male; 7 weeks | Periodontal furcation defects; 1.3×1 | Xenogeneic | PDLSCs; Human; <=5; NA | Matrigel | 4 & 8; NB, NC |
| Simsek 2012 (39) | Canine; Mongrel dogs; Adult/15 kg | Class II furcation defects; 5×2; (Rubber base impression material; 3 weeks) | Autologous | BMSCs; Canine; NA; NA | PRP | 8; NB, NC |
| Suaid 2011 (40) | Canine; Beagle dogs; 1.46±0.18 years/10-20 kg | Class II furcation defects; 5×2 | Autologous | PDLSCs; Canine; 2~3; 3×10^5^ | Absorbable membrane | 12; NB, NC, NPDL |
| Suaid 2012 (41) | Canine; Beagle dogs; 1.46 ± 0.18 years/10-20 kg | Class III furcation defects; 5 | Autologous | PDLSCs; Canine; NA; 3×10^5^ | Collagen sponge | 12; NB, NC |
| Takewaki 2017 (42) | Canine; Beagle dogs; Female; 12-20 months | Inflammatory class III furcation defects; 4; (Alginate impression materials; 1 week) | Autologous & Allogeneic | BMSCs; Canine; 3; NA | NA | 8 & 12; NB, NC |
| Tcacencu 2012 (43) | Rodent; SD rats; Male; 350 g | Periodontal fenestration defects; 2×3~4 | Xenogeneic | BMSCs; Human; 4~5; 1.5×10^6^ | Absorbable collagen sponge or peptide hydrogel | 1 & 4; NB |
| Tobita 2013 (44) | Canine; Beagle dogs; 9-10 months/8-10 kg | Class III periodontal tissue defects; 5 | Autologous | ADSCs; Canine; 2; 7.5×10^6^ | PRP | 4 & 8; NB, NC |
| Tsumanuma 2011 (45) | Canine; Beagle dogs; Male; 10 kg | 1-wall intra-bony defects; 5×5 | Autologous | PDLSCs & BMSCs; Canine; 3; 9-15×10^4^ | PGA | 8; NB, NC |
| Tsumanuma 2016 (46) | Canine; Beagle dogs; Male; 1-2 years/10 kg | Supra-alveolar periodontal defects; 6 | Autologous & Allogeneic | PDLSCs; Canine; 5; NA | β-TCP/collagen | 8; NB, NC, NPDL |
| Vaquette 2019 (47) | Ovine; Sheep | Dehiscence periodontal defects; 6×5 | Autologous | GMSCs & BMSCs & PDLSCs; Sheep; 3; NA | Biphasic scaffold | 5 & 10; NB, NC, NPDL |
| Wei 2012 (48) | Swine; Minipigs; 12 months/40-50 kg | Experimental periodontal defects; 5×7×3 | Allogeneic | PDLSCs; Swine; NA; NA | Gelfoam | 12; NB |
| Weng 2006 (49) | Canine; Mongrel dogs; 12-24 months/15 kg | Supraalveolar bone defects; 5 | Autologous | BMSCs; Canine; 3; 5×10^7^ | Calcium alginate | 12; NB |
| Xu 2007 (50) | Canine; Mongrel dogs; Male; 1 year/11-15 kg | Horizontal alveolar bone defects; 5×2 | Autologous | BMSCs; Canine; 3~5; 2×10^8^ | AP-C/PLA or C/PLA | 4 & 8; NB, NC |
| Yan 2015 (51) | Rodent; Nude rats (Hsd: RH-Foxn1rnu); Male; 6 weeks | Intra-bony three-wall periodontal defects; 2×2×1.7 | Allogeneic | PDLSCs; Rat; 5; 2.4×10^5^ | Chitosan hydrogel | 4; NB, NPDL |
| Yang 2010 (52) | Rodent; SD rats; Male; 8 weeks | Periodontal fenestration defects; 1×3 | Allogeneic | BMSCs; Rat; Day 25; NA | Gelatin beads | 3; NB, NC, NPDL |
| Yoo 2019 (53) | Canine; Beagle dogs; Male; 15-20 months/10 kg | Periodontal dehiscence defects; 5 | Allogeneic | PDLSCs; Canine; 3~4; 1×10^6^ | Collagen matrix | 8; NB, NC |
| Yu 2016 (54) | Rodent; SD rats; Male; 220 ± 20 g | Periodontal defects; 3×1.5 | Xenogeneic | PDLSCs; Human; 4; NA | CBB | 4; NB, NC, NPDL |
| Yu 2018 (55) | Rodent; Wistar rats; Male; 6 weeks/260-300 g | Periodontal bone defects; 5x2x1 | Allogeneic | BMSCs; Rat; 3~5; 1×10^4^ | Type I collagen membranes | 2 & 4 & 6; NB |
| Yu N 2013 (56) | Rodent; Nude rats (Crl:NIH Foxn1rnu); 7 weeks | 3-wall intra-bony periodontal defects; 2×2×1.7 | Allogeneic | PDLSCs & GMSCs; Rat; 4; NA | Gelatin sponges | 6; NB, NPDL |
| Yu XB 2013 (57) | Canine; Beagle dogs; Male; Adult /10-11 kg | Class III furcation defects; 5; (Cotton balls saturated with anaerobic bacteria; 4 weeks) | Xenogeneic | GMSCs; Human; NA; NA | NA | 8; NB, NC |
| Zang 2016 (58) | Canine; Beagle dogs; Male; 15 months/10-15 kg | 1-wall infra-bony defects; 7×4 | Xenogeneic | BMSCs; Human; 3~5; 1×10^7^ | C or C/ABB | 8; NB |
| Zhan 2008 (59) | Canine; Beagle dogs; 18 months | Class II furcation defects; 5×3 | Allogeneic | BMSCs; Canine; 1; NA | Bio-Gide membrane | 12; NB, NC |
| Zhou 2012 (60) | Canine; Beagle dogs; Male; Adult/10-14 kg | Periodontal fenestration defects; 4×4×3 | Allogeneic | BMSCs; Canine; NA; NA | PLGA | 6; NB, NC |

**Abbreviations:** ABB, Anorganic bovine bone; ADSCs, Adipose tissue-derived stem cells; AM, amniotic membrane; ASA, acetylsalicylic acid; C, chitosan; CaP-PCL, Calcium phosphate coated polycaprolactone; CBB, calcined bovine bone; C-MSC, MSC/extracellular matrix (ECM) complex (C-MSC); DBCB, deprotei-nized bovine cancellous bone; DPSCs, Dental Pulp Stem Cells; e-PTFE, expanded polytetrafluoroethylene; GMSCs, gingival-derived stem cells; HA/TCP, hydroxyapatite/tricalcium phosphate; LPS, Lipopolysaccharide; NB, newly formed bone; NC, newly formed cementum; nHAC, nano-Hap-collagen; NA, Not mentioned; NPDL, newly formed periodontal ligament; NS, Normal saline; OIM, osteoinductive medium; OPG, Osteoprotegerin; PBS, phosphate-buffered saline; PCL, poly(ɛ-caprolactone) electrospun; PDLSCs, periodontal ligament stem cells; PF127, Pluronic F127; Pg, Porphyromonas gingivalis; PGA, polyglycolic acid; PHD2, Prolyl hydroxylase domain-containing protein 2; PisPLLA/COL/HA matrix, PisPLLA and collagen (1:1) and 30 wt% hydroxyapatite ; PL, platelet lysate; PLAP-1, periodontal ligament-associated protein-1; PLGA, Poly d,l-lactic-co-glycolic acid; PLLA/COL/HA, PLLA and collagen (1:1) and 30 wt% hydroxyapatite; PRF, platelet-rich fibrin; PRP, platelet-rich plasma; RSV, Resveratrol; SD rats, Sprague Dawley rats; TDMP, treated dentin matrix particles.

**Reference**

1. Akita D, Morokuma M, Saito Y, Yamanaka K, Akiyama Y, Sato M, et al. Periodontal tissue regeneration by transplantation of rat adipose-derived stromal cells in combination with PLGA-based solid scaffolds. Biomedical Research (Japan). 2014;35(2):91-103.

2. Akita D, Kano K, Saito-Tamura Y, Mashimo T, Sato-Shionome M, Tsurumachi N, et al. Use of rat mature adipocyte-derived dedifferentiated fat cells as a cell source for periodontal tissue regeneration. Frontiers in Physiology. 2016;7(FEB).

3. Akizuki T, Oda S, Komaki M, Tsuchioka H, Kawakatsu N, Kikuchi A, et al. Application of periodontal ligament cell sheet for periodontal regeneration: a pilot study in beagle dogs. J Periodontal Res. 2005;40(3):245-51.

4. Babo PS, Cai X, Plachokova AS, Reis RL, Jansen JA, Gomes ME, et al. The Role of a Platelet Lysate-Based Compartmentalized System as a Carrier of Cells and Platelet-Origin Cytokines for Periodontal Tissue Regeneration. Tissue Eng Part A. 2016;22(19-20):1164-75.

5. Cai X, Yang F, Yan X, Yang W, Yu N, Oortgiesen DAW, et al. Influence of bone marrow-derived mesenchymal stem cells pre-implantation differentiation approach on periodontal regeneration in vivo. Journal of clinical periodontology. 2015;42(4):380-9.

6. Cao Y, Liu Z, Xie Y, Hu J, Wang H, Fan Z, et al. Adenovirus-mediated transfer of hepatocyte growth factor gene to human dental pulp stem cells under good manufacturing practice improves their potential for periodontal regeneration in swine. Stem Cell Res Ther. 2015;6:249-.

7. Dan H, Vaquette C, Fisher AG, Hamlet SM, Xiao Y, Hutmacher DW, et al. The influence of cellular source on periodontal regeneration using calcium phosphate coated polycaprolactone scaffold supported cell sheets. Biomaterials. 2014;35(1):113-22.

8. Ding G, Liu Y, Wang W, Wei F, Liu D, Fan Z, et al. Allogeneic periodontal ligament stem cell therapy for periodontitis in swine. Stem cells (Dayton, Ohio). 2010;28(10):1829-38.

9. Dogan A, Ozdemir A, Kubar A, Oygür T. Assessment of periodontal healing by seeding of fibroblast-like cells derived from regenerated periodontal ligament in artificial furcation defects in a dog: a pilot study. Tissue engineering. 2002;8(2):273-82.

10. Doǧan A, Özdemir A, Kubar A, Oygür T. Healing of Artificial Fenestration Defects by Seeding of Fibroblast-Like Cells Derived from Regenerated Periodontal Ligament in a Dog: A Preliminary Study. Tissue Engineering. 2003;9(6):1189-96.

11. Du J, Shan Z, Ma P, Wang S, Fan Z. Allogeneic bone marrow mesenchymal stem cell transplantation for periodontal regeneration. J Dent Res. 2014;93(2):183-8.

12. Duan X, Lin Z, Lin X, Wang Z, Wu Y, Ji M, et al. Study of platelet-rich fibrin combined with rat periodontal ligament stem cells in periodontal tissue regeneration. Journal of Cellular and Molecular Medicine. 2018;22(2):1047-55.

13. Fawzy El-Sayed KM, Mekhemar MK, Beck-Broichsitter BE, Bähr T, Hegab M, Receveur J, et al. Periodontal regeneration employing gingival margin-derived stem/progenitor cells in conjunction with IL-1ra-hydrogel synthetic extracellular matrix. Journal of clinical periodontology. 2015;42(5):448-57.

14. Fu X, Jin L, Ma P, Fan Z, Wang S. Allogeneic stem cells from deciduous teeth in treatment for periodontitis in miniature swine. Journal of periodontology. 2014;85(6):845-51.

15. Guo S, Kang J, Ji B, Guo W, Ding Y, Wu Y, et al. Periodontal-Derived Mesenchymal Cell Sheets Promote Periodontal Regeneration in Inflammatory Microenvironment. Tissue Eng Part A. 2017;23(13-14):585-96.

16. Han J, Menicanin D, Marino V, Ge S, Mrozik K, Gronthos S, et al. Assessment of the regenerative potential of allogeneic periodontal ligament stem cells in a rodent periodontal defect model. J Periodontal Res. 2014;49(3):333-45.

17. Hu J, Cao Y, Xie Y, Wang H, Fan Z, Wang J, et al. Periodontal regeneration in swine after cell injection and cell sheet transplantation of human dental pulp stem cells following good manufacturing practice. Stem Cell Res Ther. 2016;7(1):130-.

18. Iwata T, Yamato M, Tsuchioka H, Takagi R, Mukobata S, Washio K, et al. Periodontal regeneration with multi-layered periodontal ligament-derived cell sheets in a canine model. Biomaterials. 2009;30(14):2716-23.

19. Jiang S, Tang K, Chen B, Yan F. Regenerative effect of hOPG gene-modified autologous PDLs in combination with cell transplantation on periodontal defection in beagle dogs. Cytotechnology. 2016;68(6):2613-23.

20. Khorsand A, Eslaminejad MB, Arabsolghar M, Paknejad M, Ghaedi B, Rokn AR, et al. Autologous dental pulp stem cells in regeneration of defect created in canine periodontal tissue. J Oral Implantol. 2013;39(4):433-43.

21. Lemaitre M, Monsarrat P, Blasco-Baque V, Loubiêres P, Burcelin R, Casteilla L, et al. Periodontal tissue regeneration using syngeneic adipose-derived stromal cells in a mouse model. Stem Cells Translational Medicine. 2017;6(2):656-65.

22. Li H, Yan F, Lei L, Li Y, Xiao Y. Application of autologous cryopreserved bone marrow mesenchymal stem cells for periodontal regeneration in dogs. Cells Tissues Organs. 2009;190(2):94-101.

23. Li Y-F, Yan F-H, Zhong Q, Zhao X. Effect of hBMP-7 gene modified bone marrow stromal cells on periodontal tissue regeneration. Zhonghua Yi Xue Za Zhi. 2010;90(20):1427-30.

24. Li G, Han N, Zhang X, Yang H, Cao Y, Wang S, et al. Local Injection of Allogeneic Stem Cells from Apical Papilla Enhanced Periodontal Tissue Regeneration in Minipig Model of Periodontitis. BioMed Research International. 2018;2018.

25. Liu Y, Zheng Y, Ding G, Fang D, Zhang C, Bartold PM, et al. Periodontal ligament stem cell-mediated treatment for periodontitis in miniature swine. Stem Cells. 2008;26(4):1065-73.

26. Liu D, Wang Y, Jia Z, Wang L, Wang J, Yang D, et al. Demethylation of IGFBP5 by Histone Demethylase KDM6B Promotes Mesenchymal Stem Cell-Mediated Periodontal Tissue Regeneration by Enhancing Osteogenic Differentiation and Anti-Inflammation Potentials. Stem Cells. 2015;33(8):2523-36.

27. Lu H, Wu Z-F, Tian Y. A study on the effects of cells and scaffolds tissue engineering on the periodontal regeneration. Zhonghua Kou Qiang Yi Xue Za Zhi. 2004;39(3):189-92.

28. Ma L, Hu J, Cao Y, Xie Y, Wang H, Fan Z, et al. Maintained Properties of Aged Dental Pulp Stem Cells for Superior Periodontal Tissue Regeneration. Aging and disease. 2019;10(4):793-806.

29. Mohammed E, Khalil E, Sabry D. Effect of adipose-derived stem cells and their exo as adjunctive therapy to nonsurgical periodontal treatment: A histologic and histomorphometric study in rats. Biomolecules. 2018;8(4).

30. Mrozik KM, Wada N, Marino V, Richter W, Shi S, Wheeler DL, et al. Regeneration of periodontal tissues using allogeneic periodontal ligament stem cells in an ovine model. Regenerative Medicine. 2013;8(6):711-23.

31. Nagahara T, Yoshimatsu S, Shiba H, Kawaguchi H, Takeda K, Iwata T, et al. Introduction of a mixture of β-tricalcium phosphate into a complex of bone marrow mesenchymal stem cells and type I collagen can augment the volume of alveolar bone without impairing cementum regeneration. Journal of periodontology. 2015;86(3):456-64.

32. Nakahara T, Nakamura T, Kobayashi E, Kuremoto K-I, Matsuno T, Tabata Y, et al. In situ tissue engineering of periodontal tissues by seeding with periodontal ligament-derived cells. Tissue engineering. 2004;10(3-4):537-44.

33. Nuñez J, Sanz-Blasco S, Vignoletti F, Muñoz F, Arzate H, Villalobos C, et al. Periodontal regeneration following implantation of cementum and periodontal ligament-derived cells. J Periodontal Res. 2012;47(1):33-44.

34. Ozasa M, Sawada K, Iwayama T, Yamamoto S, Morimoto C, Okura H, et al. Periodontal tissue regeneration by transplantation of adipose tissue-derived multi-lineage progenitor cells. Inflammation and Regeneration. 2014;34(2):109-16.

35. Paknejad M, Eslaminejad MB, Ghaedi B, Rokn A-R, Khorsand A, Etemad-Moghadam S, et al. Isolation and Assessment of Mesenchymal Stem Cells Derived From Bone Marrow: Histologic and Histomorphometric Study in a Canine Periodontal Defect. J Oral Implantol. 2015;41(3):284-91.

36. Park J-Y, Jeon SH, Choung P-H. Efficacy of periodontal stem cell transplantation in the treatment of advanced periodontitis. Cell transplantation. 2011;20(2):271-85.

37. Rezaei M, Jamshidi S, Saffarpour A, Ashouri M, Rahbarghazi R, Rokn AR, et al. Transplantation of Bone Marrow-Derived Mesenchymal Stem Cells, Platelet-Rich Plasma, and Fibrin Glue for Periodontal Regeneration. Int J Periodontics Restorative Dent. 2019;39(1):e32-e45.

38. Sano K, Usui M, Moritani Y, Nakazawa K, Hanatani T, Kondo H, et al. Co-cultured spheroids of human periodontal ligament mesenchymal stem cells and vascular endothelial cells enhance periodontal tissue regeneration. Regenerative Therapy. 2020;14:59-71.

39. Simsek SB, Keles GC, Baris S, Cetinkaya BO. Comparison of mesenchymal stem cells and autogenous cortical bone graft in the treatment of class II furcation defects in dogs. Clin Oral Investig. 2012;16(1):251-8.

40. Suaid FF, Ribeiro FV, Rodrigues TL, Silvério KG, Carvalho MD, Nociti FH, Jr., et al. Autologous periodontal ligament cells in the treatment of class II furcation defects: a study in dogs. Journal of clinical periodontology. 2011;38(5):491-8.

41. Suaid FF, Ribeiro FV, Gomes TRLES, Silvério KG, Carvalho MD, Nociti FH, Jr., et al. Autologous periodontal ligament cells in the treatment of Class III furcation defects: a study in dogs. Journal of clinical periodontology. 2012;39(4):377-84.

42. Takewaki M, Kajiya M, Takeda K, Sasaki S, Motoike S, Komatsu N, et al. MSC/ECM Cellular Complexes Induce Periodontal Tissue Regeneration. J Dent Res. 2017;96(9):984-91.

43. Tcacencu I, Karlström E, Cedervall J, Wendel M. Transplanted Human Bone Marrow Mesenchymal Stem Cells Seeded onto Peptide Hydrogel Decrease Alveolar Bone Loss. BioResearch open access. 2012;1(5):215-21.

44. Tobita M, Uysal CA, Guo X, Hyakusoku H, Mizuno H. Periodontal tissue regeneration by combined implantation of adipose tissue-derived stem cells and platelet-rich plasma in a canine model. Cytotherapy. 2013;15(12):1517-26.

45. Tsumanuma Y, Iwata T, Washio K, Yoshida T, Yamada A, Takagi R, et al. Comparison of different tissue-derived stem cell sheets for periodontal regeneration in a canine 1-wall defect model. Biomaterials. 2011;32(25):5819-25.

46. Tsumanuma Y, Iwata T, Kinoshita A, Washio K, Yoshida T, Yamada A, et al. Allogeneic Transplantation of Periodontal Ligament-Derived Multipotent Mesenchymal Stromal Cell Sheets in Canine Critical-Size Supra-Alveolar Periodontal Defect Model. BioResearch Open Access. 2016;5(1):22-36.

47. Vaquette C, Saifzadeh S, Farag A, Hutmacher DW, Ivanovski S. Periodontal Tissue Engineering with a Multiphasic Construct and Cell Sheets. J Dent Res. 2019;98(6):673-81.

48. Wei F, Qu C, Song T, Ding G, Fan Z, Liu D, et al. Vitamin C treatment promotes mesenchymal stem cell sheet formation and tissue regeneration by elevating telomerase activity. Journal of Cellular Physiology. 2012;227(9):3216-24.

49. Weng Y, Wang M, Liu W, Hu X, Chai G, Yan Q, et al. Repair of experimental alveolar bone defects by tissue-engineered bone. Tissue engineering. 2006;12(6):1503-13.

50. Xu C, Xian X, Guo F. An experimental study on effect of astragalus polysaccharides on chitosan/polylactic acid scaffolds for repairing alveolar bone defects in dogs. Zhongguo Xiu Fu Chong Jian Wai Ke Za Zhi. 2007;21(7):748-52.

51. Yan XZ, Van Den Beucken JJJP, Cai X, Yu N, Jansen JA, Yang F. Periodontal tissue regeneration using enzymatically solidified chitosan hydrogels with or without cell loading. Tissue Engineering - Part A. 2015;21(5-6):1066-76.

52. Yang Y, Rossi FMV, Putnins EE. Periodontal regeneration using engineered bone marrow mesenchymal stromal cells. Biomaterials. 2010;31(33):8574-82.

53. Yoo S-Y, Lee J-S, Cha J-K, Kim S-K, Kim C-S. Periodontal healing using a collagen matrix with periodontal ligament progenitor cells in a dehiscence defect model in beagle dogs. J Periodontal Implant Sci. 2019;49(4):215-27.

54. Yu Y, Bi CS, Wu RX, Yin Y, Zhang XY, Lan PH, et al. Effects of short-term inflammatory and/or hypoxic pretreatments on periodontal ligament stem cells: in vitro and in vivo studies. Cell and Tissue Research. 2016;366(2):311-28.

55. Yu X, Liu S, Wang W, Li S. Periodontal ligament‑associated protein‑1 delays rat periodontal bone defect repair by regulating osteogenic differentiation of bone marrow stromal cells and osteoclast activation. International journal of molecular medicine. 2018;41(2):1110-8.

56. Yu N, Oortgiesen DAW, Bronckers ALJJ, Yang F, Walboomers XF, Jansen JA. Enhanced periodontal tissue regeneration by periodontal cell implantation. Journal of clinical periodontology. 2013;40(7):698-706.

57. Yu X, Ge S, Chen S, Xu Q, Zhang J, Guo H, et al. Human gingiva-derived mesenchymal stromal cells contribute to periodontal regeneration in beagle dogs. Cells Tissues Organs. 2013;198(6):428-37.

58. Zang S, Jin L, Kang S, Hu X, Wang M, Wang J, et al. Periodontal Wound Healing by Transplantation of Jaw Bone Marrow-Derived Mesenchymal Stem Cells in Chitosan/Anorganic Bovine Bone Carrier Into One-Wall Infrabony Defects in Beagles. Journal of periodontology. 2016;87(8):971-81.

59. Zhan X, Yan FH, Xiao Y. Different densities of autologous bone marrow mesenchymal stem cell transplantation for repairing Beagle canine teeth class II furcation defects. Journal of Clinical Rehabilitative Tissue Engineering Research. 2008;12(16):3193-7.

60. Zhou W, Mei L. Effect of autologous bone marrow stromal cells transduced with osteoprotegerin on periodontal bone regeneration in canine periodontal window defects. Int J Periodontics Restorative Dent. 2012;32(5):e174-e81.
