# Supplementary material for: Stem cell therapies for periodontal tissue regeneration: a network meta-analysis of preclinical studies
Source: Stem Cell Res Ther. 2020 Oct 2;11:427. doi: 10.1186/s13287-020-01938-7 (PMC7531120; doi:10.1186/s13287-020-01938-7)
Supplement: Supplementary file 5 — Additional file 5. : Supplementary Table 5. Summary of the characteristics of included studies. [file 13287_2020_1938_MOESM5_ESM.docx]

**Supplementary Table 5. Summary of the characteristics of included studies.**

| Index | Item | Count | Percentage |
| --- | --- | --- | --- |
| *Study arms* | Two | 53 | 88.33% |
|  | Three | 6 | 10.00% |
|  | Four | 1 | 1.67% |
| *Animal species* | Canine | 31 | 51.67% |
|  | Rodent | 17 | 28.33% |
|  | Swine | 10 | 16.67% |
|  | Ovine | 2 | 3.33% |
| *Periodontal defects* | Intrabony defects | 13 | 21.67% |
|  | Furcation defects | 16 | 26.67% |
|  | Fenestration defects | 10 | 16.67% |
|  | Periodontal bone defects | 5 | 8.33% |
|  | Periodontitis defects | 7 | 11.67% |
|  | Dehiscence defects | 4 | 6.67% |
|  | Other (Unclear) | 5 | 8.33% |
| *Types of stem cells* | PDLSCs | 30 | 50.00% |
|  | BMSCs | 20 | 33.33% |
|  | ADSCs | 6 | 10.00% |
|  | DPSCs | 6 | 10.00% |
|  | GMSCs | 5 | 8.33% |
| *Transplant type* | Autologous | 28 | 46.67% |
|  | Allogeneic | 23 | 38.33% |
|  | Xenogeneic | 12 | 20.00% |
| *Outcomes* | NB | 55 | 91.67% |
|  | NC | 41 | 68.33% |
|  | NPDL | 17 | 28.33% |

**Abbreviations:** ADSCs, adipose tissue-derived stem cells; BMSCs, bone marrow-derived stem cells; DPSCs, dental pulp stem cells; GMSCs, gingival-derived stem cells; NB, newly formed bone; NC, newly formed cementum; NPDL, newly formed periodontal ligament; PDLSCs, periodontal ligament stem cells.
