# Supplementary material for: Stem cell therapies for periodontal tissue regeneration: a network meta-analysis of preclinical studies
Source: Stem Cell Res Ther. 2020 Oct 2;11:427. doi: 10.1186/s13287-020-01938-7 (PMC7531120; doi:10.1186/s13287-020-01938-7)
Supplement: Supplementary file 6 — Additional file 6. : Supplementary Fig. 1. Risk of bias of included preclinical studies. Review author’s judgments about each risk of bias item for each included study. +, low risk; −, high risk;?, unclear risk. [file 13287_2020_1938_MOESM6_ESM.pdf]

Supplementary Figure 1

	Sequence generation	Baseline characteristics	Allocation concealment	Random housing	Blinding against performance bias	Random outcome assessment	Blinding against detection bias	Incomplete outcome data	Selective outcome reporting	Other sources of biases
Akita 2014	?	+	?	?	?	?	?	+	?	?
Akita 2016	?	+	?	?	?	?	?	?	?	?
Akizuki 2005	−	+	?	?	?	?	+	+	+	?
Babo 2016	?	+	?	?	?	?	?	+	+	?
Cai 2015	+	+	?	?	?	?	?	+	+	?
Cao 2015	?	+	?	?	?	?	+	+	+	?
Dan 2014	?	?	?	?	?	?	?	+	?	?
Ding 2010	?	+	?	?	?	?	?	+	+	?
Doğan 2002	−	+	?	?	?	?	?	+	?	−
Doğan 2003	−	+	?	?	?	?	?	+	?	−
Du 2014	+	+	?	?	?	?	?	+	?	?
Duan 2011	?	+	?	?	?	?	?	+	+	?
Fawzy El-Sayed 2015	+	+	+	?	+	?	+	+	+	?
Fu 2014	+	+	?	?	?	?	?	+	?	?
Guo 2017	?	+	?	?	?	?	?	?	?	?
Han 2014	?	+	?	?	?	?	+	?	?	−
Hu 2016	?	+	?	?	?	?	?	+	?	?
Iwata 2009	?	+	?	?	?	?	+	+	+	?
Jiang 2016	?	+	?	?	?	?	+	+	+	?
Khorsand 2013	?	+	?	?	?	?	?	?	+	−
Lemaitre 2016	?	+	?	?	?	?	?	?	?	?
Li 2009	+	+	?	?	+	?	+	+	?	−
Li 2010	?	+	?	?	?	?	?	+	+	?
Li 2018	?	+	?	?	?	?	?	+	+	?
Liu 2008	?	+	?	?	?	?	?	+	?	?
Liu 2015	?	+	?	?	?	?	?	+	?	?
Lu 2004	+	?	?	?	?	?	?	+	?	?
Ma 2019	?	+	?	?	?	?	?	?	?	?
Mohammed 2018	?	+	?	+	?	?	?	+	?	?
Mrozik 2013	−	+	?	?	?	?	+	+	+	−
Nagahara 2015	?	+	?	?	?	?	?	+	?	?
Nakahara 2004	?	+	?	?	?	?	+	+	+	−
Nuñez 2012	+	+	+	?	+	?	+	+	+	?
Ozasa 2014	−	+	?	?	?	?	?	+	?	?
Paknejad 2015	?	+	?	?	?	?	?	+	?	?
Park 2011	?	+	?	?	?	?	+	?	?	?
Rezaei 2019	?	+	?	?	?	?	+	+	+	?
Sano 2020	?	+	?	?	?	?	?	+	+	?
Simsek 2012	−	+	?	?	?	?	+	+	?	?
Suaid 2011	+	+	+	?	+	?	+	+	+	?
Suaid 2012	+	+	+	?	+	?	+	+	+	?
Takewaki 2017	?	+	?	?	?	?	?	+	?	?
Tcacencu 2012	?	+	?	?	?	?	?	?	?	−
Tobita 2013	?	+	?	?	?	?	?	?	?	?
Tsumanuma 2011	?	+	?	?	?	?	?	+	?	?
Tsumanuma 2016	−	+	?	?	?	?	+	+	+	+
Vaquette 2019	?	?	?	?	?	?	?	+	+	+
Wei 2012	?	?	?	?	?	?	?	+	?	?
Weng 2006	?	+	?	?	?	?	?	+	+	?
Xu 2006	?	?	?	?	?	+	+	?	?	?
Yan 2015	?	+	?	?	?	?	+	+	+	+
Yang 2010	?	+	?	?	?	?	?	+	?	?
Yoo 2019	?	+	?	?	?	?	?	+	+	+
Yu 2016	?	+	?	?	?	?	?	?	+	?
Yu 2018	?	?	?	?	?	?	?	?	?	?
Yu N 2013	+	+	?	?	?	?	?	+	?	?
Yu XB 2013	−	+	?	?	?	?	?	+	+	?
Zang 2016	+	+	?	?	?	?	+	+	+	?
Zhan 2008	?	+	?	?	?	?	?	+	+	?
Zhou 2012	?	+	?	?	?	?	?	+	?	?

Supplementary Figure 1. Risk of bias of included preclinical studies (Review authors’ judgments about each risk of bias item for each included study. +, low risk; −, high risk; ?, unclear risk).
