# Supplementary material for: Stem cell therapies for periodontal tissue regeneration: a network meta-analysis of preclinical studies
Source: Stem Cell Res Ther. 2020 Oct 2;11:427. doi: 10.1186/s13287-020-01938-7 (PMC7531120; doi:10.1186/s13287-020-01938-7)
Supplement: Supplementary file 7 — Additional file 7. : Supplementary Fig. 2. Forest plots of pairwise meta-analyses showing the SMD and 95% CI of alveolar bone, cementum, and periodontal ligament regeneration for each included study. The graphs were generated using the ‘mvmeta’ suite in Stata. For all the plots, the solid vertical line (0) indicates no effect, SMDs of more than 0 favor stem cells on the right side of the x-axis. The size of the box indicates the weighting of each study, and the thin horizontal whisker indicates the 95% CI. The diamond represents overall effect size. Random-effects model was used to summarize the effect sizes. Heterogeneity is denoted by the Ι2. [file 13287_2020_1938_MOESM7_ESM.docx]

**Supplementary Figure 2 Forest plots of pairwise meta-analyses** **showing the SMD and 95% CI of alveolar bone, cementum, and periodontal ligament regeneration for each included study.** The graphs were generated using the ‘mvmeta’ suite in Stata. For all the plots, the solid vertical line (0) indicates no effect, SMDs of more than 0 favor stem cells on the right side of the x-axis. The size of the box indicates the weighting of each study, and the thin horizontal whisker indicates the 95% CI. The diamond represents the overall effect size. The random-effects model was used to summarise the effect sizes. The Ι^2^ denotes heterogeneity. **Abbreviations:** ADSCs, adipose tissue-derived stem cells; BMSCs, bone marrow-derived stem cells; CC, cell carrier; CI, confidence interval; DPSCs, dental pulp stem cells; GMSCs, gingival-derived stem cells; NB, newly formed bone; NC, newly formed cementum; NPDL, newly formed periodontal ligament; PDLSCs, periodontal ligament stem cells; SMD, standardized mean difference.

**Outcome 1 NB**

**
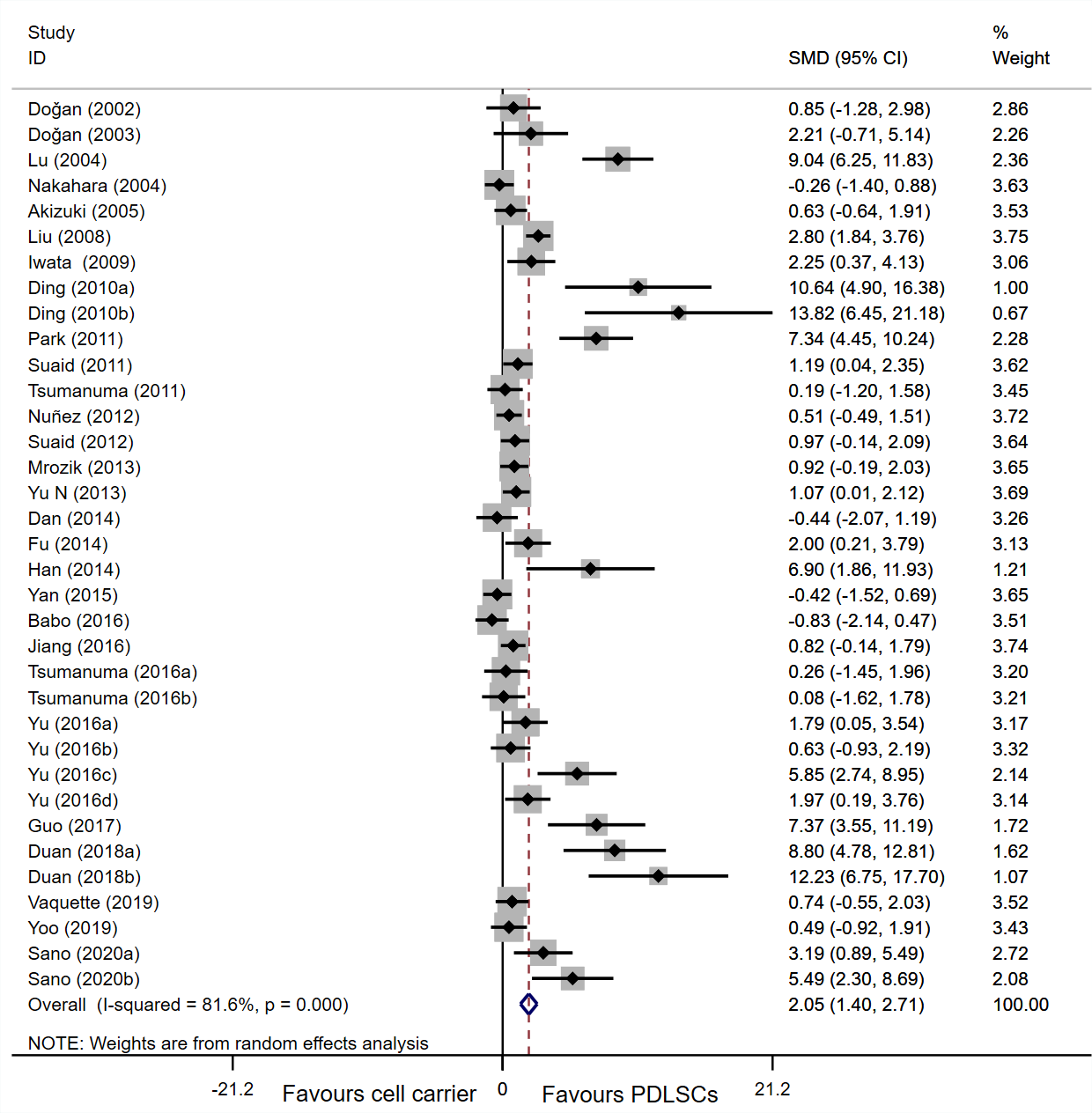
**

**Analysis 1.1 Comparison 1 PDLSCs versus cell carrier, Outcome 1 NB.**

**
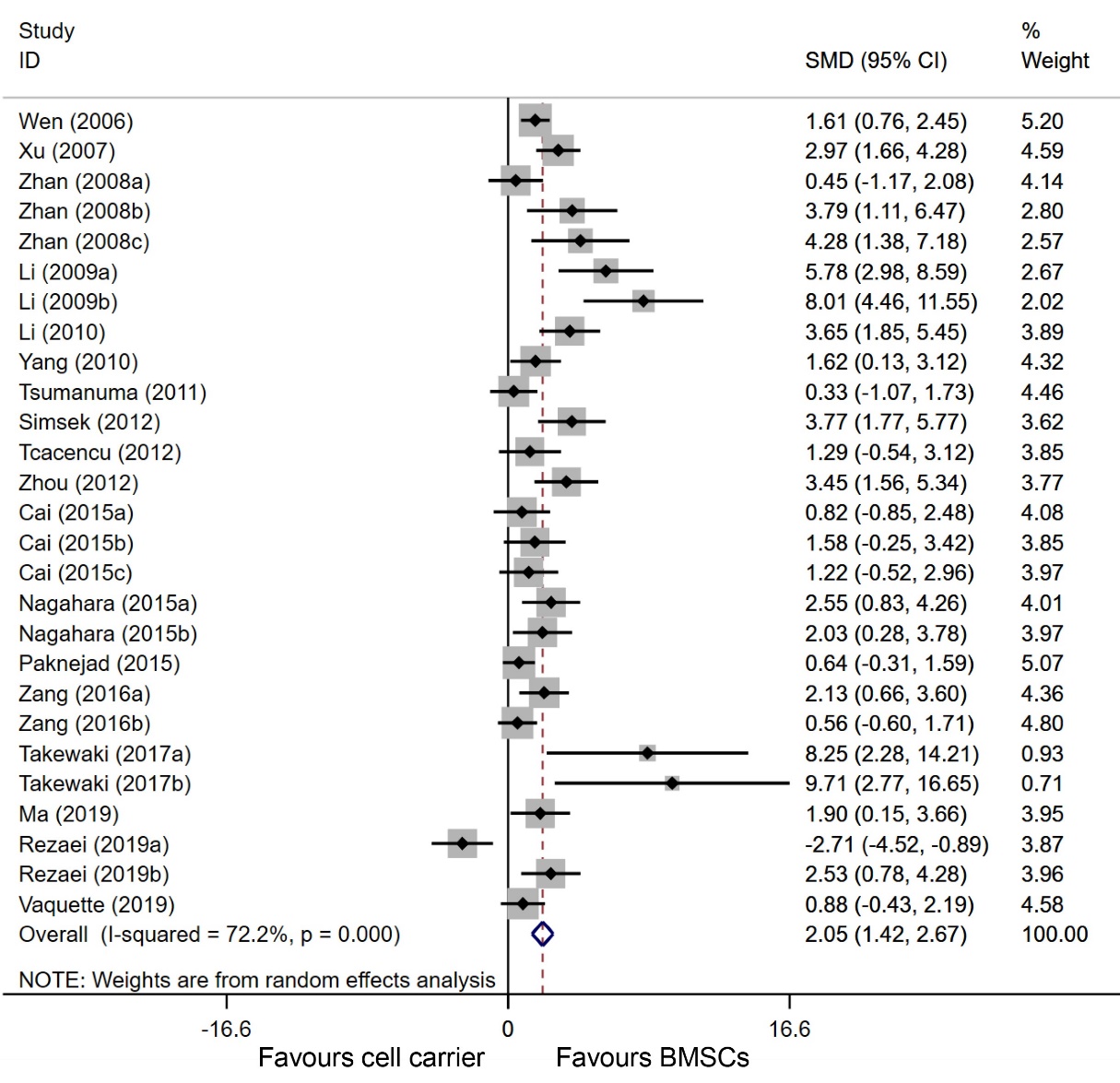
**

**Analysis 1.2 Comparison 2 BMSCs versus cell carrier, Outcome 1 NB.**

**
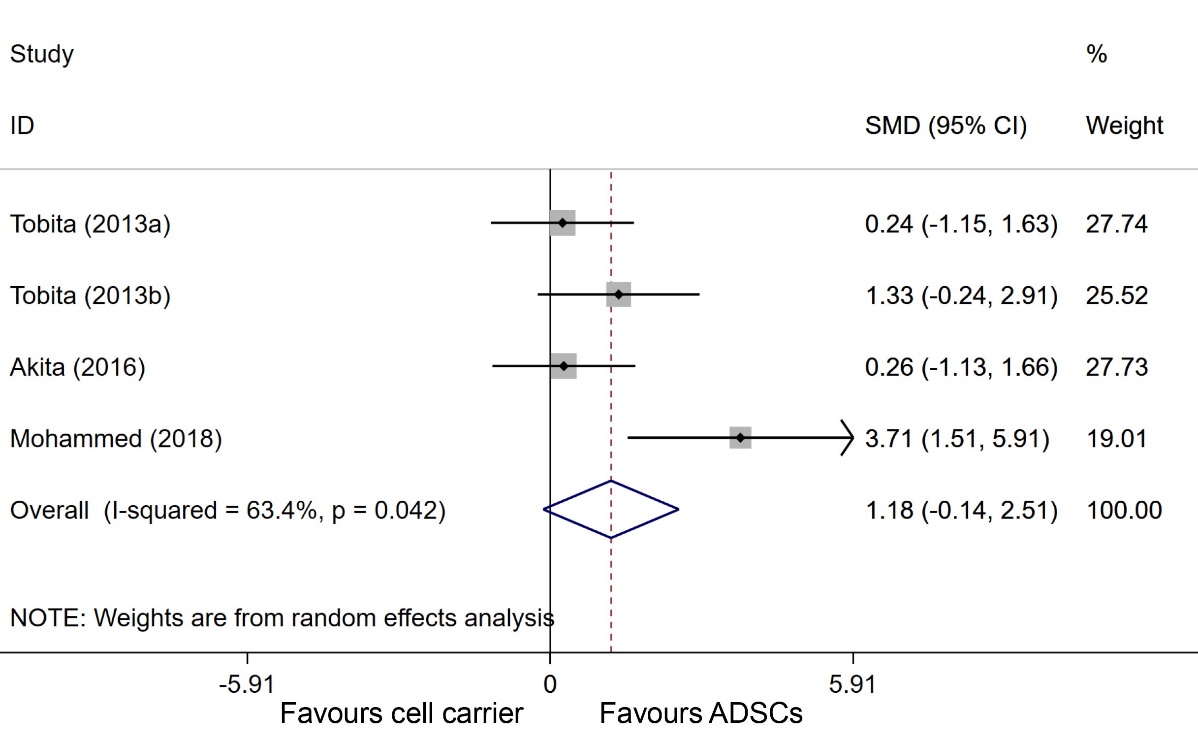
**

**Analysis 1.3 Comparison 3 ADSCs versus cell carrier, Outcome 1 NB.**

**
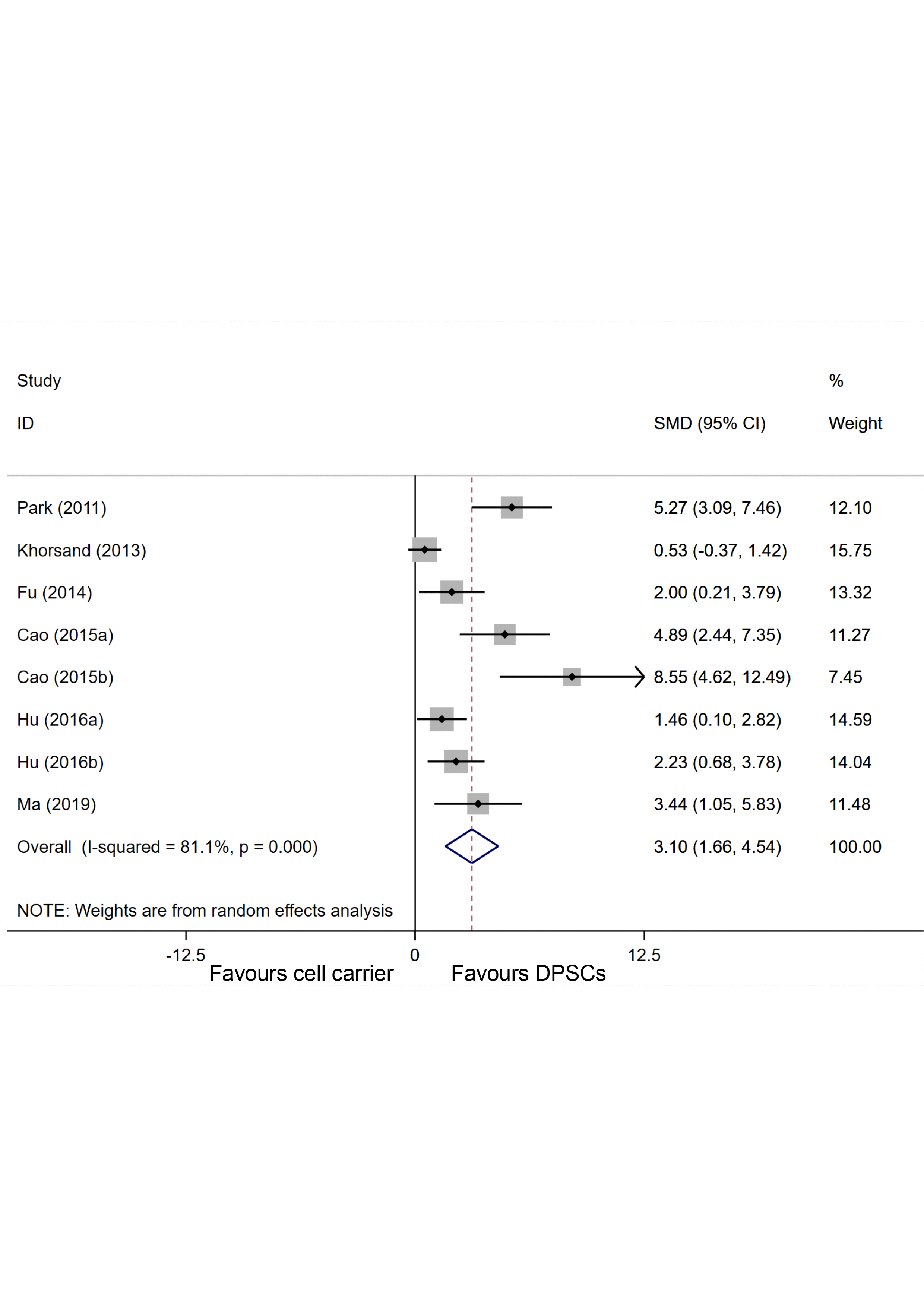
**

**Analysis 1.4 Comparison 4 DPSCs versus cell carrier, Outcome 1 NB.**

**
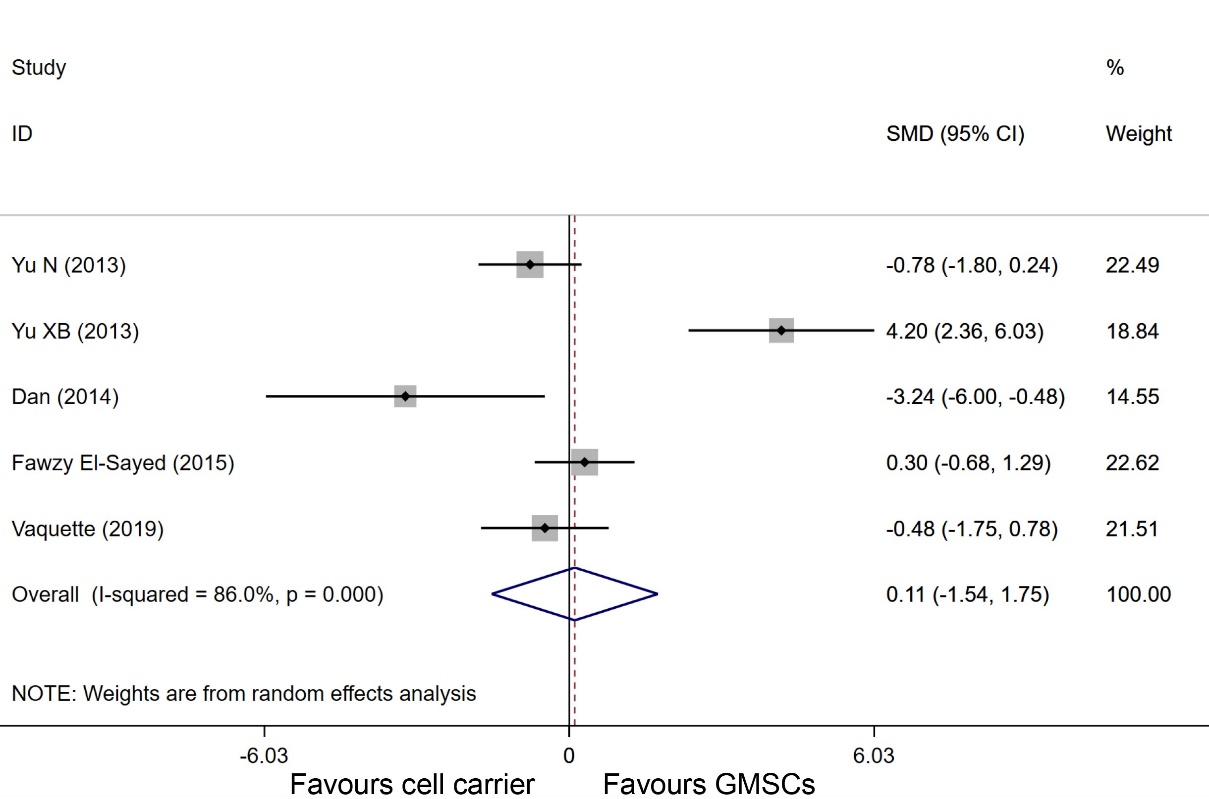
**

**Analysis 1.5 Comparison 5 GMSCs versus cell carrier, Outcome 1 NB.**

**
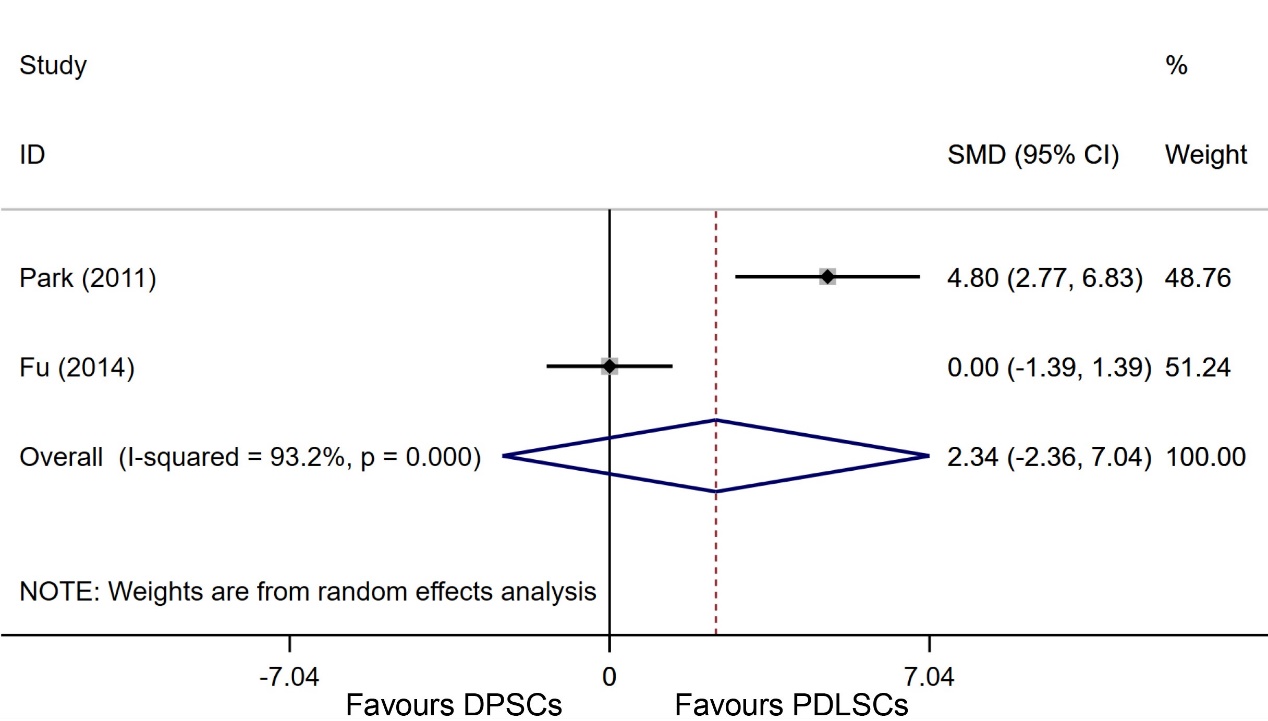
**

**Analysis 1.6 Comparison 6 PDLSCs versus DPSCs, Outcome 1 NB.**

**
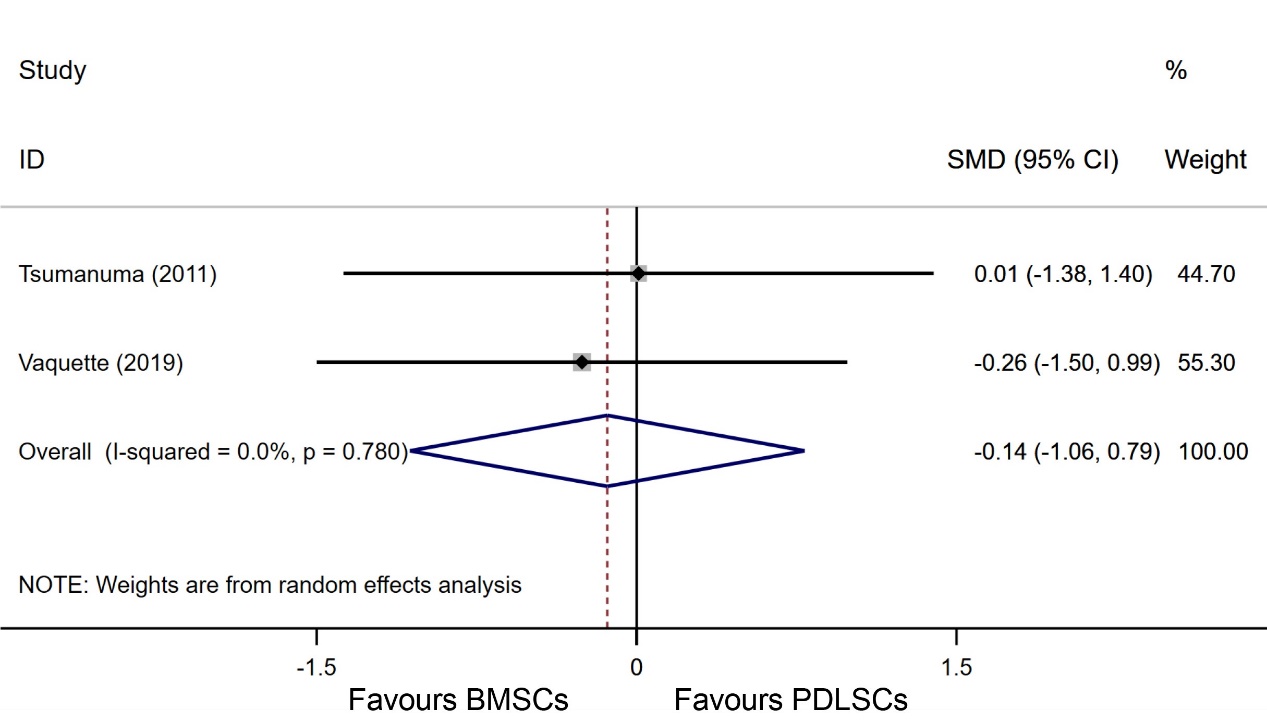
**

**Analysis 1.7 Comparison 7 PDLSCs versus BMSCs, Outcome 1 NB.**

**
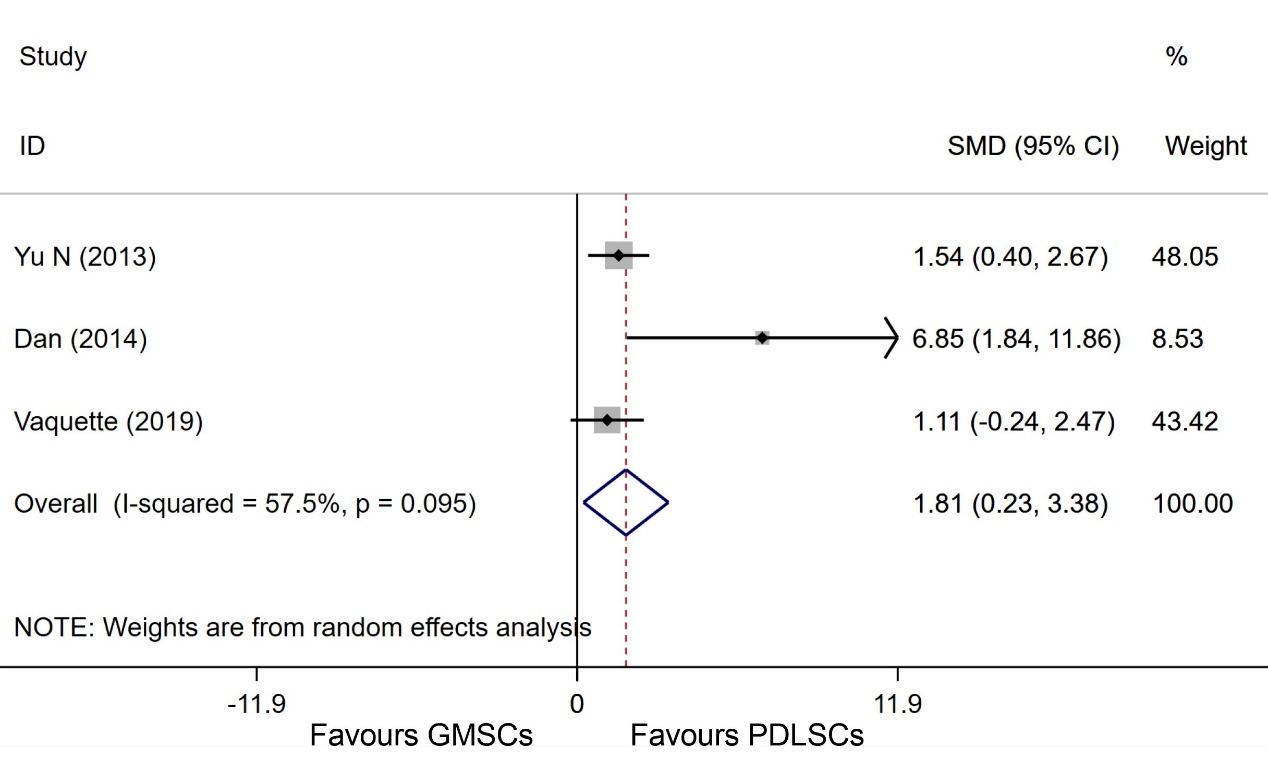
**

**Analysis 1.8 Comparison 8 PDLSCs versus GMSCs, Outcome 1 NB.**

**
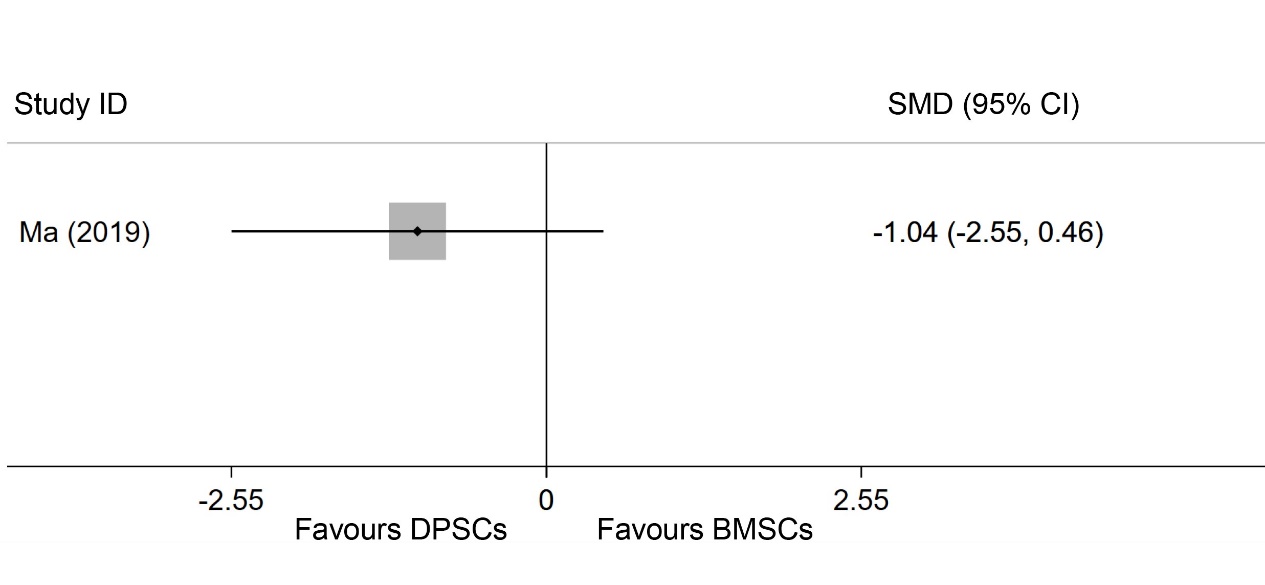
**

**Analysis 1.9 Comparison 9 BMSCs versus DPSCs, Outcome 1 NB.**

**
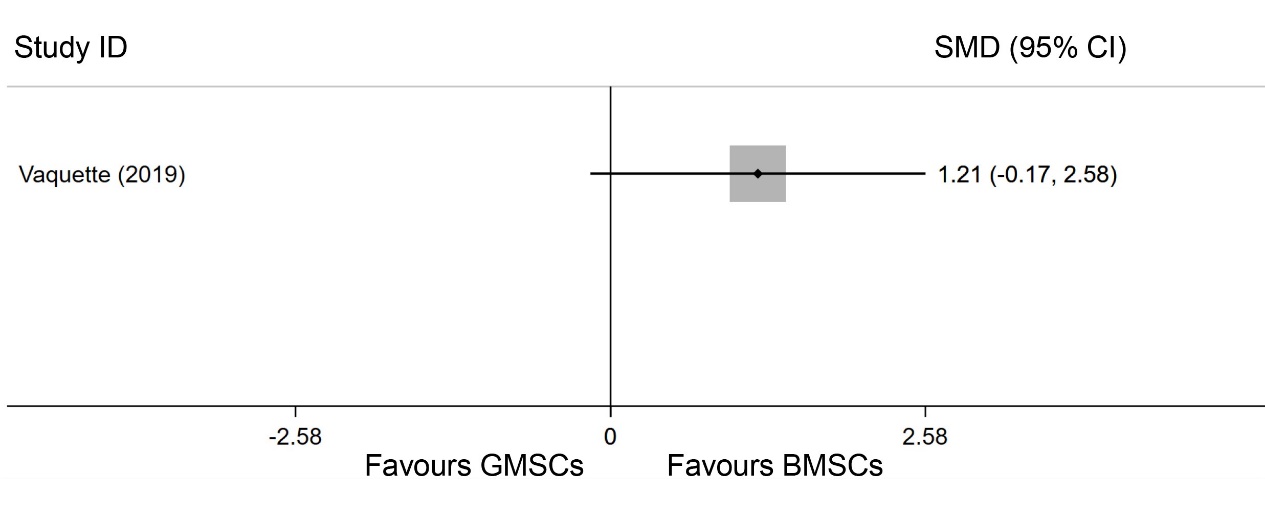
**

**Analysis 1.10 Comparison 10 BMSCs versus GMSCs, Outcome 1 NB.**

**Outcome 2 NC**

**
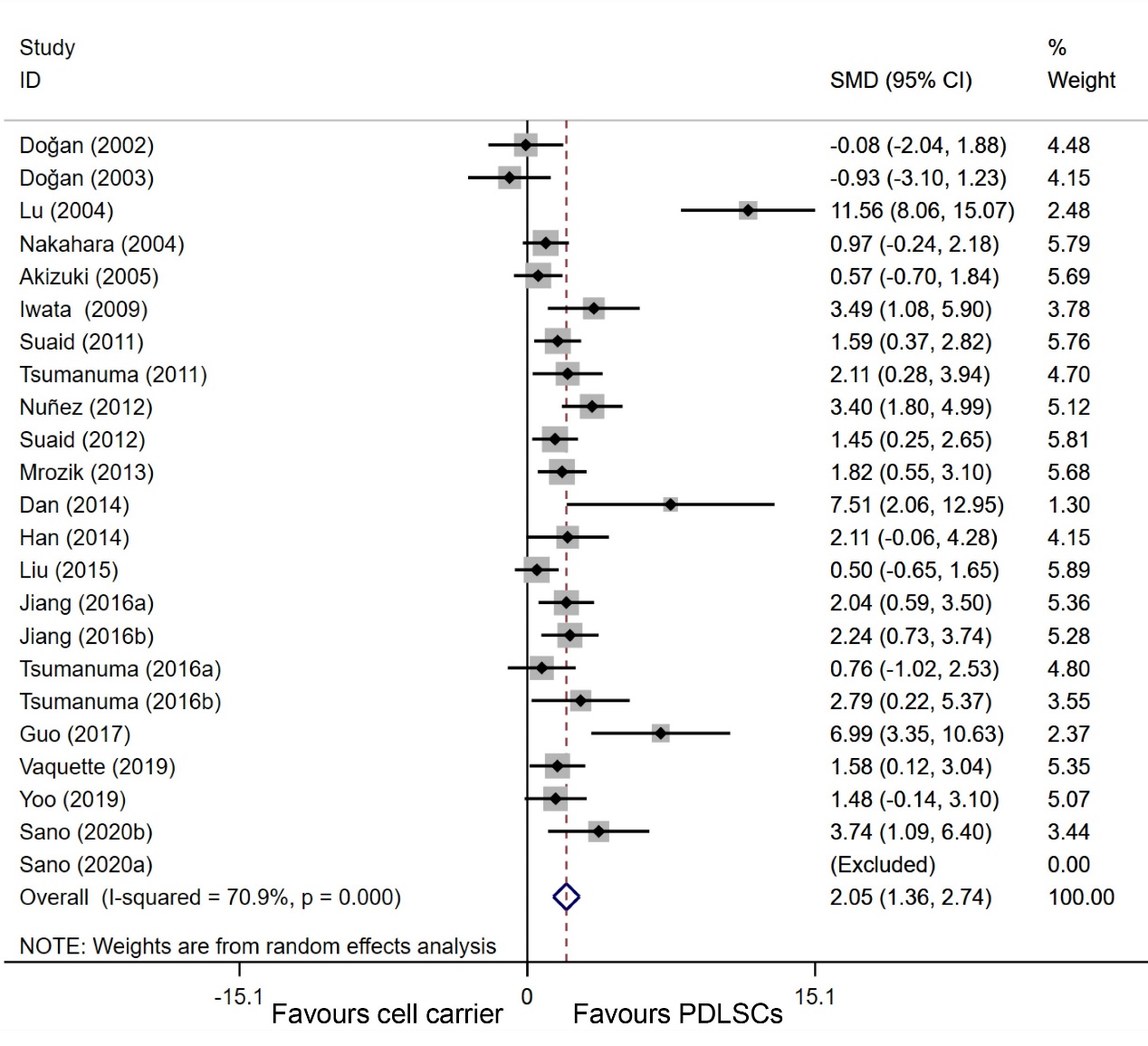
**

**Analysis 2.1 Comparison 11 PDLSCs versus cell carrier, Outcome 2 NC.**

**
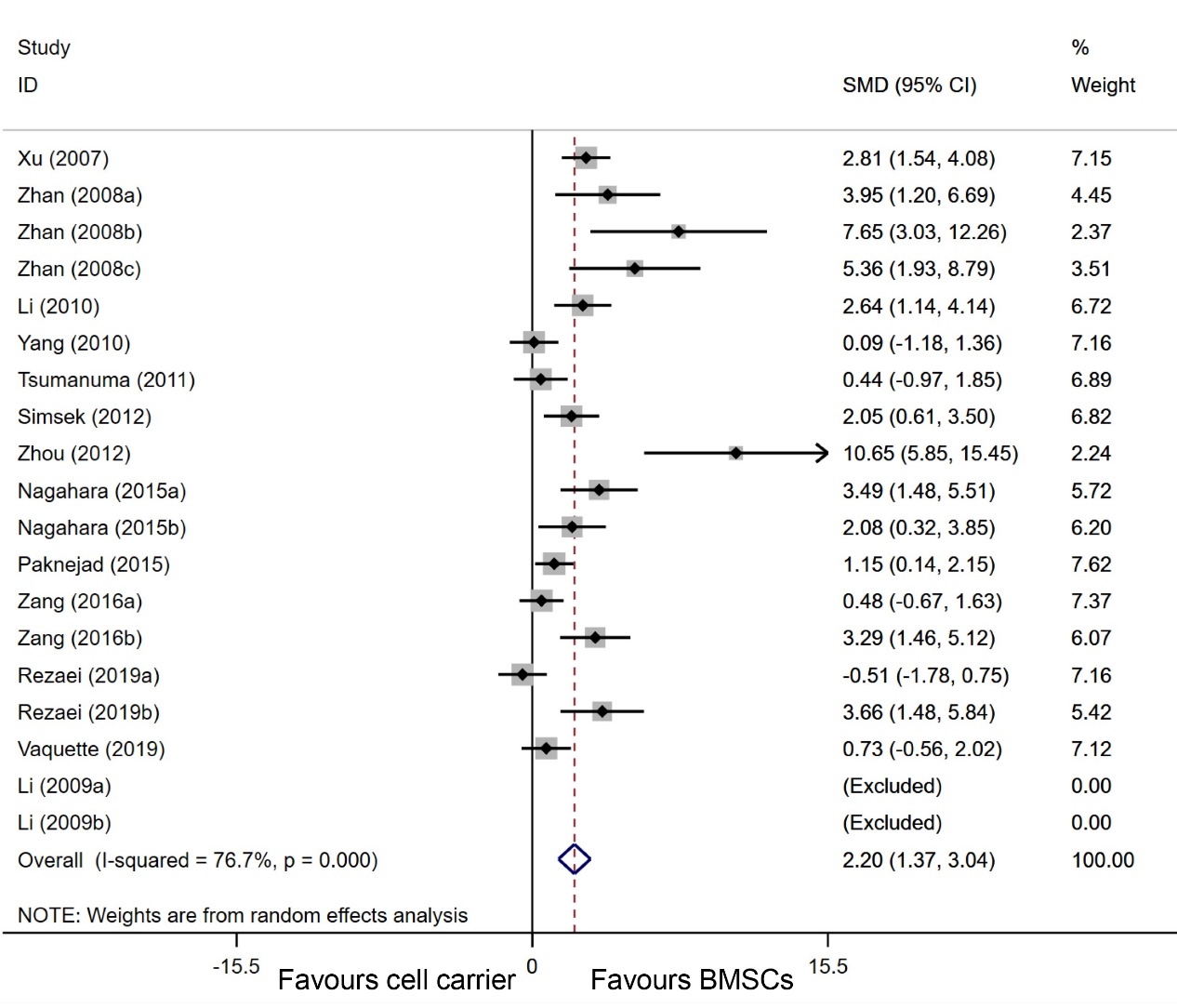
**

**Analysis 2.2 Comparison 12 BMSCs versus cell carrier, Outcome 2 NC.**

**
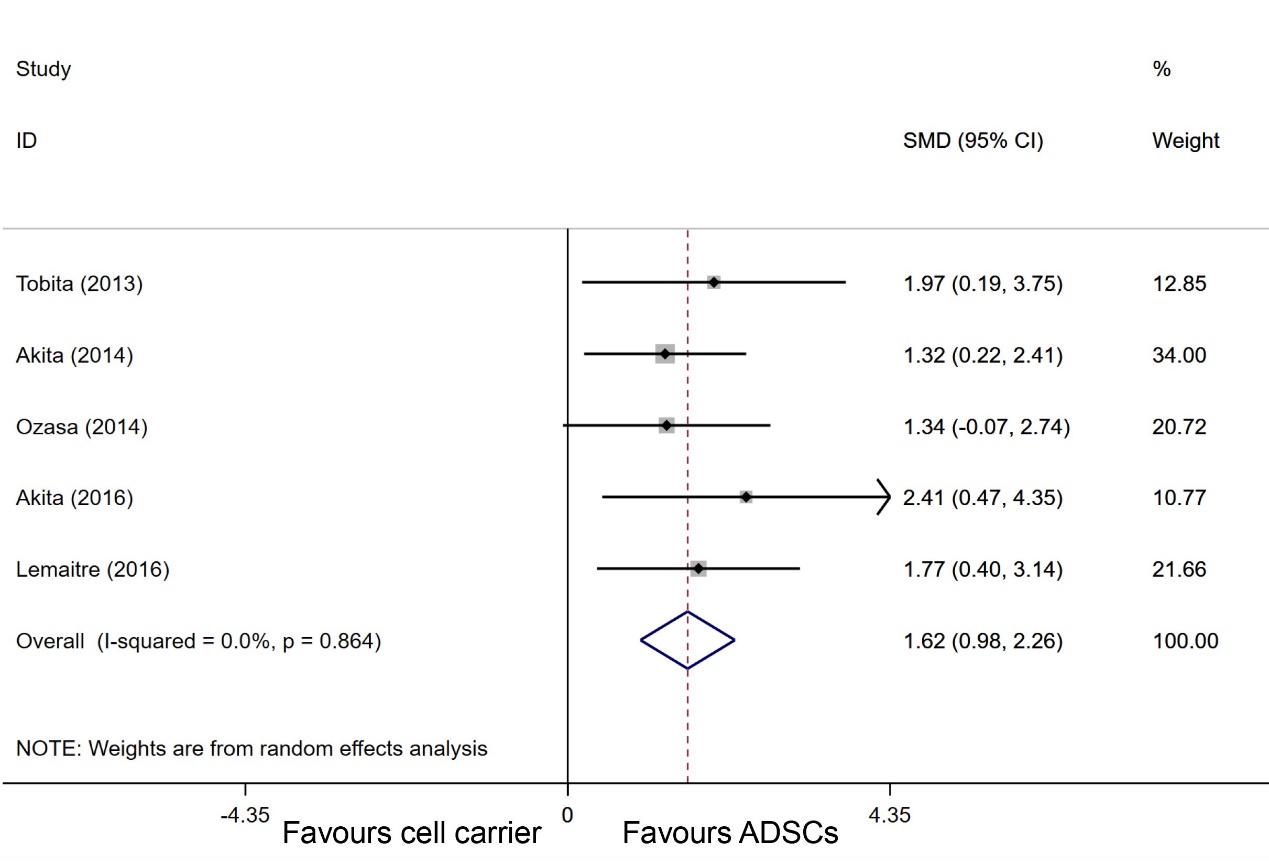
**

**Analysis 2.3 Comparison 13 ADSCs versus cell carrier, Outcome 2 NC.**

**
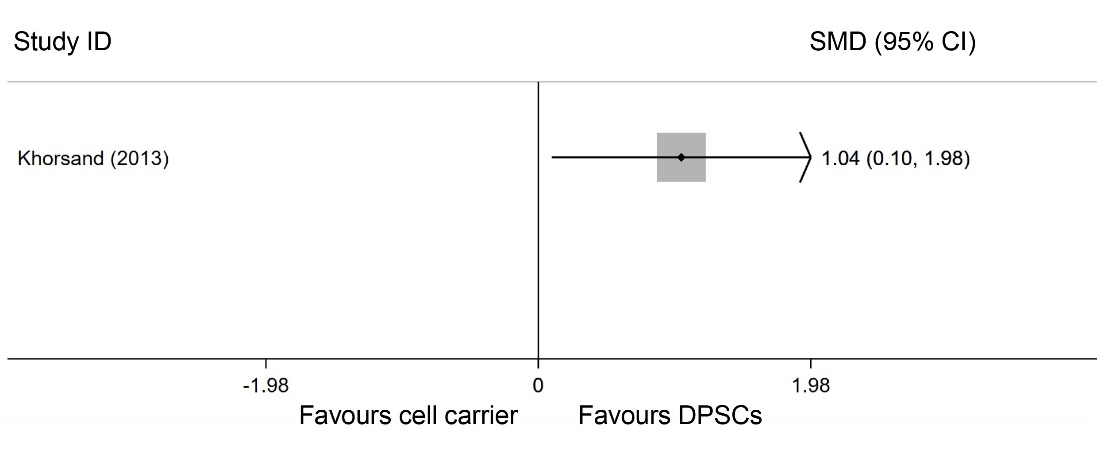
**

**Analysis 2.4 Comparison 14 DPSCs versus cell carrier, Outcome 2 NC.**

**
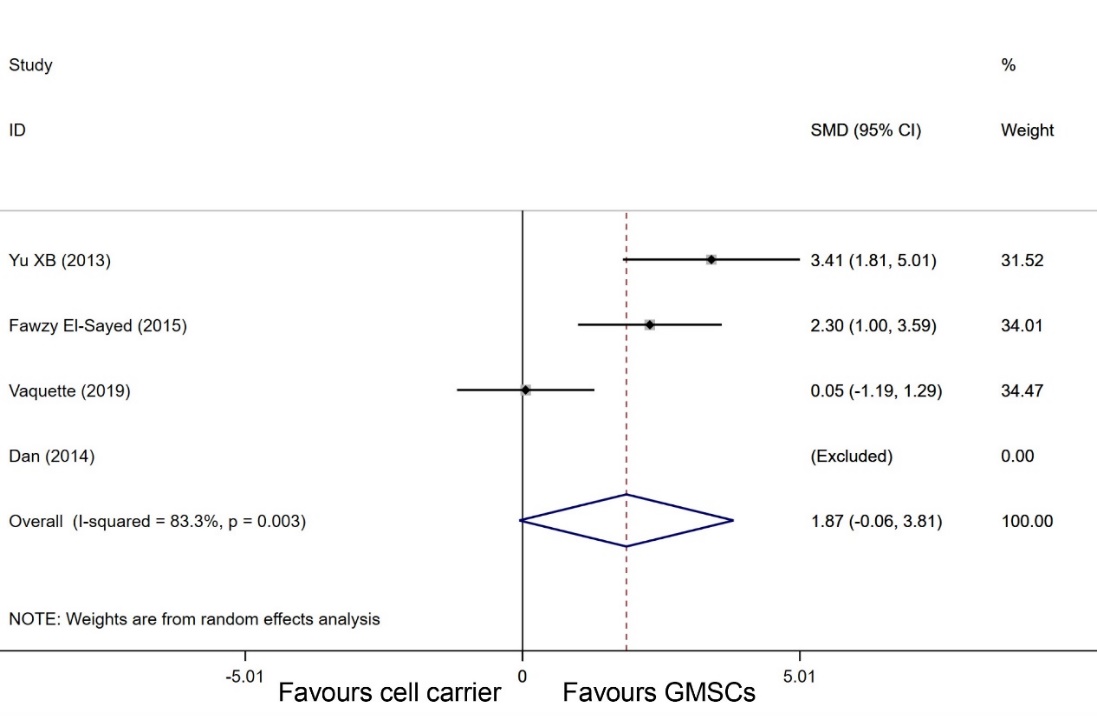
**

**Analysis 2.5 Comparison 15 GMSCs versus cell carrier, Outcome 2 NC.**

**
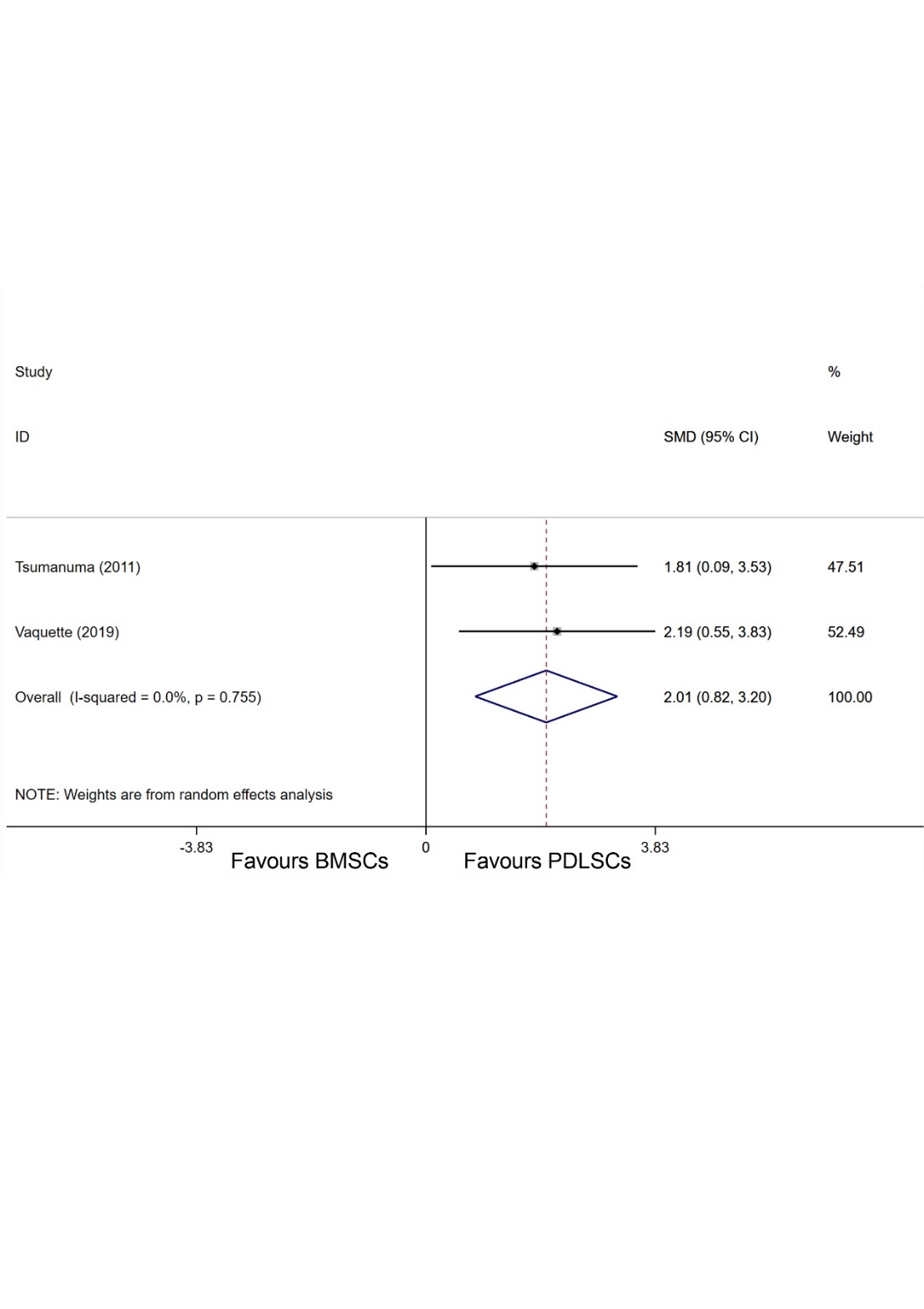
**

**Analysis 2.6 Comparison 16 PDLSCs versus BMSCs, Outcome 2 NC.**

**
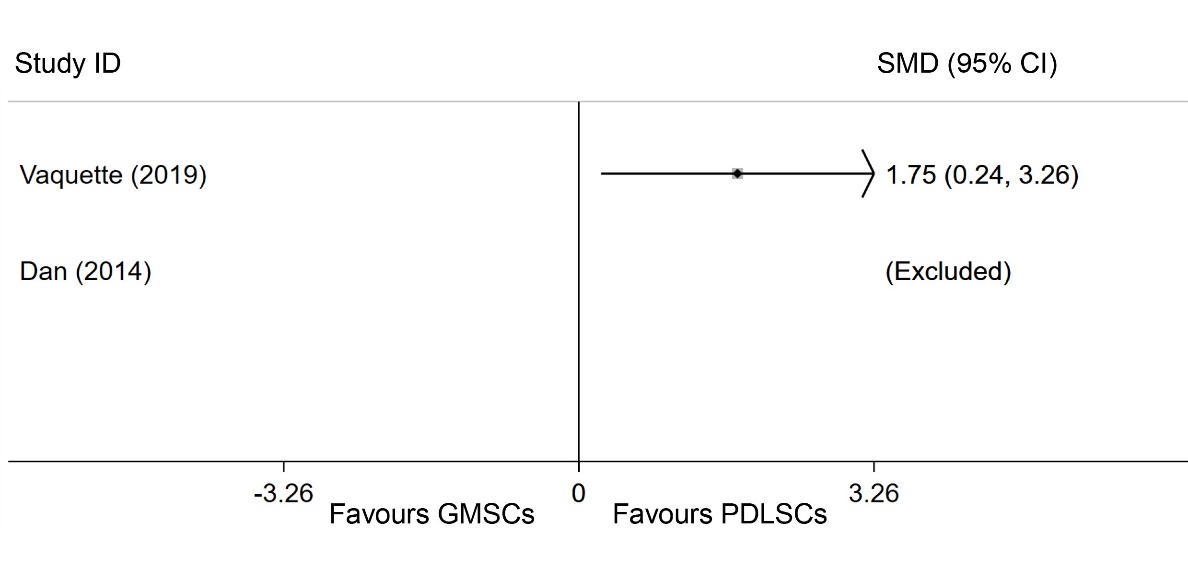
**

**Analysis 2.7 Comparison 17 PDLSCs versus GMSCs, Outcome 2 NC.**

**
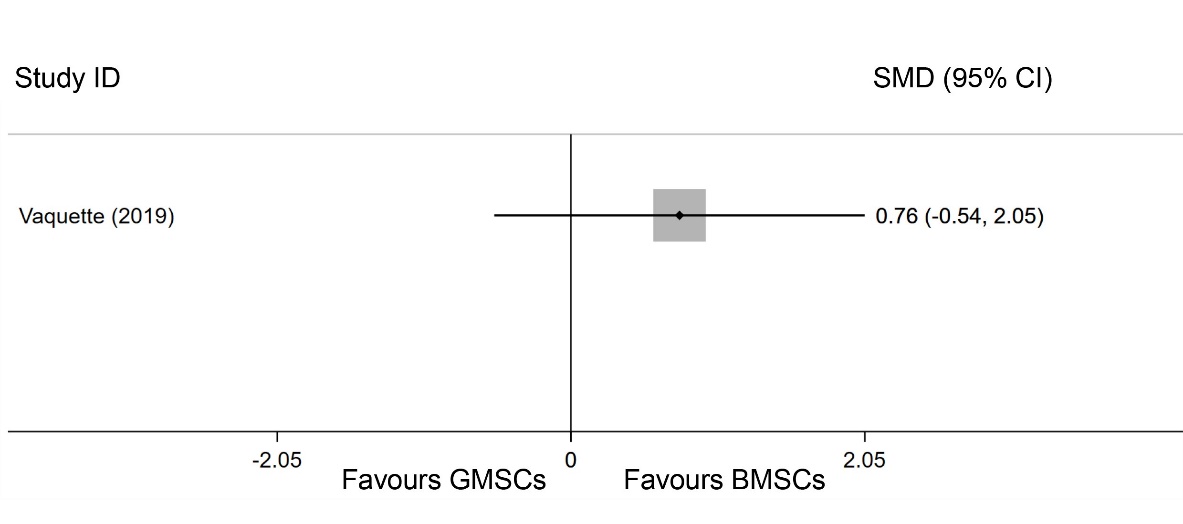
**

**Analysis 2.8 Comparison 18 BMSCs versus GMSCs, Outcome 2 NC.**

**Outcome 3 NPDL**

**
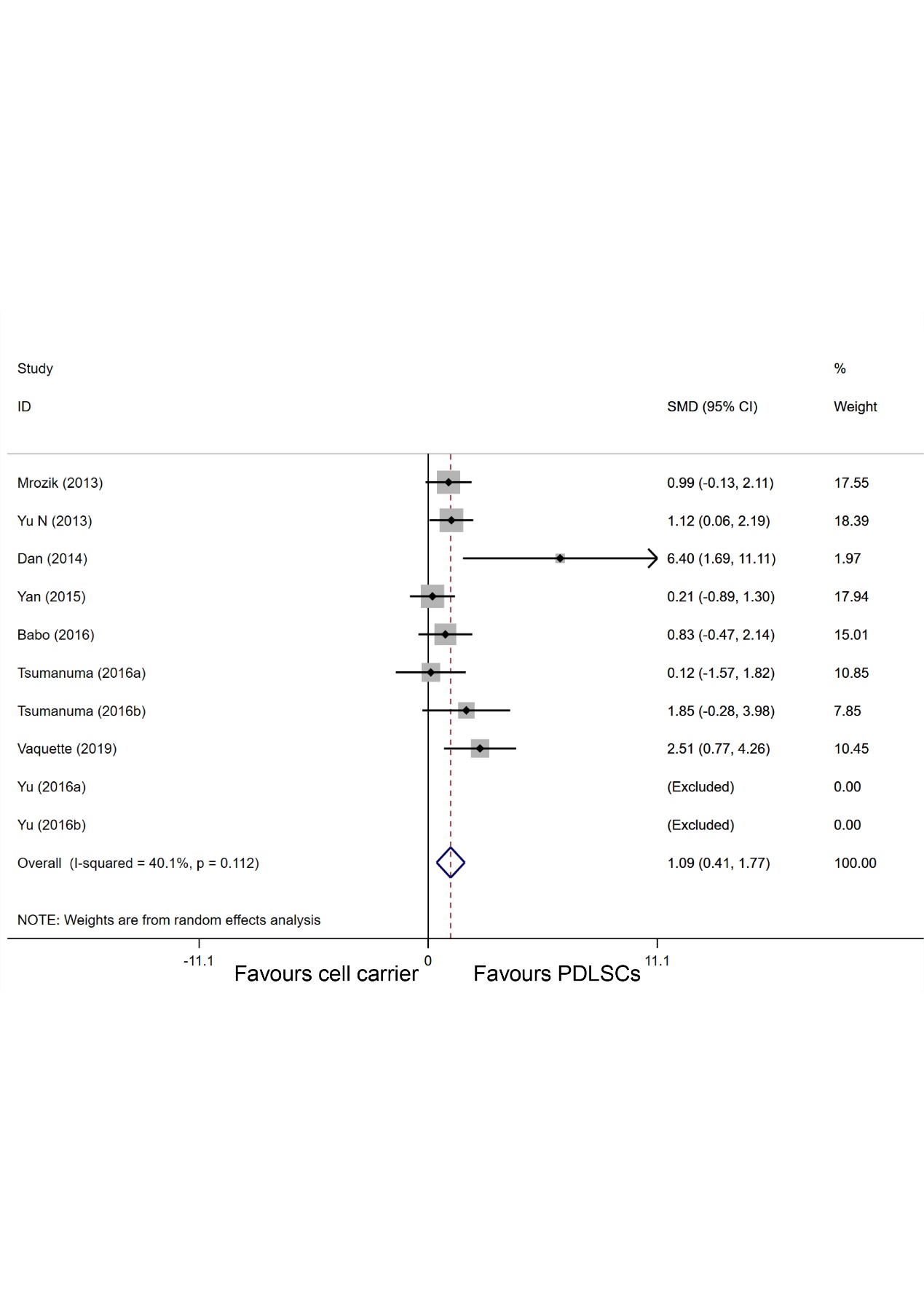
**

**Analysis 3.1 Comparison 19 PDLSCs versus cell carrier, Outcome 3 NPDL.**

**
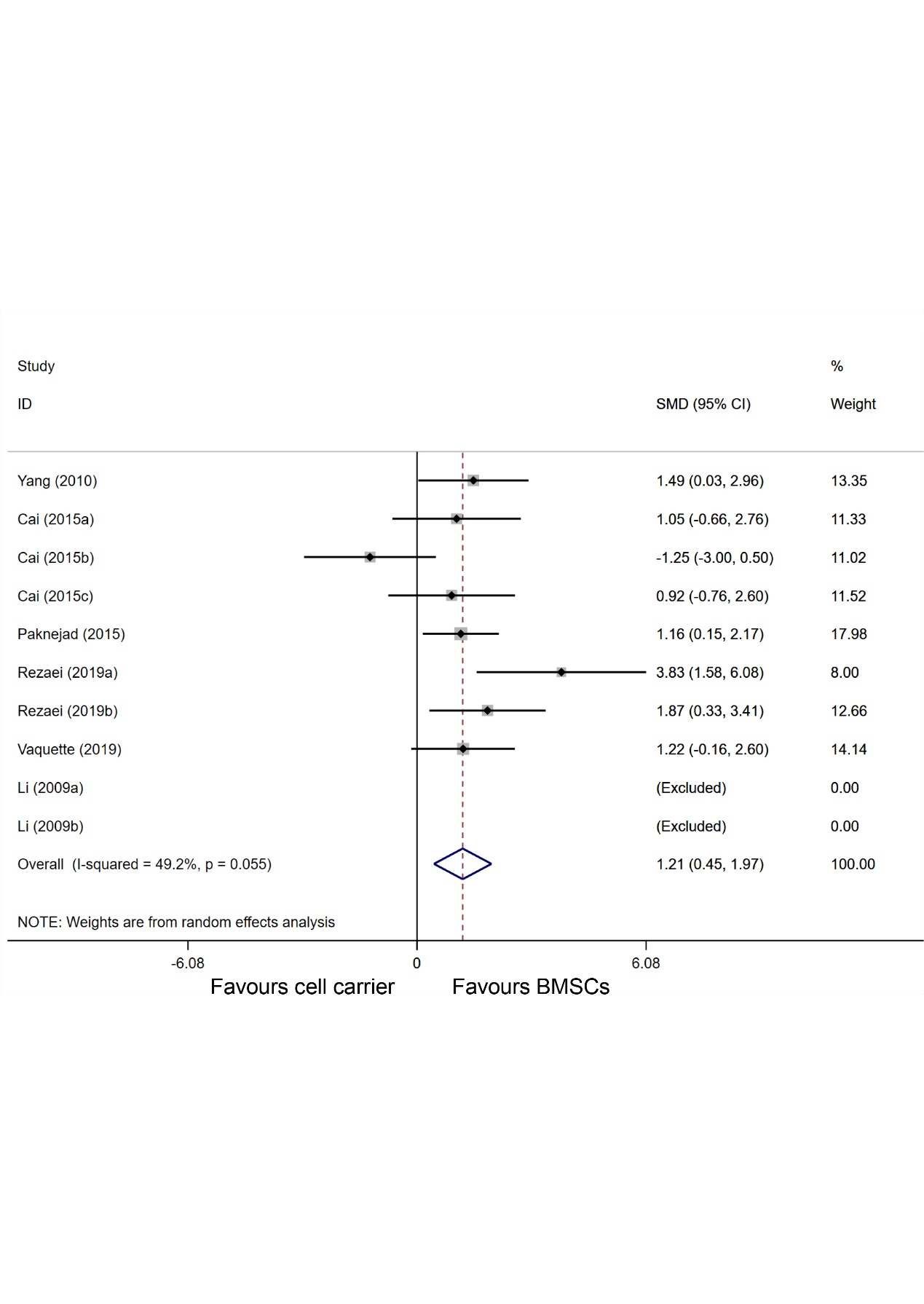
**

**Analysis 3.2 Comparison 20 BMSCs versus cell carrier, Outcome 3 NPDL.**

**
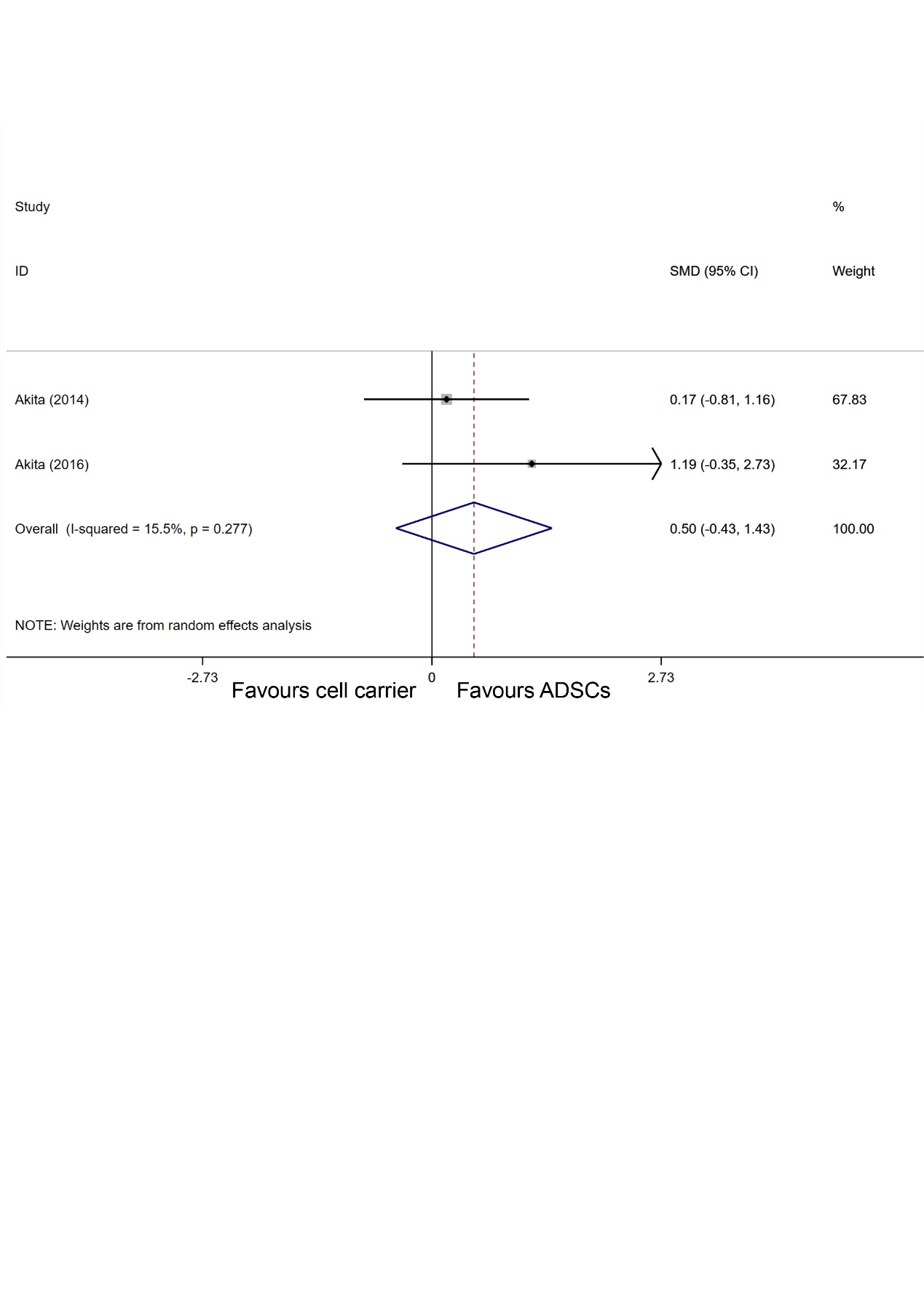
**

**Analysis 3.3 Comparison 20 ADSCs versus cell carrier, Outcome 3 NPDL.**

**
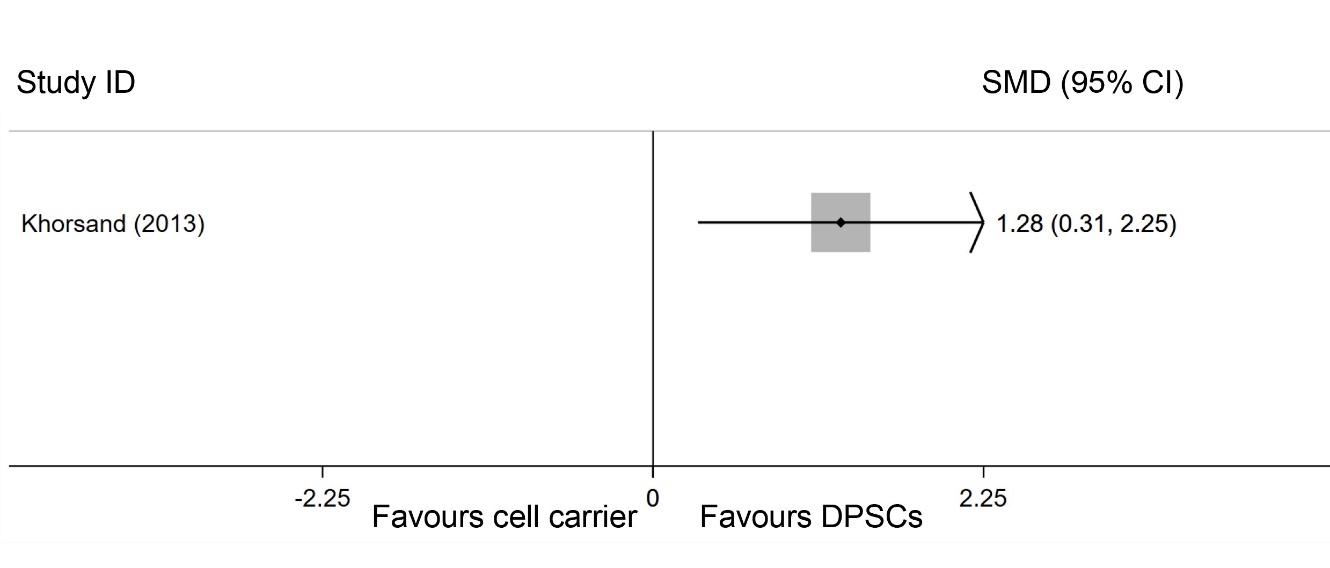
**

**Analysis 3.4 Comparison 21 DPSCs versus cell carrier, Outcome 3 NPDL.**

**
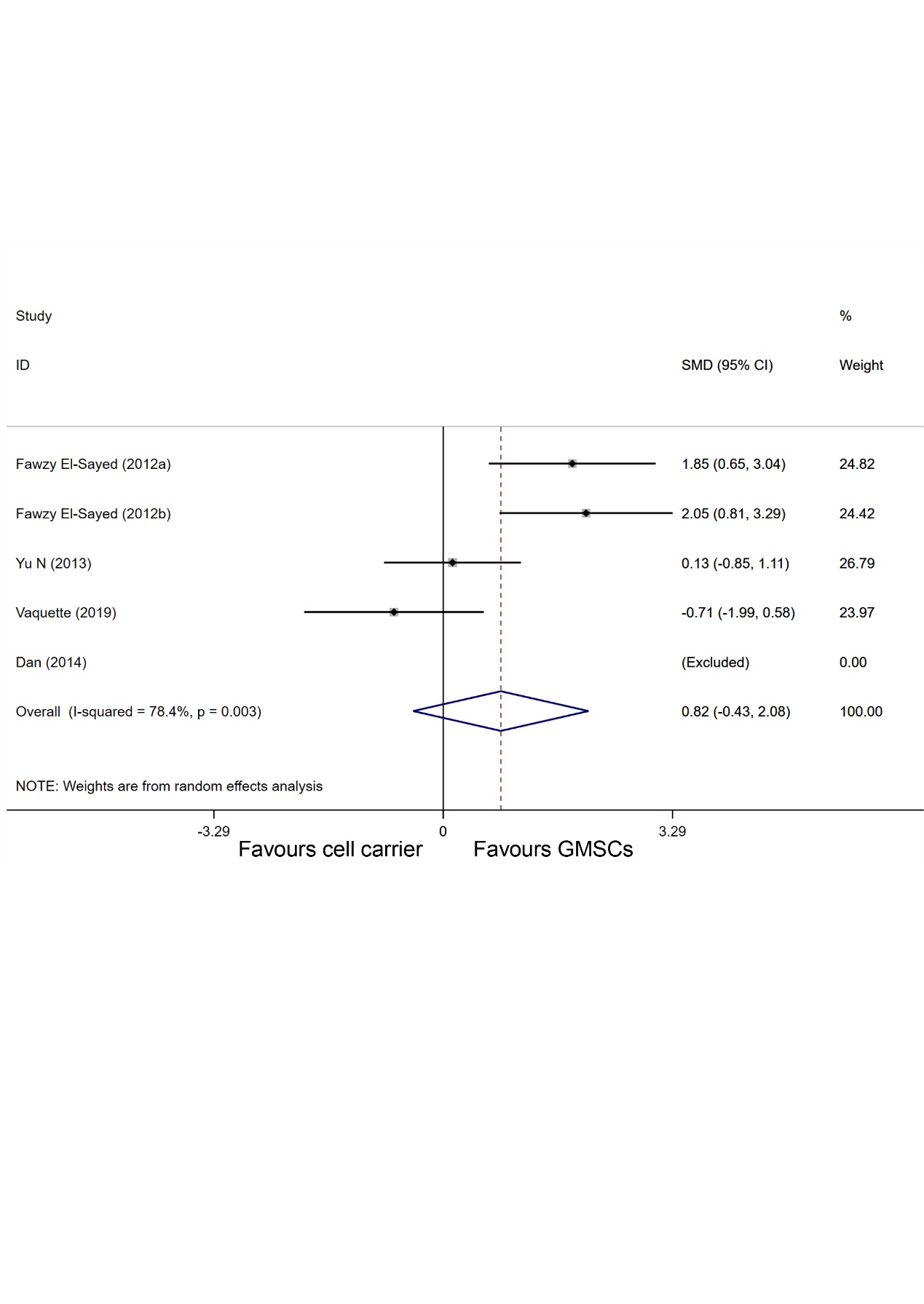
**

**Analysis 3.5 Comparison 22 GMSCs versus cell carrier, Outcome 3 NPDL.**

**
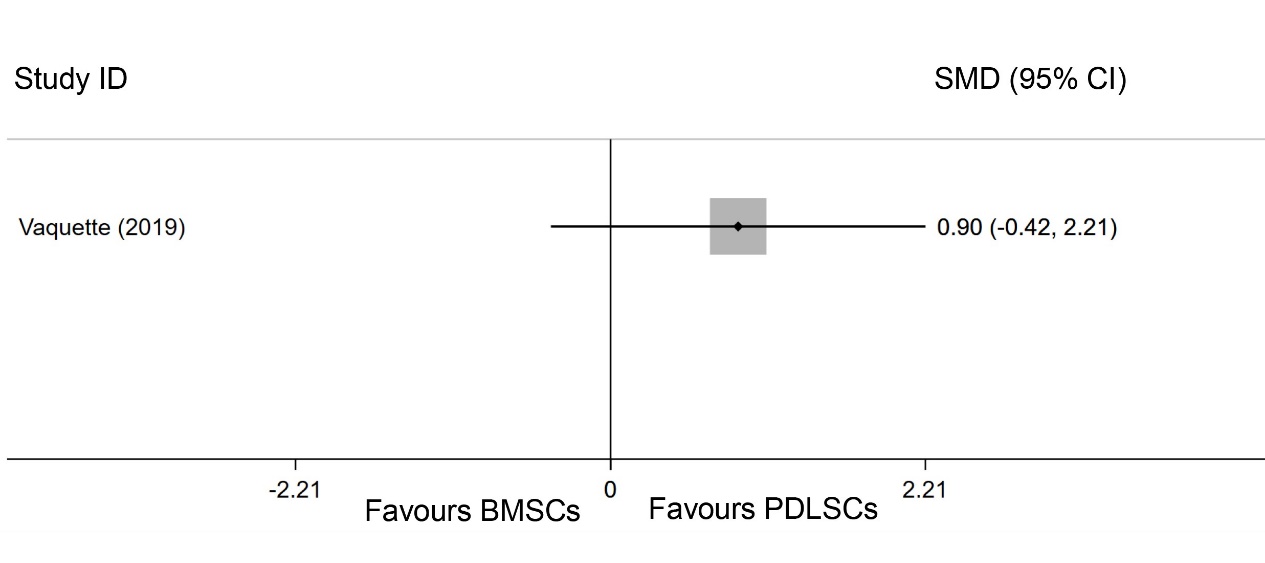
**

**Analysis 3.6 Comparison 23 PDLSCs versus BMSCs, Outcome 3 NPDL.**

**
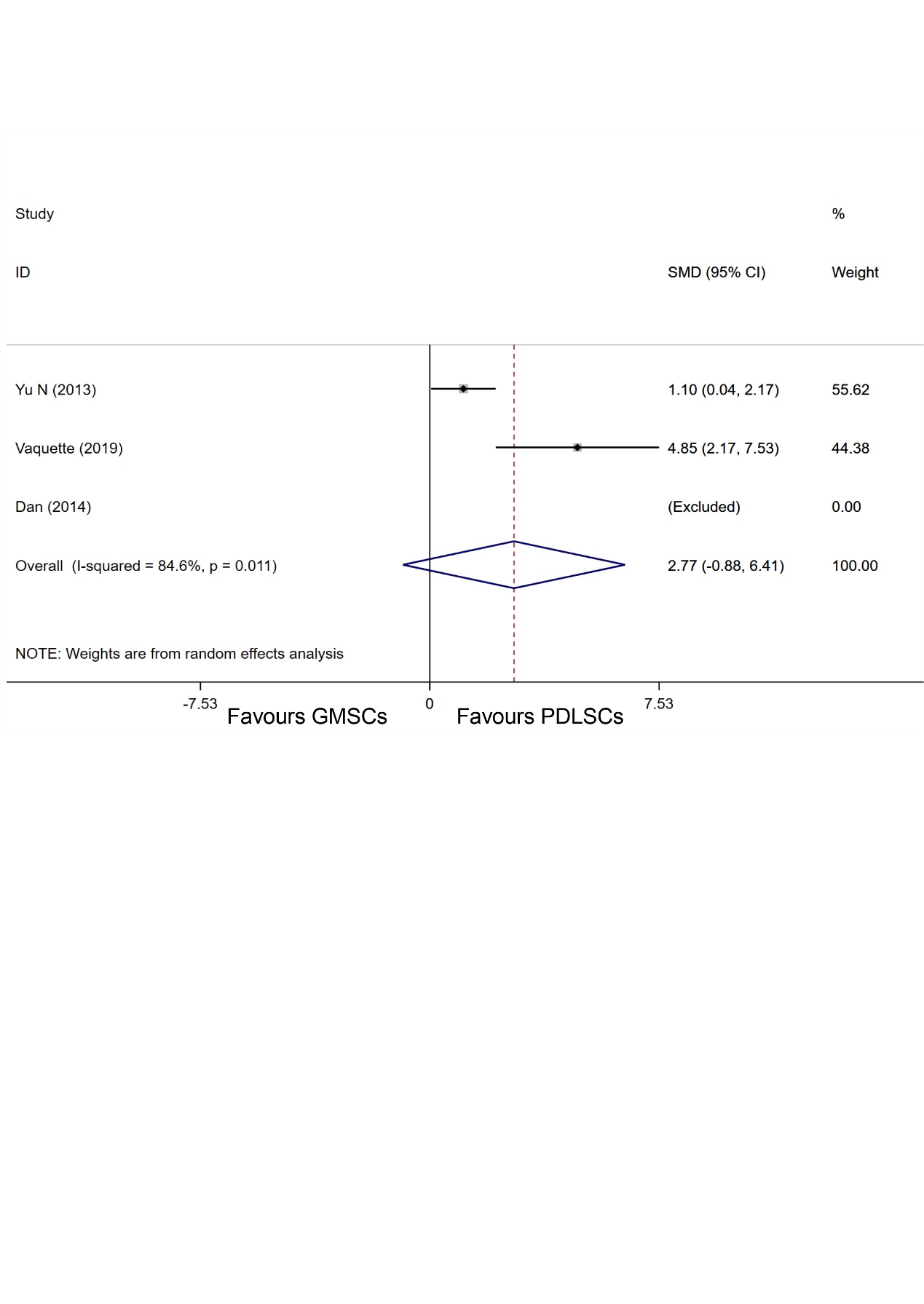
**

**Analysis 3.7 Comparison 24 PDLSCs versus GMSCs, Outcome 3 NPDL.**

**
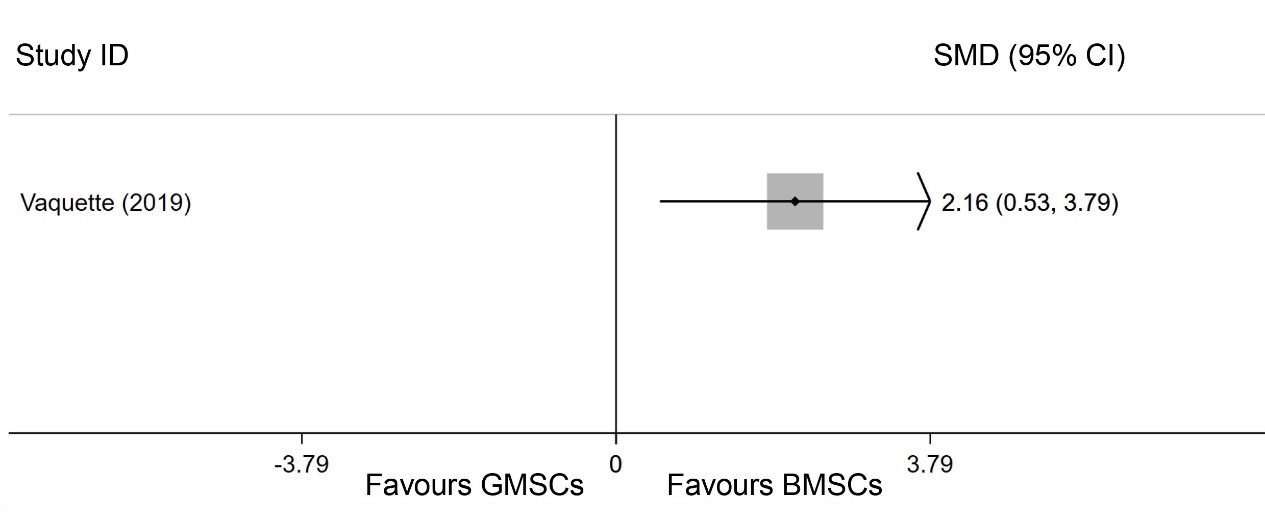
**

**Analysis 3.8 Comparison 25 BMSCs versus GMSCs, Outcome 3 NPDL.**
