# Supplementary material for: Stem cell therapies for periodontal tissue regeneration: a network meta-analysis of preclinical studies
Source: Stem Cell Res Ther. 2020 Oct 2;11:427. doi: 10.1186/s13287-020-01938-7 (PMC7531120; doi:10.1186/s13287-020-01938-7)
Supplement: Supplementary file 8 — Additional file 8. : Supplementary Table 6. Summary of the SUCRA, mean probabilities of being best and mean rank for each outcome. [file 13287_2020_1938_MOESM8_ESM.docx]

**Supplementary Table 6. Summary of the SUCRA, mean probabilities of being best and mean rank for each outcome.**


| Outcome 1 NB | | | |
| --- | --- | --- | --- |
| Treatment | SUCRA (%) | Mean PrBest (%) | Mean Rank |
| CCs | 7.4 | 0 | 5.6 |
| PDLSCs | 74.8 | 25.3 | 2.3 |
| BMSCs | 75.0 | 27.3 | 2.3 |
| ADSCs | 56.4 | 25.2 | 3.2 |
| DPSCs | 66.0 | 21.4 | 2.7 |
| GMSCs | 20.5 | 0.8 | 5.0 |
| Outcome 2 NC | | | |
| Treatment | SUCRA | Mean PrBest | Mean Rank |
| CCs | 6.3 | 0 | 5.7 |
| PDLSCs | 77.4 | 29.7 | 2.1 |
| BMSCs | 78.4 | 33.7 | 2.1 |
| ADSCs | 55.0 | 13 | 3.2 |
| DPSCs | 41.0 | 18 | 3.9 |
| GMSCs | 41.8 | 5.7 | 3.9 |
| Outcome 3 NPDL | | | |
| Treatment | SUCRA | Mean PrBest | Mean Rank |
| CCs | 11.3 | 0 | 5.4 |
| PDLSCs | 84.7 | 45.2 | 1.8 |
| BMSCs | 72.8 | 19.1 | 2.4 |
| ADSCs | 37.2 | 5.2 | 4.1 |
| DPSCs | 62.2 | 30.1 | 2.9 |
| GMSCs | 31.9 | 0.4 | 4.4 |

**Note:** Treatment hierarchies are presented with the SUCRA; the larger the SUCRA, the higher its rank among all available interventions. A SUCRA of 100% means the stem cell is the best and a SUCRA of 0% means the intervention is the worst.  **Abbreviations:** ADSCs, adipose tissue-derived stem cells; BMSCs, bone marrow-derived stem cells; CCs, cell carriers; DPSCs, dental pulp stem cells; GMSCs, gingival-derived stem cells; NB, newly formed bone; NC, newly formed cementum; NPDL, newly formed periodontal ligament; PDLSCs, periodontal ligament stem cells; PrBest, probabilities of being best; SUCRA, surface under the cumulative ranking curve.
