# Supplementary material for: Stem cell therapies for periodontal tissue regeneration: a network meta-analysis of preclinical studies
Source: Stem Cell Res Ther. 2020 Oct 2;11:427. doi: 10.1186/s13287-020-01938-7 (PMC7531120; doi:10.1186/s13287-020-01938-7)
Supplement: Supplementary file 9 — Additional file 9. : Supplementary Table 7. The global and local inconsistency between direct and indirect sources of evidence. [file 13287_2020_1938_MOESM9_ESM.docx]

**Supplementary Table 7. The global and local inconsistency between direct and indirect sources of evidence.**

**Table S7A. Testing for inconsistency globally.**

| Outcomes | chi^2^ | Prob > chi^2^ |
| --- | --- | --- |
| NB | 10.23 | 0.5095 |
| NC | 10.82 | 0.1469 |
| NPDL | 7.05 | 0.2169 |

**Table S7B. The results of node‐splitting between direct and indirect evidence.**

Outcome 1 NB

| Comparisons | Direct | | Indirect | | Difference | | P>z | tau |
| --- | --- | --- | --- | --- | --- | --- | --- | --- |
|  | Coef. | Std. Err. | Coef. | Std. Err. | Coef. | Std. Err. |  |  |
| CC vs PDLSCs * | 1.76 | 0.33 | 4.04 | 1.43 | -2.28 | 1.46 | 0.12 | 1.50 |
| CC vs BMSCs * | 1.86 | 0.38 | 2.34 | 1.73 | -0.48 | 1.77 | 0.79 | 1.57 |
| CC vs ADSCs | … | … | … | … | … | … | … | … |
| CC vs DPSCs * | 1.95 | 0.57 | -0.72 | 1.85 | 2.67 | 1.96 | 0.17 | 1.53 |
| CC vs GMSCs * | 0.09 | 0.79 | 1.84 | 1.69 | -1.75 | 1.85 | 0.34 | 1.57 |
| PDLSCs vs BMSCs | 0.10 | 1.21 | -0.02 | 0.51 | 0.13 | 1.31 | 0.92 | 1.57 |
| PDLSCs vs DPSCs | -1.72 | 1.21 | 0.33 | 0.68 | -2.05 | 1.40 | 0.14 | 1.53 |
| PDLSCs vs GMSCs | -1.59 | 1.02 | -1.40 | 1.08 | -0.19 | 1.46 | 0.90 | 1.57 |
| BMSCs vs DPSCs | 0.92 | 1.72 | -0.35 | 0.68 | 1.27 | 1.85 | 0.49 | 1.55 |
| BMSCs vs GMSCs | -1.33 | 1.71 | -1.54 | 0.89 | 0.22 | 1.93 | 0.91 | 1.57 |

Outcome 2 NC

| Comparisons | Direct | | Indirect | | Difference | | P>z | tau |
| --- | --- | --- | --- | --- | --- | --- | --- | --- |
|  | Coef. | Std. Err. | Coef. | Std. Err. | Coef. | Std. Err. |  |  |
| CC vs PDLSCs * | 1.92 | 0.32 | 6.25 | 1.66 | -4.33 | 1.66 | **0.01** | 1.20 |
| CC vs BMSCs * | 2.16 | 0.45 | 1.53 | 1.90 | 0.63 | 1.95 | 0.75 | 1.40 |
| CC vs ADSCs | … | … | … | … | … | … | … | … |
| CC vs DPSCs | … | … | … | … | … | … | … | … |
| CC vs GMSCs * | 1.23 | 0.79 | 0.61 | 2.17 | 0.62 | 2.28 | 0.79 | 1.40 |
| PDLSCs vs BMSCs | -1.31 | 1.08 | 0.36 | 0.56 | -1.67 | 1.22 | 0.17 | 1.34 |
| PDLSCs vs GMSCs | -2.87 | 1.35 | 0.26 | 1.00 | -3.12 | 1.73 | **0.07** | 1.31 |
| ADSCs vs GMSCs | -0.69 | 1.55 | -1.09 | 1.03 | 0.40 | 1.86 | 0.83 | 1.41 |

Outcome 3 NPDL

| Comparisons | Direct | | Indirect | | Difference | | P>z | tau |
| --- | --- | --- | --- | --- | --- | --- | --- | --- |
|  | Coef. | Std. Err. | Coef. | Std. Err. | Coef. | Std. Err. |  |  |
| CC vs PDLSCs* | 1.52 | 0.38 | 4.14 | 1.43 | -2.62 | 1.45 | **0.07** | 0.83 |
| CC vs BMSCs* | 1.41 | 0.47 | 1.81 | 1.84 | -0.40 | 1.90 | 0.83 | 1.00 |
| CC vs ADSCs | … | … | … | … | … | … | … | … |
| CC vs DPSCs | … | … | … | … | … | … | … | … |
| CC vs GMSCs* | 0.59 | 0.51 | -0.98 | 1.45 | 1.57 | 1.53 | 0.30 | 0.95 |
| PDLSCs vs BMSCs | -0.90 | 1.19 | -0.11 | 0.64 | -0.80 | 1.35 | 0.56 | 0.99 |
| PDLSCs vs GMSCs | -2.24 | 0.70 | 0.18 | 0.75 | -2.41 | 1.02 | **0.02** | 0.73 |
| BMSCs vs GMSCs | -1.87 | 1.18 | -0.64 | 0.74 | -1.22 | 1.39 | 0.38 | 0.94 |

**Table S7C. Evaluation of inconsistency using loop-specific heterogeneity estimates.**

Outcome 1 NB

| Loop | IF | se IF | z_value | p_value | 95% CI | Loop_Heterog_tau2 |
| --- | --- | --- | --- | --- | --- | --- |
| PDLSCs-BMSCs-DPSCs | 3.267 | 3.202 | 1.020 | 0.308 | (0.00,9.54) | 3.776 |
| CC-PDLSCs-DPSCs | 2.596 | 1.840 | 1.411 | 0.158 | (0.00,6.20) | 2.146 |
| CC-BMSCs-DPSCs | 0.863 | 1.486 | 0.581 | 0.561 | (0.00,3.78) | 0.977 |
| PDLSCs-BMSCs-GMSCs | 0.655 | 0.975 | 0.672 | 0.501 | (0.00,2.57) | 0 |
| CC-BMSCs-GMSCs | 0.364 | 1.437 | 0.253 | 0.800 | (0.00,3.18) | 0.853 |
| CC-PDLSCs-GMSCs | 0.094 | 1.884 | 0.050 | 0.960 | (0.00,3.79) | 2.424 |
| CC-PDLSCs-BMSCs | 0.009 | 1.437 | 0.006 | 0.995 | (0.00,2.82) | 1.565 |

Outcome 2 NC

| Loop | IF | se IF | z_value | p_value | 95% CI | Loop_Heterog_tau2 |
| --- | --- | --- | --- | --- | --- | --- |
| CC-PDLSCs- GMSCs | 1.829 | 2.085 | 0.877 | 0.380 | (0.00,5.91) | 1.871 |
| CC-PDLSCs-BMSCs | 1.424 | 1.842 | 0.773 | 0.439 | (0.00,5.03) | 2.165 |
| CC-BMSCs-GMSCs | 0.743 | 3.321 | 0.224 | 0.823 | (0.00,7.25) | 3.076 |
| PDLSCs-BMSCs-GMSCs | 0.638 | 1.262 | 0.506 | 0.613 | (0.00,3.11) | 0 |

Outcome 3 NPDL

| Loop | IF | se IF | z_value | p_value | 95% CI | Loop_Heterog_tau2 |
| --- | --- | --- | --- | --- | --- | --- |
| PDLSCs-BMSCs-DPSCs | 1.649 | 1.117 | 1.475 | 0.140 | (0.00,3.84) | 0 |
| CC-PDLSCs-DPSCs | 1.455 | 1.33 | 1.094 | 0.274 | (0.00,4.06) | 1.266 |
| CC-PDLSCs-BMSCs | 0.885 | 2.594 | 0.341 | 0.733 | (0.00,5.97) | 1.813 |
| CC-BMSCs-DPSCs | 0.782 | 3.35 | 0.233 | 0.815 | (0.00,7.35) | 2.364 |

* Note: all the evidence about these contrasts comes from the trials which directly compare them.

**Abbreviations:** ADSCs, adipose tissue-derived stem cells; BMSCs, bone marrow-derived stem cells; CC, cell carrier; CI, confidence interval; DPSCs, dental pulp stem cells; GMSCs, gingival-derived stem cells; IF, inconsistency factor; NB, newly formed bone; NC, newly formed cementum; NPDL, newly formed periodontal ligament; PDLSCs, periodontal ligament stem cells.
