# Supplementary material for: Stem cell therapies for periodontal tissue regeneration: a network meta-analysis of preclinical studies
Source: Stem Cell Res Ther. 2020 Oct 2;11:427. doi: 10.1186/s13287-020-01938-7 (PMC7531120; doi:10.1186/s13287-020-01938-7)
Supplement: Supplementary file 10 — Additional file 10. : Supplementary Fig. 3. Evaluation of inconsistency using loop-specific heterogeneity estimates. When at least three interventions are compared with each other in a network that forms a closed path, the loop-specific approach compares indirect evidence with direct evidence, and their differences define the inconsistency factor (IF). The magnitude of the IF, 95% CI of IF, and a loop-specific z-test can be used to infer the presence of inconsistency in each loop. IF close to zero indicates that direct evidence and indirect evidence are very consistent. [file 13287_2020_1938_MOESM10_ESM.docx]

**Supplementary Figure 3.** **Evaluation of inconsistency using loop-specific heterogeneity estimates.** When at least three interventions are compared with each other in a network that forms a closed path, the loop-specific approach compares indirect evidence with direct evidence, and their differences define the inconsistency factor (IF). The magnitude of the IF, 95% CI of IF, and a loop-specific z-test can be used to infer the presence of inconsistency in each loop. IF close to zero indicates that direct evidence and indirect evidence are very consistent.


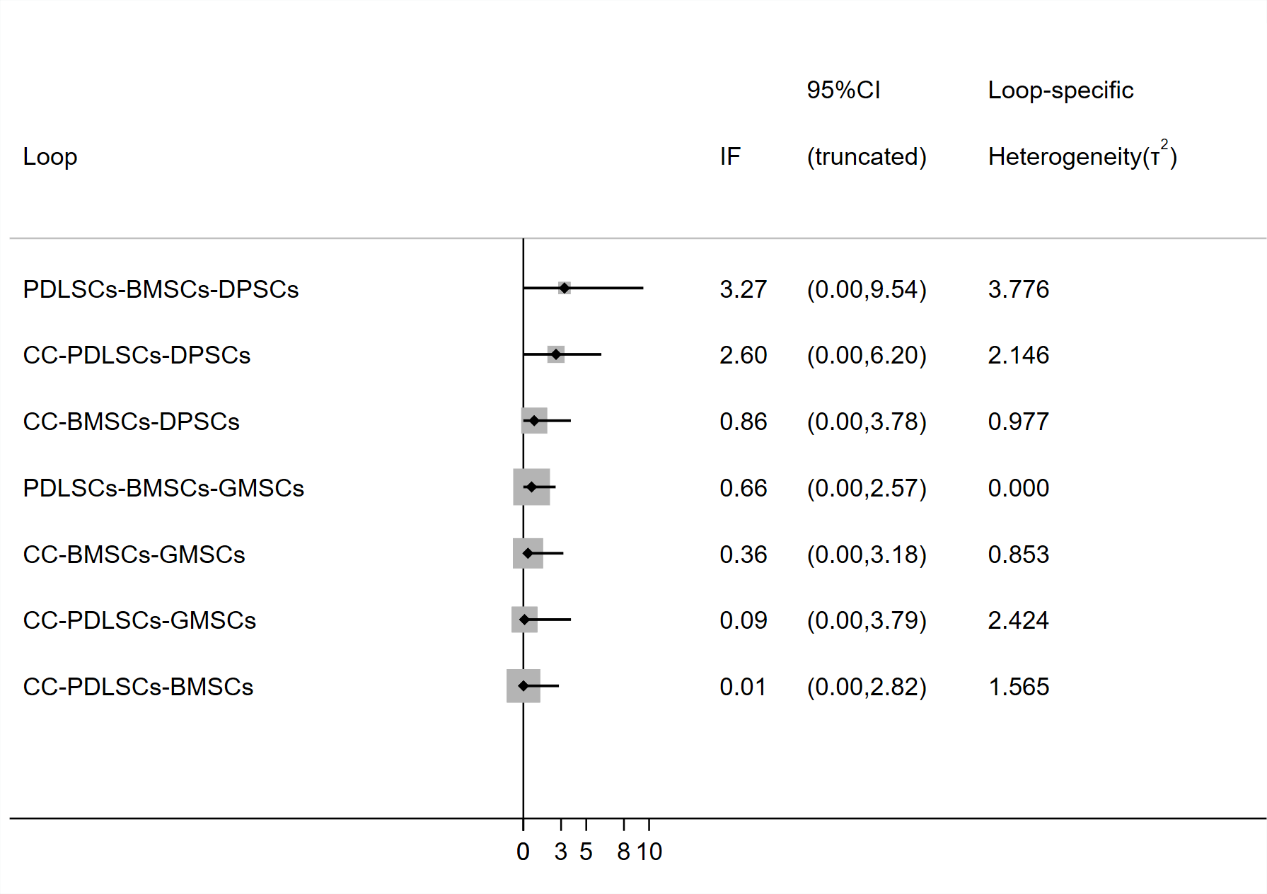


**Figure S3A.** Evaluation of inconsistency using loop-specific heterogeneity estimates for NB.


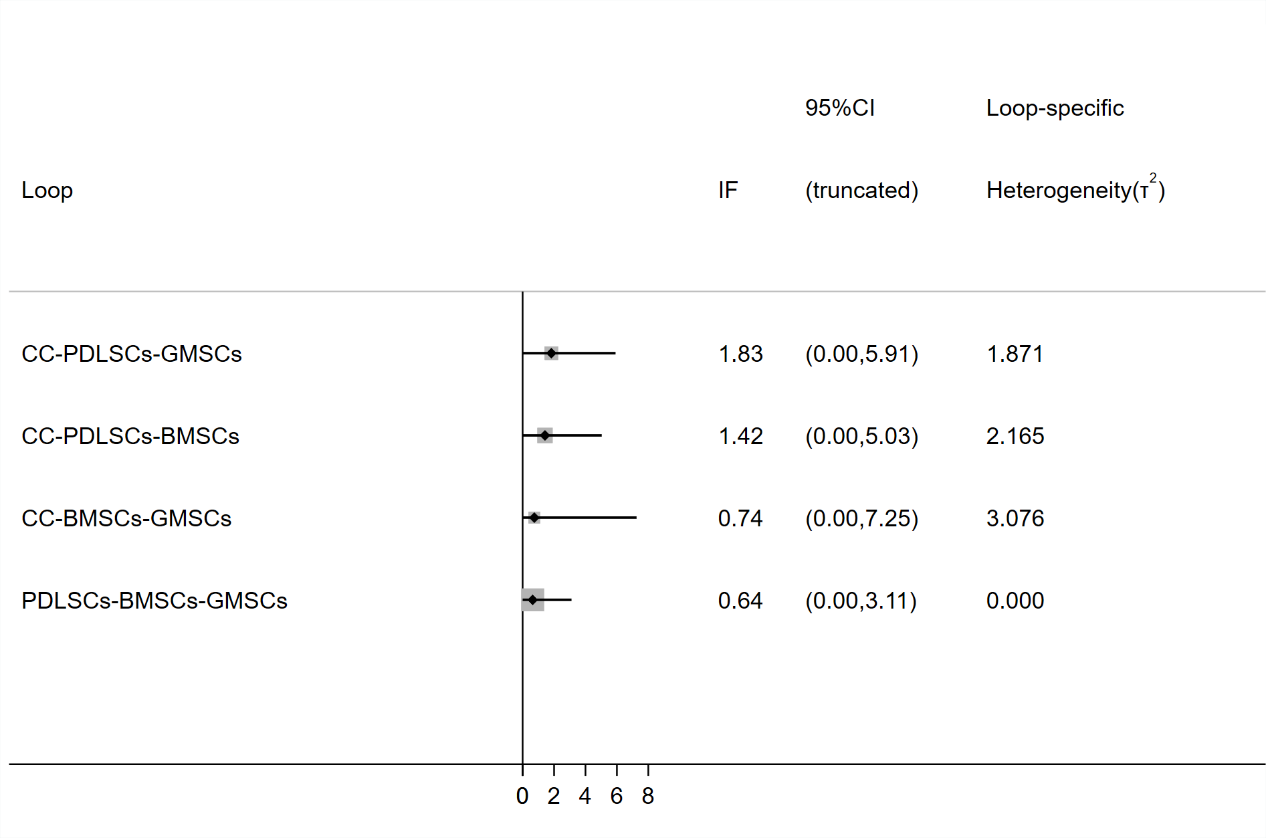


**Figure S3B.** Evaluation of inconsistency using loop-specific heterogeneity estimates for NC.


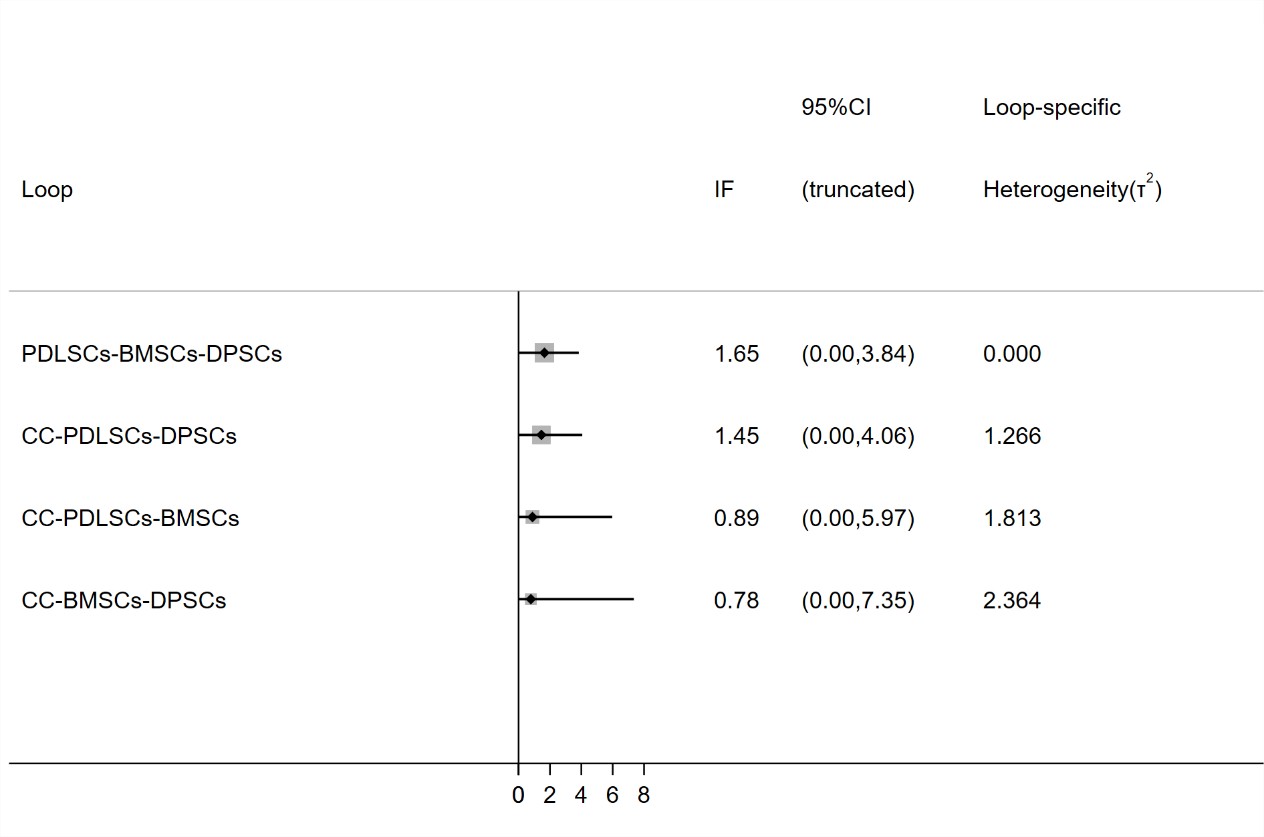


**Figure S3C.** Evaluation of inconsistency using loop-specific heterogeneity estimates for NPDL.

**Abbreviations:** ADSCs, adipose tissue-derived stem cells; BMSCs, bone marrow-derived stem cells; CC, cell carrier; CI, confidence interval; DPSCs, dental pulp stem cells; GMSCs, gingival-derived stem cells; IF, inconsistency factor; NB, newly formed bone; NC, newly formed cementum; NPDL, newly formed periodontal ligament; PDLSCs, periodontal ligament stem cells.
